# Gut Microbiome Compositional and Functional Signatures Associated with Chemotherapy Response in Pancreatic Ductal Adenocarcinoma: An Exploratory Cohort Study

**DOI:** 10.3390/metabo16100720

**Published:** 2026-09-28

**Authors:** Erica Pizzocaro, Matteo Soverini, Gian Luca Salvagno, Ashkan Lotfollahzadeh, Barbara Santacroce, Giulia Bruno, Isabella Frigerio, Laura Di Renzo, Andrea Castagnetti, Giovanni Butturini

**Affiliations:** 1 HPB Unit, General Surgery Department, Pederzoli Hospital, Peschiera del Garda, 37019 Verona, Italy; 2 PhD School of Applied Medical-Surgical Sciences, University of Rome Tor Vergata, 00133 Rome, Italy; 3 Wellmicro Srl, 40138 Bologna, Italy; 4 Clinical Biochemistry Section, Department of Engineering for Innovation Medicine, University of Verona, 37129 Verona, Italy; 5 Department of Biochemistry, Pederzoli Hospital, Peschiera del Garda, 37019 Verona, Italy; 6 Collegium Medicum, Spoleczna Akademia Nauk University, 90-113 Łódź, Poland; 7 Section of Clinical Nutrition and Nutrigenomic, Department of Biomedicine and Prevention, University of Rome Tor Vergata, 00133 Rome, Italy

**Keywords:** pancreatic cancer, gut microbiome, butyrate, chemotherapy response, surgical eligibility, metagenomics, oral pathobionts, exploratory cohort

## Abstract

**Highlights:**

**What are the main findings?**

- In this exploratory cohort (*n* = 15), PDAC patients who achieved surgical eligibility after chemotherapy showed enrichment of *Collinsella aerofaciens* and *Butyrivibrio fibrisolvens*, while non-responders were enriched in multiple oral-origin taxa (*Streptococcus gordonii*, *Actinomyces* spp., *Rothia dentocariosa*).
- Responders showed broadly elevated predicted SCFAs (butyrate had the largest, though non-significant, effect size; *p* = 0.179) and bile acids; these associations were, however, confounded by the uneven distribution of disease stage and chemother-apy regimen between groups.

**What are the implications of the main findings?**

- These hypothesis-generating results suggest that baseline gut-microbial metabolic competence may contribute to chemotherapy response in PDAC.
- The associations require prospective validation in larger, regimen-stratified cohorts before any predictive or clinical use.

**Abstract:**

**Background/Objectives:** The gut microbiome modulates chemotherapy efficacy in pancreatic ductal adenocarcinoma (PDAC), yet its relationship with the clinically relevant endpoint of post-chemotherapy surgical eligibility remains poorly characterised. We explored whether baseline gut microbiome composition and metagenomically predicted metabolic function differ between chemotherapy responders and non-responders in PDAC. **Methods:** In this prospective single-centre exploratory cohort study, faecal samples were collected from 15 patients with non-immediately resectable PDAC at diagnosis, before chemotherapy. Whole-genome shotgun metagenomics was performed, and microbial functional capacity was inferred through an assembly-based pipeline. Patients were classified as responders (achieved surgical eligibility, *n* = 6) or non-responders (*n* = 9). **Results:** At baseline, responders showed enrichment of *Collinsella aerofaciens* (*p* = 0.026) and *Butyrivibrio fibrisolvens* (*p* = 0.012), while non-responders were enriched in oral-origin bacteria (*Streptococcus gordonii*, *Actinomyces* spp., *Rothia dentocariosa*). Predicted butyrate showed the largest effect size difference between groups but did not reach significance (*p* = 0.179); responders also had broadly elevated predicted SCFAs and bile acids. However, chemotherapy regimen (mFOLFIRINOX in 5/6 responders vs. 2/9 non-responders; Fisher *p* = 0.041) and disease stage were unevenly distributed between groups. **Conclusions:** This exploratory study identifies the compositional and functional microbiome features distinguishing PDAC chemotherapy responders from non-responders. These associations are confounded by disease stage and regimen and require validation in larger, regimen-stratified cohorts before predictive or mechanistic claims can be made.

## 1. Introduction

Pancreatic ductal adenocarcinoma (PDAC) is among the most lethal human malignancies, with a 5-year survival rate below 12% [1,2]. Neoadjuvant or induction chemotherapy represents the standard of care for borderline resectable and locally advanced disease, yet tumour response allowing surgical resection is achieved in less than 50% of cases [3,4]. Tumour genetics alone cannot explain this variability [5], prompting a search for host-extrinsic factors that modulate treatment efficacy.

The gut microbiome has emerged as a compelling candidate. Tintelnot et al. demonstrated that the microbiota-derived tryptophan metabolite indole-3-acetic acid (3-IAA) was enriched in PDAC patients responding to FOLFIRINOX, enhancing chemotherapy efficacy through a neutrophil myeloperoxidase-dependent mechanism involving reactive oxygen species accumulation in tumour cells [6]. This finding established that microbial metabolites can directly influence the pharmacological action of cytotoxic agents in PDAC. Independently, a consistent pattern of gut dysbiosis has been documented in PDAC, characterised by depletion of short-chain fatty acid (SCFA)-producing bacteria, including *Faecalibacterium*, *Eubacterium*, and *Bifidobacterium* [7,8,9], and enrichment of lipopolysaccharide-producing and oral-origin taxa [8,10]. Oral pathobionts such as *Porphyromonas gingivalis*, *Streptococcus* spp., and *Fusobacterium nucleatum* have been detected in pancreatic tumour tissue itself, and their presence in the gut may reflect oro-intestinal translocation facilitated by disrupted mucosal barriers [11,12,13].

Among microbial metabolites, butyrate has attracted particular attention for its pleiotropic anti-tumoral properties. Butyrate inhibits histone deacetylases (HDACs), leading to upregulation of the tumour suppressor p53 and increased chemosensitivity in cancers harbouring p53 mutations [14,15]. It enhances anti-tumour CD8+ T-cell function through epigenetic modulation of T-cell receptor signalling [16,17], maintains intestinal epithelial barrier integrity during chemotherapy-induced mucosal injury [18], and has demonstrated synergistic effects with gemcitabine in preclinical PDAC models [19]. Furthermore, butyrate modulates the tumour microenvironment by suppressing pro-inflammatory IL-6 signalling in macrophages, thereby counteracting the immunosuppressive milieu characteristic of PDAC [20].

Despite these mechanistic insights, clinical data linking the pre-treatment gut microbiome to the endpoint of surgical eligibility following chemotherapy remain sparse. Here, we report an exploratory analysis of gut microbiome composition and metagenomically predicted metabolic function in a prospective cohort of PDAC patients profiled at diagnosis, comparing those who subsequently achieved surgical eligibility with those who did not.

## 2. Materials and Methods

### 2.1. Study Design and Participants

Patients affected by histologically confirmed PDAC, not immediately resectable according to the NCCN Clinical Practice Guidelines in Oncology for Pancreatic Adenocarcinoma [21], and candidates for chemotherapy were prospectively enrolled at the HPB Surgical Unit of Ospedale P. Pederzoli. Faecal samples were collected at diagnosis before starting chemotherapy, which was scheduled at the medical oncologist’s discretion. Patients were re-evaluated by the same surgical team after a full course of chemotherapy. The definition of responder or not was based upon the eligibility to exploratory laparotomy and has been achieved in those patients in whom, after imaging and laboratory re-staging, the neoplasm became resectable. As regards imaging, we applied the common RECIST criteria to assess response. As regards laboratory, we obtained the measures of oncological markers commonly altered in pancreatic ductal carcinoma (CEA and Ca19/9). The study was approved by CET nr.41029 on 20 August 2024 and all participants provided written informed consent.

### 2.2. DNA Extraction, Library Preparation, and Sequencing

Faecal samples were collected using the Copan SMART eNAT kit (Copan Italia S.p.A., Brescia, Italy), which preserves nucleic acid integrity. DNA was extracted following a protocol developed by Wellmicro Srl. Briefly, faecal material was mechanically lysed using inert beads and the FastPrep homogenizer (MP Biomedicals, Irvine, CA, USA). DNA extraction and purification were performed using the Mag-Bind Universal Pathogen Kit (Omega Bio-Tek, Norcross, GA, USA) on the automated NIMBUS Presto platform (Hamilton, Reno, NV, USA). Microbial DNA was quantified using the Qubit 1X dsDNA Broad Range Assay Kit (Thermo Fisher Scientific, Waltham, MA, USA) on the Synergy instrument (Agilent Technologies, Santa Clara, CA, USA) and normalised to 13.3 ng/µL.

Metagenomic libraries were prepared using the Illumina DNA Prep kit (Illumina, San Diego, CA, USA) [22] following the manufacturer’s protocol adapted for the VANTAGE automated platform (Hamilton). Library fragment size was assessed by capillary electrophoresis on the QIAxcel instrument (Qiagen, Hilden, Germany). Libraries were quantified with the Qubit 1X dsDNA High Sensitivity Assay Kit (Thermo Fisher Scientific), normalised to 4 nM, pooled, and sequenced on the NovaSeq 6000Dx instrument (Illumina), generating 2 × 150 bp paired-end reads. Raw reads were quality-filtered using fastp (v1.3.6) [23]. After filtering, base quality was high (approximately 93% of bases ≥ Q30; mean Phred quality ~Q35). All samples were processed in a single sequencing batch on the same instrument, minimising potential batch effects; mean sequencing depth was 5.3 ± 0.6 million read pairs per sample, and no samples were excluded for quality reasons.

### 2.3. Taxonomic Profiling

Taxonomic classification was performed using Kraken2 (v2.1.6) [24] against a curated proprietary reference database (WMDB2.0, Wellmicro Srl, Bologna, Italy) integrating bacterial, archaeal, viral, and eukaryotic reference genomes with harmonised NCBI taxonomy, at a confidence threshold of 0.30. Species-level relative abundances were calculated from classified read counts normalised to total classified reads per sample.

### 2.4. Functional Metabolite Prediction

Quality-filtered reads were assembled de novo using MEGAHIT (v1.2.9) [25] (k-min = 75, k-max = 151, step = 28). Contigs larger than 300 bp were retained and open reading frames (ORFs) predicted using FragGeneScan (v1.32) [26] (illumina_5 error model). Predicted protein sequences were aligned against a curated functional protein database (WMF_DB, Wellmicro Srl) using DIAMOND BLASTp (v2.2.2) [27] (minimum identity 70%), yielding an Enzyme Commission (EC) abundance matrix per sample. The bioinformatic pipeline is composed entirely of open-source, peer-reviewed tools (fastp, MEGAHIT, FragGeneScan and DIAMOND BLASTp), each independently reproducible; the only proprietary component is the curated protein reference database (WMF_DB), analogous to the role of curated reference databases in other widely used metagenomic annotation frameworks (e.g., ChocoPhlAn for MetaPhlAn, UniRef90 for HUMAnN3, and custom genome databases for Kraken2).

Functional profiles were integrated to derive metabolite-level signatures using a validated proprietary inference framework (Wellmicro Srl), summarising microbial metabolic potential at both individual and pathway-informed levels. The framework has been validated on the same independent dataset used in the MelonnPan publication [28] (validation data available upon request); as the two methods differ fundamentally in their inference strategy, this constitutes a validation of predictive coverage on a shared benchmark dataset rather than a head-to-head comparison. Only metabolites of established or probable microbial origin, as curated from the MetaCyc metabolic pathway database and cross-referenced with AGORA2 genome-scale metabolic reconstructions, were retained for analysis. Individual metabolites were further classified into functional categories (SCFAs and fermentation products, bile acids, tryptophan/indole metabolism, phenolic/aromatic metabolites, biogenic amines, amino acids, vitamins, organic acids, and others; full classification in Supplementary Table S1). For interpretability, selected functional outputs were summarised as pathway-level scores representing major microbial metabolic outputs (e.g., butyrate, acetate, propionate, secondary bile acids). The EC-to-metabolite mapping table is provided as Supplementary Table S2.

### 2.5. Statistical Analysis

Differential abundance between responders and non-responders was assessed by Wilcoxon rank-sum test with Benjamini–Hochberg correction. Effect sizes were reported as log2 fold-change (pseudocount 1 × 10^−5^) and median difference. Species markers were identified using repeated random forest classification (ranger package v0.18.0, 120 iterations with stratified 75%/25% train/test splits, permutation importance averaged across iterations; no independent validation set was used and results are reported as exploratory). A multivariable logistic regression (GLM, binomial family) was fitted with surgical eligibility as outcome and predicted butyrate score plus disease stage (III vs. IV) as predictors; model comparison used AIC and McFadden pseudo-R^2^. With only 15 observations and 6 events, the guideline of approximately 10 events per predictor limits the model to at most two covariates. The association between chemotherapy regimen and response was tested by Fisher’s exact test. Candidate clinical covariates (age, body mass index, CA19-9, and nutritional and inflammatory indices) were compared univariately between responders and non-responders (Supplementary Table S9). All analyses used R v4.6.1.

## 3. Results

### 3.1. Population and Potential Confounders

Fifteen patients were enrolled. Six patients (6/15, 40%) underwent successful surgical resection and were identified as responders; nine (9/15, 60%) were non-responders. The chemotherapy regimen differed between groups: mFOLFIRINOX was administered to 5/6 (83%) responders but only 2/9 (22%) non-responders, while gemcitabine plus nab-paclitaxel was the most common regimen in non-responders (6/9, 67%). This imbalance was statistically significant (Fisher exact *p* = 0.041) and represents an important potential confounder, as mFOLFIRINOX is associated with higher response rates than gemcitabine-based regimens in PDAC [3,4]. Furthermore, disease stage was unevenly distributed: stage III was more prevalent among responders (Table S3), and stage III patients were more likely to receive mFOLFIRINOX. Thus, stage, regimen, and outcome are partially concatenated in this cohort, and the microbiome differences described below cannot be unambiguously attributed to intrinsic treatment response independently of these clinical determinants.

Baseline clinical and laboratory characteristics are summarised in Table S3. PERMANOVA analysis confirmed that T0 species composition did not differ significantly by subsequently assigned chemotherapy regimen (R^2^ = 0.08, *p* = 0.51; Supplementary Table S8), indicating that the pre-treatment microbiome was not detectably influenced by the factors that guided regimen selection.

### 3.2. Compositional Differences Between Responders and Non-Responders

Wilcoxon rank-sum testing of species-level relative abundances at T0 identified several species that differed nominally between groups (Figure 1; full results in Table S4).

Among responders, *Collinsella aerofaciens* showed the most robust enrichment (relative abundance 17.2% vs. 0.5% in non-responders, *p* = 0.026, prevalence 100% in both groups), representing a biologically plausible and technically reliable signal. *C. aerofaciens* is a bile acid-metabolising actinobacterium with established roles in bile salt deconjugation and SCFA cross-feeding [29]. *Butyrivibrio fibrisolvens*, one of the most efficient butyrate producers in the human gut via the butyryl-CoA:acetate CoA-transferase pathway [30], was also nominally enriched in responders (*p* = 0.012). However, *B. fibrisolvens* was present at very low abundance (median 0.0002% in responders vs undetectable in non-responders) with a prevalence of approximately 50%, raising concerns about the robustness of species-level identification at the limit of metagenomic detection. After Benjamini–Hochberg correction, no individual species survived FDR correction; all associations are therefore reported at nominal significance as exploratory findings.

Non-responders showed enrichment of multiple oral-origin species: *Streptococcus gordonii*, a commensal of the oral cavity and established early coloniser in dental plaque biofilms; *Actinomyces* species (*A.* sp. oral taxon 169, *A. viscosus*, *A. oris*); and *Rothia dentocariosa*. These are well-characterised members of the oral microbiome whose faecal presence has been described as consistent with oro-intestinal translocation [11,12], though paired oral samples would be required to confirm this directly.

### 3.3. Functional Metabolite Differences

Across all collapsed functional metabolites tested, predicted butyrate production showed the largest effect size difference between responders and non-responders at T0 (Figure 2A). However, this difference was not statistically significant (*p* = 0.179, Wilcoxon rank-sum; Figure 2B), and no collapsed metabolite reached nominal significance. The ranking of butyrate as the top feature by effect size and random forest importance should therefore be interpreted as a prioritisation signal for future hypothesis testing, not as evidence of a validated association. The full set of collapsed features is reported in Table S6.

### 3.4. Imputed Metabolites

Individual imputed metabolite analysis provided additional granularity (Figure 3A). Among the hundreds of metabolites tested, those reaching nominal significance and classified as being of established microbial origin fell into two main groups.

SCFAs and fermentation products. Butyrate, propionate, and hydroxyisovaleric acid (a branched-chain fatty acid derived from microbial fermentation of leucine) were nominally elevated in responders. The co-elevation of these three fermentation products is consistent with greater overall saccharolytic and amino acid fermentation capacity in the responder microbiome, although the small sample size precludes firm conclusions.

Bile acids. Responders showed elevated levels of both conjugated (taurochenodesoxycholic acid) and unconjugated (chenodeoxycholate, cholate) primary bile acids. This global elevation suggests greater overall bile acid secretory and microbial processing flux, consistent with a more metabolically active commensal community, including bile salt hydrolase (BSH) activity by commensals such as *Lactobacillaceae*, *Bifidobacterium*, *Clostridium*, and *Bacteroides* [31,32]. This interpretation is supported by the enrichment of *Collinsella aerofaciens* among responders. However, bile acid levels in PDAC are also influenced by the degree of biliary obstruction [33], which was not systematically quantified in this cohort (biliary stenting data were not available), and this represents an uncontrolled confounder.

The overall pattern (Figure 3A) suggests that the discriminant signal was concentrated in SCFAs/fermentation products and bile acids, while lipid and host-derived metabolites contributed minimally. However, given the multiple comparisons performed and the small sample size, these patterns should be regarded as hypothesis-generating.

### 3.5. Multivariable Modelling

Disease stage at diagnosis showed a non-significant univariable association with treatment response (stage IV vs. III: OR = 0.20, *p* = 0.22; Table 1). Multivariable logistic regression including both predicted butyrate and stage showed a lower AIC (20.6) than either predictor alone (butyrate: 21.9; stage: 21.4), with a pseudo-R^2^ of 0.24 (Table 1; full model outputs in Table S7). In the multivariable model, neither predictor reached significance (butyrate OR = 5.32, 95% CI 0.77–80.9, *p* = 0.14; stage IV OR = 0.058, 95% CI 0.0004–1.20, *p* = 0.14). The extremely wide confidence intervals reflect the sample size constraint and the instability inherent in modelling 6 events with 2 predictors. The ROC AUC of the multivariable model was 0.78 (95% CI 0.52–1.00; sensitivity 0.67, specificity 0.88; Supplementary Figure S2); it is reported for completeness, but should not be interpreted as validated predictive performance. These modelling results are exploratory and serve primarily to motivate future adequately powered studies.

## 4. Discussion

This exploratory study describes compositional and functional microbiome features that differ between PDAC chemotherapy responders and non-responders at diagnosis. While several biologically coherent signals were observed, important confounders limit the interpretability of these associations, and the findings should be regarded as hypothesis-generating rather than as evidence of predictive biomarkers.

### 4.1. Compositional Signals and Their Limitations

The enrichment of *Collinsella aerofaciens* in responders was the most technically robust taxonomic signal, with high prevalence (100%), substantial abundance difference (17.2% vs. 0.5%), and established biological relevance in bile acid metabolism and SCFA cross-feeding [29]. *Butyrivibrio fibrisolvens*, although showing the lowest nominal *p*-value, was present at very low abundance near the limit of metagenomic detection (median 0.0002%), with a prevalence of approximately 50%. At this abundance, species-level identification from short-read metagenomics is inherently uncertain, and the signal requires confirmation by targeted approaches (e.g., qPCR or long-read sequencing).

The enrichment of oral-origin species (*S. gordonii*, *Actinomyces* spp., *R. dentocariosa*) in non-responders is consistent with the growing literature on oro-intestinal translocation in PDAC [11,12,13]. In PDAC, multiple factors may facilitate translocation: biliary stent placement, duodenal obstruction, exocrine pancreatic insufficiency leading to altered gastric and small bowel pH, and systemic immunosuppression [13,34]. Oral taxa have also been detected within pancreatic tumour tissue, where they have been associated with immunosuppressive features of the tumour microenvironment [35], and *S. gordonii* in particular expresses surface adhesins that support polymicrobial biofilm formation [36]. However, without paired oral samples, we cannot confirm that these taxa originated from the oral cavity, and their faecal enrichment may alternatively reflect greater mucosal compromise associated with more advanced disease.

### 4.2. The Confounding Structure: Stage, Regimen, and Outcome

The most important limitation of this study is the non-random distribution of chemotherapy regimen between groups: mFOLFIRINOX was administered to 5/6 responders but only 2/9 non-responders (Fisher *p* = 0.041). Since mFOLFIRINOX has superior response rates compared to gemcitabine-based regimens in PDAC [3,4], the observed microbiome differences may reflect factors that influenced regimen selection (primarily disease stage and performance status) rather than a direct microbiome–response relationship. Stage III patients were more likely to receive mFOLFIRINOX and more likely to achieve resection, creating a stage–regimen–outcome chain that cannot be disentangled with *n* = 15.

While PERMANOVA showed that T0 microbiome composition did not differ significantly by subsequently assigned regimen (R^2^ = 0.08, *p* = 0.51), this negative result should be interpreted cautiously given the limited statistical power. The functional differences between groups may be mediated by disease severity rather than by the microbiome’s intrinsic capacity to modulate treatment response. Future studies should either control for regimen prospectively (e.g., restricting to a single regimen) or include sufficient sample sizes for regimen-stratified analyses.

### 4.3. Functional Predictions: Context and Caveats

The functional metabolite analysis relies on metagenomically predicted metabolic potential, not direct metabolite measurements. An estimated 90–95% of butyrate produced in the colon is absorbed by colonocytes, for which it is the primary energy source [37,38], making faecal SCFA concentrations a poor proxy for microbial production capacity [39]. Metagenomic prediction measures genetic capacity independently of host absorption, which may be advantageous in PDAC where biliary obstruction and altered motility further disconnect production from faecal concentrations. However, the predicted butyrate difference between groups did not reach significance (*p* = 0.179), and the direction and magnitude of the butyrate signal varied depending on the specific definition used (single enzyme abundance, butyrate-producing guild composition, or pathway-level score), indicating that the signal is not robust to analytical choices. This instability should temper any strong interpretation of butyrate as a mechanistic mediator in this cohort.

The bile acid elevation in responders is biologically plausible given the role of microbial BSH activity [31,32] and the immunomodulatory signalling of unconjugated bile acids through FXR and TGR5 [40], but bile acid levels in PDAC are heavily influenced by the degree of biliary obstruction, stent placement, and exocrine insufficiency [33], none of which were systematically recorded. The observed differences may therefore reflect disease-related variation in bile acid handling rather than microbiome-driven metabolic competence.

### 4.4. Strengths and Limitations

Strengths of this study include the prospective design, pre-treatment sampling eliminating the confounding effect of chemotherapy on the microbiome, whole-genome shotgun sequencing providing species-level resolution, and the multi-layered analytical approach (taxonomy, collapsed functional outputs, imputed metabolites). The principal limitations are the small sample size (*n* = 15), the confounded association between regimen, stage, and outcome, the absence of an independent validation cohort, the use of a proprietary functional database (though the EC-to-metabolite mapping is provided for scrutiny), the lack of direct metabolomic validation, and the absence of biliary stenting and drainage data. No a priori power calculation was performed, as the effect size of gut microbiome features on chemotherapy response in PDAC was unknown at study inception. The single-centre design further limits generalisability. Baseline demographic, clinical, nutritional and lifestyle characteristics, including inflammatory (neutrophil-to-lymphocyte ratio, C-reactive protein) and nutritional (albumin, prognostic nutritional index, sarcopenia, malnutrition risk) indices, Mediterranean-diet adherence, and antibiotic, proton pump inhibitor, supplement/probiotic and concomitant-medication use in the 30 days preceding sampling did not differ significantly between responders and non-responders (Supplementary Table S9), although the small sample size limits the power to exclude residual confounding. The observational nature of this study precludes causal inference, and all findings should be interpreted as hypothesis-generating.

## 5. Conclusions

This exploratory study identifies compositional and functional gut microbiome features that differ between PDAC chemotherapy responders and non-responders at diagnosis, including enrichment of *Collinsella aerofaciens* and predicted butyrate-producing capacity in responders, and enrichment of oral-origin taxa in non-responders. These associations are confounded by the non-random distribution of disease stage and chemotherapy regimen between groups. Validation in larger, regimen-stratified cohorts with concurrent faecal metabolomics, biliary obstruction assessment, and paired oral sampling is required before any predictive or mechanistic claims can be made. If confirmed independently of confounders, these findings could motivate the investigation of microbiome-directed adjuncts to chemotherapy in PDAC.

## Figures and Tables

**Figure 1:**
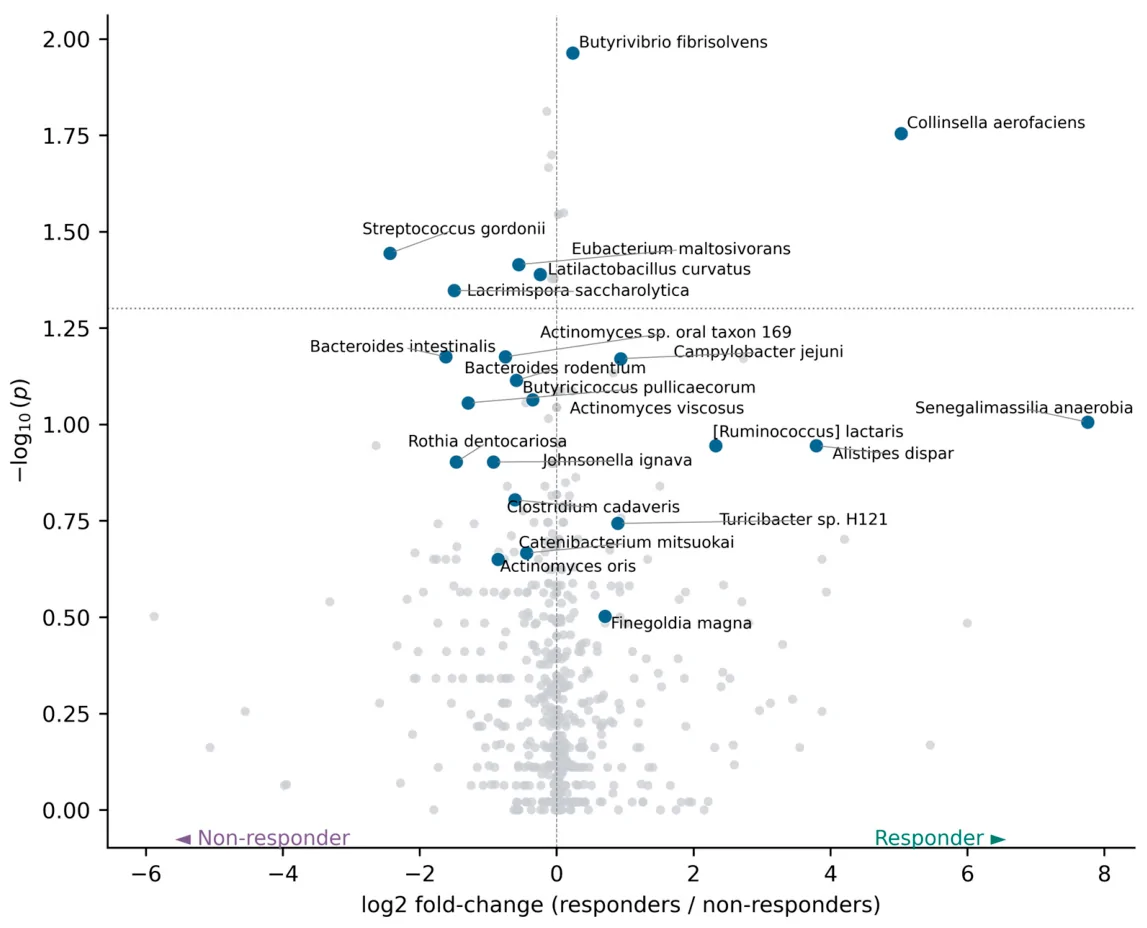
Differential gut microbial composition at baseline (T0) in chemotherapy responders versus non-responders. Volcano plot of species-level differential relative abundance (Wilcoxon rank-sum test): the x-axis shows the log2 fold-change (responders vs. non-responders) and the y-axis the −log10(*p*-value). Species enriched in responders (e.g., *Collinsella aerofaciens*, *Butyrivibrio fibrisolvens*) lie on the right; oral-origin taxa enriched in non-responders (*Streptococcus gordonii*, *Rothia dentocariosa*, *Actinomyces* spp.) lie on the left. Coloured (blue) points indicate nominal *p* < 0.05; no species remained significant after Benjamini–Hochberg correction. Selected species are labelled. Group sizes: *n* = 6 responders and *n* = 9 non-responders.

**Figure 2:**
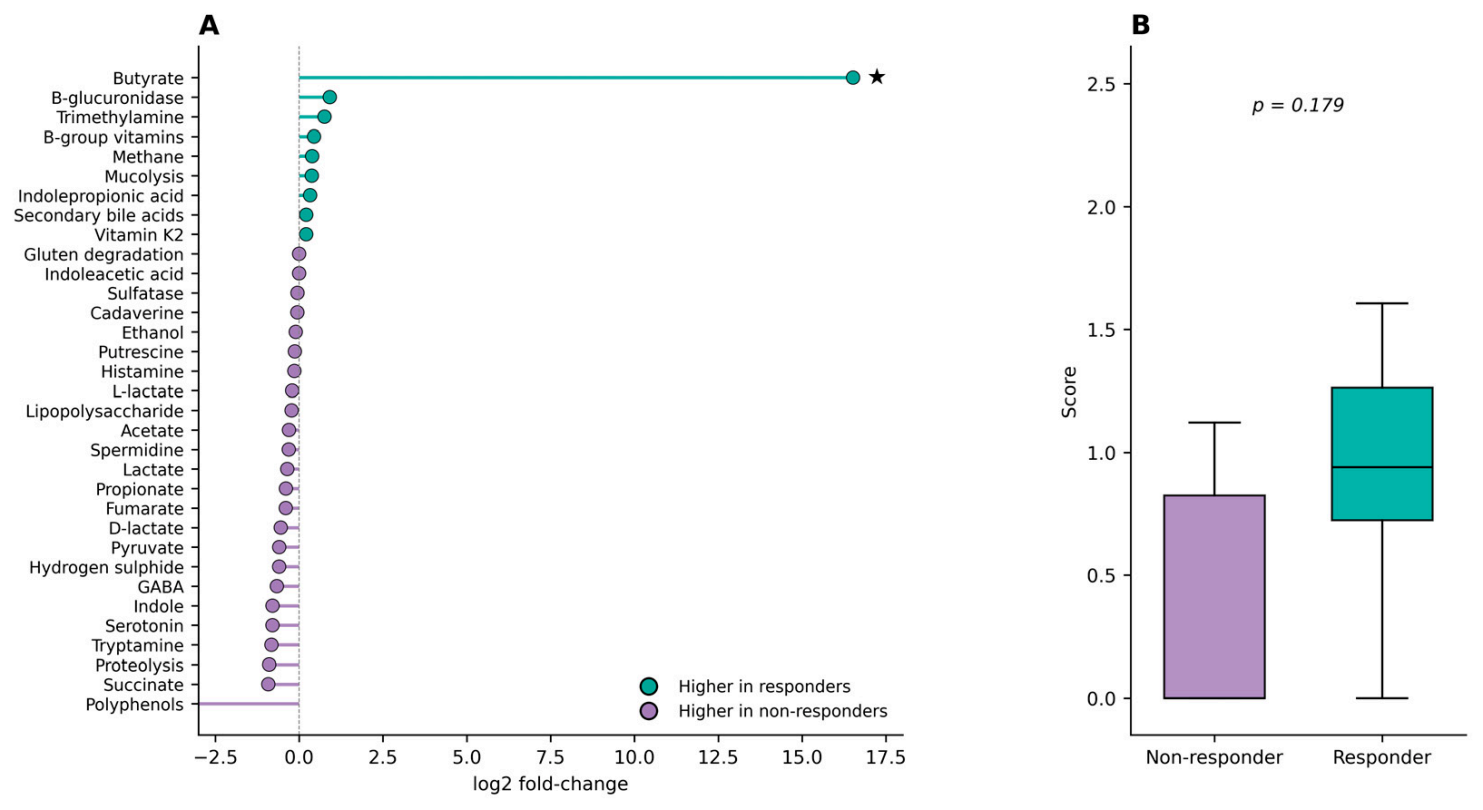
Predicted microbial metabolic potential at baseline in responders and non-responders. (**A**) Forest plot showing log2 fold-change (responders/non-responders) for all collapsed functional metabolites at T0, ordered by absolute effect size. The star (★) marks predicted butyrate as the top-ranked feature by effect size and random forest importance; it denotes ranking, not univariate statistical significance. None of the collapsed features reached nominal significance (all *p* > 0.05; exact *p*-values in Table S6). (**B**) Boxplot of predicted butyrate production score by group, higher in responders (*p* = 0.179, Wilcoxon rank-sum test). Functional potentials were inferred from shotgun metagenomes using the assembly-based enzyme-to-metabolite mapping (Tables S1 and S2). Group sizes: *n* = 6 responders and *n* = 9 non-responders.

**Figure 3:**
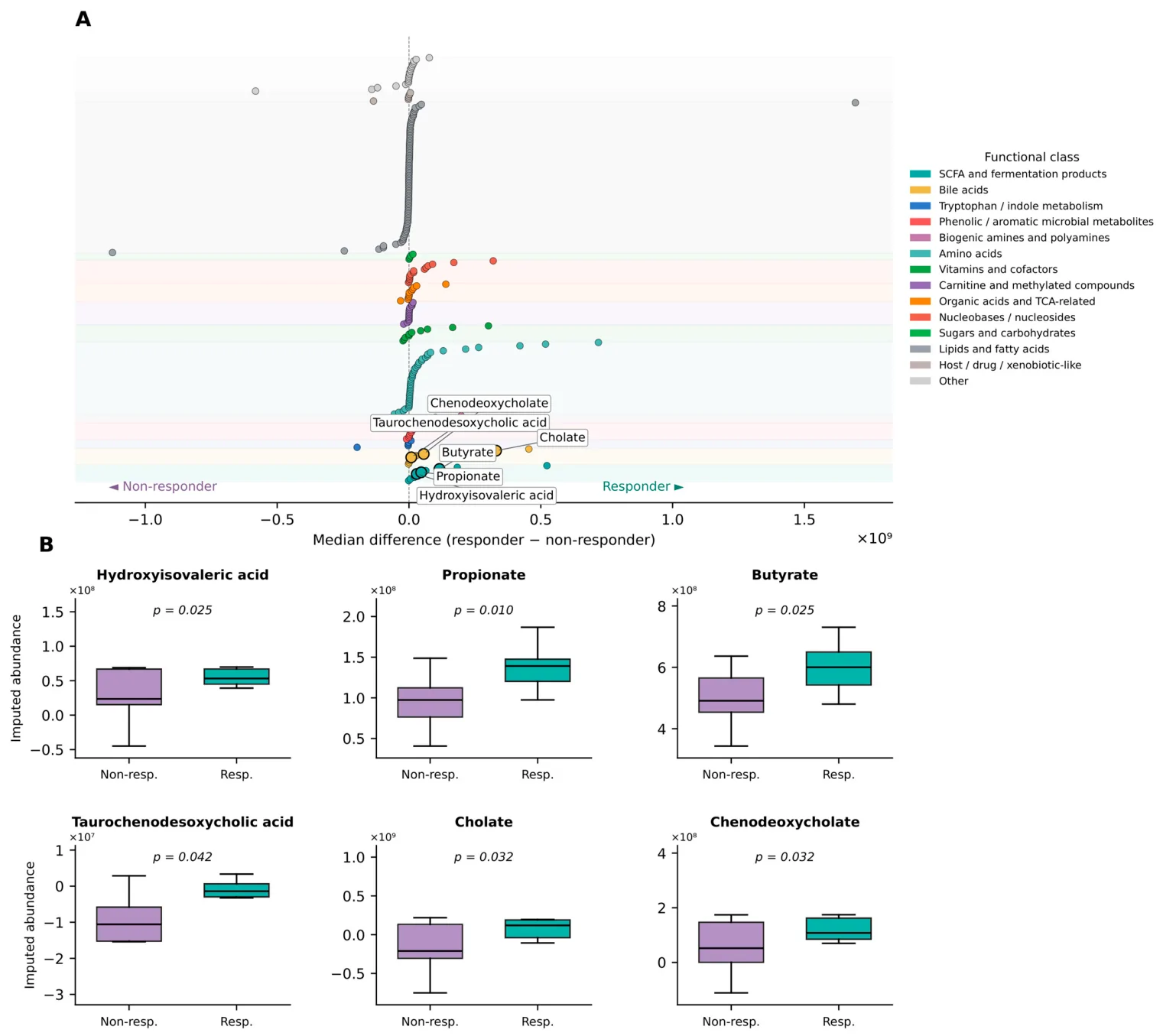
Imputed metabolites at T0 in responders and non-responders. (**A**) Overview of all tested imputed metabolites, ordered by functional class and within-class ranking score; the x-axis shows the median difference (responders–non-responders) and labelled metabolites are those shown in panel (**B**). (**B**) Imputed abundance of selected nominally discriminant metabolites (butyrate, propionate, hydroxyisovaleric acid, cholate, chenodeoxycholate, taurochenodesoxycholic acid). Full results are reported in Table S5. Group sizes: *n* = 6 responders, and *n* = 9 non-responders.

**Table 1:** Logistic regression models for chemotherapy response (exploratory analysis).

Model	Predictor	OR	95% CI	*p*	AIC ^a^	Pseudo-R^2^
Multivariable	Predicted butyrate	5.32	0.77–80.9	0.14	20.6	0.24
	Stage IV vs. III	0.058	0.0004–1.20	0.14		
Univariable	Predicted butyrate	2.43	-	0.29	21.9	-
Univariable	Stage IV vs. III	0.20	-	0.22	21.4	-

^a^ AIC and pseudo-R^2^ are model-level statistics and are reported once per model. OR, odds ratio; CI, confidence interval; AIC, Akaike information criterion; -, not applicable. Full model outputs are reported in Table S7.

## Data Availability

Raw metagenomic sequences have been deposited in the NCBI Sequence Read Archive under BioProject accession PRJNA1481895 (data held private until publication; reviewer access link available upon request to the corresponding authors). Processed taxonomic abundance tables, EC count matrices, and the EC-to-metabolite mapping are provided as Supplementary Materials.

## Supplementary Materials

The following supporting information can be downloaded at: https://www.mdpi.com/article/10.3390/metabo16100720/s1. Table S1: Collapsed functional metabolite categories; Table S2: Enzyme Commission (EC) number-to-metabolite mapping; Table S3: Baseline clinical and demographic characteristics of the 15 enrolled patients; Table S4: Complete differential-abundance results for all species (responders vs. non-responders at T0); Table S5: Complete differential-abundance results for all imputed metabolites, annotated by functional class; Table S6: Differential abundance of collapsed functional metabolite categories between response groups at T0; Table S7: Logistic regression model outputs for chemotherapy-response prediction. Table S8: PERMANOVA of baseline (T0) faecal species composition by subsequently assigned chemotherapy regimen; Table S9: Baseline demographic, clinical, nutritional and lifestyle characteristics of responders versus non-responders; Figure S1: study workflow; Figure S2: ROC curve for the multivariable model.
