# Supplementary material for: Gut Microbiome Compositional and Functional Signatures Associated with Chemotherapy Response in Pancreatic Ductal Adenocarcinoma: An Exploratory Cohort Study
Source: Metabolites. 2026 Sep 28;16(10):720. doi: 10.3390/metabo16100720 (PMC13707113; doi:10.3390/metabo16100720)

Supplementary Materials

Gut Microbial Butyrate-Producing Potential and Metabolic Competence at Diagnosis Predict Chemotherapy Response in Pancreatic Ductal Adenocarcinoma

In all data tables, group g1 = responders and group g2 = non-responders; a positive log2 fold-change indicates enrichment in responders. p, nominal p value; padj, Benjamini-Hochberg adjusted p value.

Table S1. Collapsed functional metabolite categories: definition of the aggregated functional categories used to summarise microbial metabolic potential, with representative pathways and enzymes.

Category	Constituent pathways	Representative enzymes	Biological interpretation
GABA	Glutamate decarboxylation	gadA, gadB	Neuroactive microbial metabolite linked to gut-brain signalling.
B glucuronidase	Deconjugation of glucuronides	uidA / beta-glucuronidase	Microbial deconjugation potential for host/drug metabolites.
Sulfatase	Sulfate ester hydrolysis	sulfatases	Broad deconjugation / mucin-related functional signal.

Table S2. Enzyme Commission (EC) number-to-metabolite mapping used by the functional inference framework. Because the table is a wide matrix (26 metabolites x constituent EC numbers), it is provided as a separate machine-readable file (Supplementary_Table_S2.tsv); it enables independent verification of the inference logic (which enzymatic activities map to which predicted metabolites).

Predicted metabolite	Enzyme Commission (EC) numbers
Acetate	1.13.11.50, 1.13.12.4, 1.2.5.1, 2.1.1.152, 2.3.1.187, 2.5.1.134, 2.5.1.144, 2.5.1.47, 2.5.1.49, 2.6.99.3, 2.8.3.1, 2.8.3.10, 2.8.3.12, 2.8.3.19, 3.1.1.106, 3.1.1.112, 3.1.1.113, 3.1.1.114, 3.1.1.2, 3.1.1.41, 3.1.1.47, 3.1.1.53, 3.1.1.6, 3.1.1.7, 3.1.2.1, 3.11.1.2, 3.5.1.103, 3.5.1.104, 3.5.1.105, 3.5.1.108, 3.5.1.112, 3.5.1.113, 3.5.1.114, 3.5.1.125, 3.5.1.135, 3.5.1.136, 3.5.1.14, 3.5.1.16, 3.5.1.25, 3.5.1.29, 3.5.1.41, 3.5.1.47, 3.5.1.48, 3.5.1.62, 3.5.1.76, 3.7.1.24, 3.7.1.25, 4.1.3.26, 4.1.3.6
Butyrate	2.8.3.9, 3.7.1.7, 5.4.99.13, 2.3.1.19, 6.2.1.2, 1.3.1.86, 1.3.1.44
Propionate	1.2.1.27, 1.2.1.87, 2.1.3.1, 2.8.3.1, 2.8.3.27, 3.13.1.4, 4.1.3.24, 4.1.3.32, 6.2.1.17, 7.2.4.3
Succinate	1.14.11.1, 1.14.11.16, 1.14.11.17, 1.14.11.18, 1.14.11.2, 1.14.11.21, 1.14.11.26, 1.14.11.28, 1.14.11.30, 1.14.11.33, 1.14.11.35, 1.14.11.36, 1.14.11.37, 1.14.11.39, 1.14.11.40, 1.14.11.41, 1.14.11.43, 1.14.11.44, 1.14.11.45, 1.14.11.46, 1.14.11.47, 1.14.11.49, 1.14.11.52, 1.14.11.55, 1.14.11.56, 1.14.11.57, 1.14.11.58, 1.14.11.64, 1.14.11.70, 1.14.11.71, 1.14.11.72, 1.14.11.73, 1.14.11.74, 1.14.11.75, 1.14.11.77, 1.14.11.78, 1.14.11.8, 1.14.20.1, 1.14.20.10, 1.14.20.11, 1.14.20.15, 1.14.20.3, 1.14.20.7, 1.14.20.9, 1.2.1.16, 1.2.1.24, 1.2.1.79, 1.3.4.1, 1.3.98.1
Lactate	1.1.1.401, 1.2.1.22, 2.3.3.9, 3.1.2.30, 3.5.1.124, 3.7.1.26, 4.1.1.101, 4.1.2.17, 4.1.2.19, 4.1.2.53, 4.1.2.64
GABA	1.2.1.19, 1.5.3.19, 3.5.1.94, 3.5.3.7, 4.1.1.15
Serotonin	2.4.2.18, 4.1.1.28, 4.1.1.48, 4.1.3.27, 4.2.1.20, 5.3.1.24
Histamine	1.1.1.23, 4.1.1.22
Indole	4.1.99.1, 4.2.1.20
Indoleacetic acid	1.14.13.168, 3.5.1.134
Indolepropionic acid	1.2.1.3, 1.2.1.4, 1.2.1.5, 1.2.5.2, 1.2.99.7
Tryptamine	4.1.1.105, 4.2.1.20
Trimethylamine	1.14.13.239, 3.1.1.7, 3.1.3.75, 3.1.4.4, 3.1.4.41, 3.1.6.6, 4.3.99.4
Putrescine	1.5.1.43, 1.5.3.17, 3.5.1.53, 3.5.1.62, 3.5.3.11, 4.1.1.116, 4.1.1.17, 7.6.2.16
Cadaverine	4.1.1.116, 4.1.1.18
Spermidine	1.5.3.17, 2.5.1.16, 3.5.1.48, 3.5.1.78, 3.5.3.24, 4.1.1.96
B vitamins	1.16.1.6, 1.5.1.30, 1.5.1.41, 2.4.2.21, 2.4.2.55, 2.5.1.9, 2.8.1.6, 3.1.3.100, 3.1.3.102, 3.5.1.19, 6.3.2.1, 7.6.2.15, 7.6.2.8, 2.1.1.394
Vitamin K2	1.12.5.1, 1.8.5.3, 7.1.1.12, 7.1.1.5
Mucolysis	3.1.21.1, 3.2.1.18, 3.2.1.23, 3.2.1.24, 3.2.1.25, 3.2.1.35, 3.2.1.51, 3.4.21.4, 3.4.22.1, 3.4.22.15, 3.4.22.38, 3.4.24.3, 4.1.3.3, 4.2.2.1
Secondary bile acids	1.1.1.145, 1.1.1.159, 1.1.1.176, 1.1.1.357, 1.1.1.391, 1.14.15.15, 1.3.1.22, 1.3.1.31, 3.5.1.24
Lipopolysaccharide	2.3.1.129, 2.4.1.182, 2.4.99.12, 2.4.99.13, 2.4.99.14, 2.4.99.15, 2.4.99.23, 2.4.99.24, 2.4.99.25, 2.4.99.27, 2.7.1.235, 3.2.1.144, 3.5.1.108, 3.6.1.54, 7.5.2.5
Ethanol	1.1.1.1, 3.1.1.113
Hydrogen sulfide	1.12.98.4, 1.13.11.55, 1.3.99.36, 1.8.3.4, 1.8.3.7, 1.8.98.7, 2.1.1.251, 2.8.1.2, 3.13.1.5, 3.13.1.7, 4.4.1.2
Methane	1.3.4.1, 1.8.7.3, 1.8.98.1, 1.8.98.4, 1.8.98.5, 1.8.98.6, 2.1.1.246, 2.1.1.247, 2.1.1.251, 2.1.1.377, 2.8.4.1, 4.4.1.23, 4.7.1.1
Fumarate	1.3.1.6, 1.3.2.4, 1.3.5.1, 3.7.1.14, 3.7.1.2, 3.7.1.20, 4.1.99.11, 4.2.1.2, 4.3.1.1, 4.3.2.1, 4.3.2.2, 4.3.99.5, 5.2.1.1, 7.1.1.12
Pyruvate	1.1.1.27, 1.1.1.28, 1.1.1.38, 1.1.1.39, 1.1.1.40, 1.1.1.436, 1.1.1.83, 1.1.2.3, 1.1.2.4, 1.1.3.2, 1.1.5.12, 1.1.99.6, 1.14.11.43, 1.14.11.44, 1.4.1.1, 1.4.1.17, 1.5.1.11, 1.5.1.16, 1.5.1.24, 1.5.1.28, 1.5.1.52, 2.3.1.54, 2.6.1.108, 2.6.1.18, 2.6.1.2, 2.6.1.21, 2.6.1.44, 2.7.1.121, 2.7.3.9, 2.8.1.2, 3.1.3.60, 3.11.1.3, 3.3.2.1, 3.3.2.13, 3.3.2.15, 3.5.99.10, 3.7.1.20, 3.7.1.23, 3.7.1.26, 3.7.1.5, 4.1.1.112, 4.1.2.14, 4.1.2.20, 4.1.2.21, 4.1.2.28, 4.1.2.34, 4.1.2.45, 4.1.2.51, 4.1.2.52

Note: each column of the original matrix (one per metabolite) has been transposed to a metabolite -> EC-number list for readability; content is unchanged.

Table S3. Baseline clinical and demographic characteristics of the 15 enrolled patients (sex: M/F; Stage III/IV; PNI, prognostic nutritional index; CA19-9 and CEA in serum; NLR, neutrophil-to-lymphocyte ratio).

SampleID (T0)	Response	Stage	Age	Sex	BMI	PNI	Albumin	CA19-9	CEA	NLR
G10723	Non-responder	4	67	F	27.9	49.6	38.1	22233	44.5	3.09
G13023	Non-responder	4	63	M	26.8	49.1	42.1	15714	7.2	3.77
G19907	Non-responder	3	76	M	24.8	48.6	42.1	71	1.3	3.00
G26872	Non-responder	4	75	M	35.3	39.2	34.2	6056	29.0	7.40
G47339	Non-responder	4	64	M	28.1	45.2	41.7	597	1.8	4.29
G61404	Non-responder	3	57	F	21.5	56.1	49.1	690	2.7	3.86
G72849	Non-responder	3	70	M	24.2	51.0	43.5	5449	32.7	3.27
G96580	Non-responder	3	57	F	20.3	49.0	44.5	278	1.7	1.89
ZZ12	Non-responder	4	61	M	26.0	41.8	35.6	1724	4.4	7.00
G04535	Responder	3	75	M	23.7	50.0	45.5	159	2.2	4.22
G20669	Responder	3	55	M	25.4	49.8	40.8	52.7	0.9	2.83
G31691	Responder	3	62	F	24.7	50.2	36.7	138	2.0	1.59
G32118	Responder	3	70	M	26.4	56.9	38.9	1690	14.1	0.56
G51485	Responder	4	68	M	26.0	40.8	37.8	1818	7.0	5.63
G99746	Responder	3	66	F	23.2	53.0	44.0	146	1.4	3.06

Table S4. Complete differential-abundance results for all species (responders vs non-responders at T0; Wilcoxon rank-sum test, log2 fold-change, Benjamini-Hochberg FDR). The full table (3,082 species) is provided as a separate machine-readable file (Supplementary_Table_S4.tsv).

Species	Median g1 (resp.)	Median g2 (non-resp.)	log2FC	p	padj
Lachnoclostridium phytofermentans	7.430370645610722e-7	0	0.8016032477710648	0.007419996550473036	0.7335983792666142
Aminipila luticellarii	0	6.861890369541366e-7	-0.7537662844344282	0.010559848267034103	0.7335983792666142
Butyrivibrio fibrisolvens	1.7763961533133109e-6	0	1.473213435223093	0.010725991632709752	0.7335983792666142
Vaginimicrobium propionicum	1.8540736501092327e-7	0	0.2453829262015322	0.011641875871611616	0.7335983792666142
Streptococcus gordonii	7.819864127891818e-6	1.9893176608035183e-4	-4.502607471212507	0.018207282913165267	0.7335983792666142
Bacteroides intestinalis	1.4252902123503788e-6	2.9581730564177784e-5	-3.6564407515875983	0.021483027887915108	0.7335983792666142
Bacteroides rodentium	9.18324665133105e-6	2.8478701834712553e-5	-1.533475394455701	0.02451461446095003	0.7335983792666142
Actinomyces sp oral taxon 169	3.7081473002184654e-7	7.944535410013714e-6	-2.7059729494368963	0.027416676231284604	0.7335983792666142
Actinomyces viscosus	8.493593282586945e-7	5.691918019782585e-6	-1.8553942056978185	0.027416676231284604	0.7335983792666142
Eubacterium maltosivorans	5.358475937865802e-7	4.130901770210565e-6	-1.7401773450474605	0.027416676231284604	0.7335983792666142
Fusobacterium pseudoperiodonticum	0	3.916932080926763e-7	-0.47684121122616707	0.028973416279444515	0.7335983792666142
Oxalobacter paraformigenes	0	3.430945184770683e-7	-0.42556083606833583	0.028973416279444515	0.7335983792666142
Megasphaera elsdenii	0	2.9156075201121485e-7	-0.3691155060683209	0.028973416279444515	0.7335983792666142
Lacrimispora saccharolytica	1.3920208866924462e-6	1.785628375042043e-5	-2.9787434815653033	0.03114151291160722	0.7335983792666142
Latilactobacillus curvatus	0	1.7682711727260691e-6	-1.4689852725935209	0.03145522423747072	0.7335983792666142
Leuconostoc carnosum	0	8.841355863630346e-7	-0.9139027880585241	0.03145522423747072	0.7335983792666142
Collinsella aerofaciens	0.1674220217022408	0.029827390761952406	2.488739869367092	0.03199741435035553	0.7335983792666142
Campylobacter jejuni	1.085111185370553e-5	8.76970899266354e-7	2.6585442292033328	0.03471381477652668	0.7335983792666142
Corynebacterium simulans	2.527278177003487e-7	0	0.3250729919067554	0.03963449389327917	0.7335983792666142
Alistipes dispar	2.477521470368512e-4	9.096998015117321e-6	4.622710650393435	0.041478129713423836	0.7335983792666142
Blautia massiliensis (ex Durand et al 2017)	0	0	0	0.04858293092335843	0.7335983792666142
Gardnerella swidsinskii	0	0	0	0.04858293092335843	0.7335983792666142
Acidipropionibacterium acidipropionici	0	0	0	0.04858293092335843	0.7335983792666142
Anaeromyxobacter oryzae	0	0	0	0.04858293092335843	0.7335983792666142
Ascaris lumbricoides	0	0	0	0.04858293092335843	0.7335983792666142
Ligilactobacillus apodemi	0	0	0	0.04858293092335843	0.7335983792666142
Thermoclostridium stercorarium	0	0	0	0.04858293092335843	0.7335983792666142
Anaeromicropila herbilytica	0	0	0	0.04858293092335843	0.7335983792666142
Mycolicibacterium fortuitum	0	0	0	0.04858293092335843	0.7335983792666142
Afonbuvirus coli	0	0	0	0.04858293092335843	0.7335983792666142
Shigella flexneri	0	0	0	0.04858293092335843	0.7335983792666142
Anaerococcus mediterraneensis	0	0	0	0.04858293092335843	0.7335983792666142

Species	Median g1 (resp.)	Median g2 (non-resp.)	log2FC	p	padj
Kluyvera sp CRP	0	0	0	0.04858293092335843	0.7335983792666142
Novosphingobium sp EMRT-2	0	0	0	0.04858293092335843	0.7335983792666142
Anaerococcus sp Marseille-Q7828	0	0	0	0.04858293092335843	0.7335983792666142
Leclercia adecarboxylata	0	0	0	0.04858293092335843	0.7335983792666142
Pseudoclostridium thermosuccinogenes	0	0	0	0.04858293092335843	0.7335983792666142
Selenomonas noxia	0	2.385899145180054e-7	-0.3086986037644786	0.05090760866384896	0.7335983792666142
Fretibacterium fastidiosum	0	1.6104584459849018e-7	-0.21542493901578902	0.05090760866384896	0.7335983792666142
Kurthia sp YJT4	2.41075389095678e-7	0	0.3115907546068575	0.0537380668130376	0.7335983792666142
Rothia dentocariosa	2.6145645487243623e-6	4.1197005024836794e-5	-3.545246846408599	0.06091128006791017	0.7335983792666142
Johnsonella ignava	1.4242188448575527e-6	1.2881128214357631e-5	-2.517532982182771	0.06091128006791017	0.7335983792666142
Clostridium cadaveris	5.460210504864351e-6	1.3659541516475811e-5	-1.182186902492521	0.06091128006791017	0.7335983792666142
[Ruminococcus] lactaris	8.14152042289976e-4	1.488333119734907e-4	2.44371076680154	0.06679594914889034	0.7335983792666142
Actinomyces oris	5.130784203236518e-6	2.2240984620701416e-5	-1.9225276614326106	0.06679594914889034	0.7335983792666142
Butyricicoccus pullicaecorum	1.3884061528880662e-5	4.7973214887571776e-5	-1.718224646563025	0.06679594914889034	0.7335983792666142
Eggerthella guodeyinii	1.4885316656258225e-5	2.793948780850163e-6	2.065921741581152	0.06723146114649427	0.7335983792666142
Catenibacterium mitsuokai	1.9985176843934483e-6	1.294302136118793e-5	-2.217221831085251	0.067656862082081	0.7335983792666142
Afonbuvirus intestinihominis	1.8540736501092327e-7	0	0.2453829262015322	0.07180604730018461	0.7335983792666142
Senegalimassilia anaerobia	0.004970071188589274	1.953755343930469e-4	4.661869657032199	0.07500529281377794	0.7335983792666142
Romboutsia hominis	7.465310244963753e-7	0	0.8044922702675772	0.07879323127885379	0.7335983792666142
Segatella bryantii	5.358475937865802e-7	0	0.6190350607222442	0.08047174179102921	0.7335983792666142
Selenomonas artemidis	0	4.866730997870243e-7	-0.5720874522567717	0.08047174179102921	0.7335983792666142
Caproicibacterium sp BJN0003	0	3.916932080926763e-7	-0.47684121122616707	0.08047174179102921	0.7335983792666142
Prevotella pallens	0	0	0	0.0856692277660039	0.7335983792666142
Eikenella corrodens	0	0	0	0.0856692277660039	0.7335983792666142
Bifidobacterium scardovii	0	0	0	0.0856692277660039	0.7335983792666142
Companilactobacillus paralimentarius	0	0	0	0.0856692277660039	0.7335983792666142
Fusobacterium periodonticum	0	0	0	0.0856692277660039	0.7335983792666142
Actinomyces sp Chiba101	0	0	0	0.0856692277660039	0.7335983792666142
Neisseria sp oral taxon 014	0	0	0	0.0856692277660039	0.7335983792666142
Megasphaera hexanoica	0	0	0	0.0856692277660039	0.7335983792666142
Bacteroides heparinolyticus	0	0	0	0.0856692277660039	0.7335983792666142
Raoultella planticola	0	0	0	0.0856692277660039	0.7335983792666142
Kahnovirus copri	2.41075389095678e-6	0	1.7700906584676266	0.09457368795259105	0.7335983792666142
Proteus mirabilis	9.270368250546164e-8	0	0.1279022265007203	0.09457368795259105	0.7335983792666142

Species	Median g1 (resp.)	Median g2 (non-resp.)	log2FC	p	padj
Turicibacter sp H121	8.598696764962284e-6	0	3.2628385414215444	0.0957044355798869	0.7335983792666142
Finegoldia magna	1.669454275340827e-5	8.841355863630346e-7	3.2313297882351835	0.09944535603344483	0.7335983792666142
Clostridium saudiense	0.0037816150050266045	1.4448660980792074e-5	7.93575833862608	0.10050328269212787	0.7335983792666142
Parascardovia denticolens	2.377403656382007e-6	1.3400490542864201e-5	-2.0921314359080747	0.10050328269212787	0.7335983792666142
Actinomyces graevenitzii	3.268816898515527e-5	2.631542974729725e-4	-2.9710669584600433	0.10245636716224953	0.7335983792666142
Eggerthella lenta	0.004154755860937903	5.802087767648645e-4	2.8379825082083543	0.10245636716224953	0.7335983792666142
Vescimonas coprocola	0.0011441289412070213	0.005115398956704504	-2.1596187097637825	0.10245636716224953	0.7335983792666142
Streptococcus thermophilus	3.293700007522054e-4	0.0013101357193378309	-1.988662436806882	0.10245636716224953	0.7335983792666142
Stomatobaculum longum	4.2051345218294607e-7	2.6650887259566748e-6	-1.3674356302959294	0.10245636716224953	0.7335983792666142
Roseburia sp 499	3.122452541588731e-6	7.887005474503576e-6	-1.1081944916939845	0.10245636716224953	0.7335983792666142
Campylobacter coli	0	6.863375228086388e-7	-0.7538933226073085	0.10586883619681647	0.7335983792666142
Alistipes indistinctus	9.336263419236244e-5	4.5807345193739726e-4	-2.2824374059030217	0.10949309673336423	0.7335983792666142
Sharpea azabuensis	4.432826256458744e-7	7.204431595995595e-6	-2.5070495490380122	0.12032691744735911	0.7335983792666142
Propionimicrobium lymphophilum	0	4.831375337954705e-7	-0.5686523874921522	0.12242537814561909	0.7335983792666142
Segatella maculosa	0	2.611783006799516e-7	-0.33477225292429297	0.12242537814561909	0.7335983792666142
Muribaculum intestinale	0	8.537213447797382e-7	-0.8904243913643604	0.12299739758412545	0.7335983792666142
Anaerostipes caccae	3.4377640864565666e-5	1.0280562941139172e-5	1.648998780771071	0.1246498599439776	0.7335983792666142
Carjivirus communis	4.2051345218294607e-7	0	0.5064124938334955	0.13206013443683942	0.7335983792666142
Fenollaria massiliensis	4.153660092483436e-7	0	0.5011751777546156	0.13206013443683942	0.7335983792666142
Schaalia meyeri	0	1.26660615716696e-6	-1.1805337318534166	0.13476661922794178	0.7335983792666142
Fusobacterium necrophorum	5.054556354006974e-7	0	0.5902001935953183	0.1384369323487725	0.7335983792666142
Brachyspira pilosicoli	4.599172884748407e-7	0	0.5458866356208033	0.1384369323487725	0.7335983792666142
Facklamia hominis	1.9055480794552572e-7	0	0.2516340375180648	0.1384369323487725	0.7335983792666142
Anaerotignum sp MB30-C6	0	0	0	0.1391789362347769	0.7335983792666142
Streptococcus sp oral taxon 064	0	0	0	0.1391789362347769	0.7335983792666142
Anaerococcus vaginalis	0	0	0	0.1391789362347769	0.7335983792666142
Porphyromonas gulae	0	0	0	0.1391789362347769	0.7335983792666142
Pantoea agglomerans	0	0	0	0.1391789362347769	0.7335983792666142
Limosilactobacillus vaginalis	0	0	0	0.1391789362347769	0.7335983792666142
Neisseria sp Marseille-Q5346	0	0	0	0.1391789362347769	0.7335983792666142
Streptococcus criceti	0	0	0	0.1391789362347769	0.7335983792666142
Streptococcus sp 116-D4	0	0	0	0.1391789362347769	0.7335983792666142
Actinomyces wuliandei	0	0	0	0.1391789362347769	0.7335983792666142
Caproiciproducens sp CPB-2	0	0	0	0.1391789362347769	0.7335983792666142

Species	Median g1 (resp.)	Median g2 (non-resp.)	log2FC	p	padj
Prevotella sp E13-17	0	0	0	0.1391789362347769	0.7335983792666142
Streptococcus phage SpSL1	0	0	0	0.1391789362347769	0.7335983792666142
Acidaminococcus fermentans	0	0	0	0.1391789362347769	0.7335983792666142
Neisseria cinerea	0	0	0	0.1391789362347769	0.7335983792666142
Streptococcus pseudoporcinus	0	0	0	0.1391789362347769	0.7335983792666142
Fusobacterium vincentii	0	0	0	0.1391789362347769	0.7335983792666142
Prevotella communis	0	0	0	0.1391789362347769	0.7335983792666142
Taranisvirus taranis	7.745001980099185e-6	0	3.128458710811188	0.14155098095598728	0.7335983792666142
Veillonella nakazawae	5.054556354006974e-7	1.1691395676507625e-5	-3.0755786327923893	0.14239632586251377	0.7335983792666142
Parabacteroides goldsteinii	2.8583221191828855e-6	1.2135539931301029e-5	-1.7674300035126145	0.14239632586251377	0.7335983792666142
Catonella morbi	1.543528159343835e-5	3.1107479488618615e-6	1.999323354345582	0.14574856099081243	0.7335983792666142
Rothia aeria	8.393418711190014e-7	4.733086404723247e-6	-1.6401223697727259	0.14574856099081243	0.7335983792666142
Phascolarctobacterium succinatutens	2.831197760862315e-7	1.7682711727260691e-6	-1.1093294238337919	0.14574856099081243	0.7335983792666142
Blautia liquoris	0	1.7214131777008846e-6	-1.4443560099382855	0.14682060431264085	0.7335983792666142
Prevotella denticola	1.3920208866924462e-6	0	1.258229986942395	0.14682060431264085	0.7335983792666142
Atopobium sp oral taxon 416	0	1.0443676632877843e-6	-1.0316546768541506	0.14682060431264085	0.7335983792666142
Treponema phagedenis	0	8.314509970359405e-7	-0.8729871002935282	0.1500109698827585	0.7335983792666142
Bacteroides zoogleoformans	0	2.611783006799516e-7	-0.33477225292429297	0.15039324859690031	0.7335983792666142
Veillonella rodentium	0	2.4808318526901893e-7	-0.3197140936357452	0.15039324859690031	0.7335983792666142
Veillonella atypica	5.368068088479088e-6	6.933655698896765e-5	-3.465347049399182	0.15050635638870935	0.7335983792666142
Gordonibacter pamelaeae	1.5579507583233926e-4	4.742413862031768e-5	1.6950819609791894	0.15050635638870935	0.7335983792666142
Oribacterium sinus	7.622192317821029e-7	7.231801439614965e-6	-2.2238147669381445	0.15244187790692604	0.7335983792666142
Enterocytozoon bieneusi	2.940037343995363e-6	8.877110927762119e-7	1.0615713212910411	0.15244187790692604	0.7335983792666142
Enterobacter roggenkampii	4.772439087005041e-6	0	2.5291810442073737	0.16511199483174352	0.7335983792666142
Jeotgalibaca arthritidis	1.5135887202359774e-6	0	1.3297486116453952	0.16511199483174352	0.7335983792666142
Candidatus Nanosynbacter featherlites	0	0	0	0.17083379767454282	0.7335983792666142
Atopobium minutum	0	0	0	0.17083379767454282	0.7335983792666142
Dehalobacterium formicoaceticum	0	0	0	0.17083379767454282	0.7335983792666142
Afonbuvirus faecalis	0	0	0	0.17083379767454282	0.7335983792666142
Corynebacterium xerosis	0	0	0	0.17083379767454282	0.7335983792666142
Lactobacillus ultunensis	0	0	0	0.17083379767454282	0.7335983792666142
Bacteroides sp M10	1.3563427710072052e-6	1.1159305821163227e-5	-2.3674395425767982	0.17221797231463226	0.7335983792666142
Schaalia cardiffensis	1.7149932715097314e-6	0	1.4409486226441592	0.17284911950681592	0.7335983792666142
Leuconostoc lactis	4.82150778191356e-7	0	0.5676922194953893	0.17284911950681592	0.7335983792666142

Species	Median g1 (resp.)	Median g2 (non-resp.)	log2FC	p	padj
Dialister invisus	1.1831434557618747e-6	7.602947043708843e-4	-8.445904298641981	0.17355758036503094	0.7335983792666142
Alloscardovia omnicolens	2.954834141402113e-6	1.6905410772943372e-4	-5.426232853211297	0.17422559473614996	0.7335983792666142
Bacteroides sp A1C1	3.4682748761768573e-6	2.1409000151790233e-5	-2.3262884355373963	0.1797026502908856	0.7335983792666142
Solobacterium moorei	5.494217973762416e-6	2.3166958619237824e-5	-1.8958082096334241	0.1797026502908856	0.7335983792666142
Actinomyces naeslundii	3.598407819811987e-5	1.1815611575216434e-4	-1.6878767773355472	0.1797026502908856	0.7335983792666142
Bacteroides faecis	1.5633819433565532e-4	5.117473427776097e-5	1.5924456790012018	0.1797026502908856	0.7335983792666142
Pseudoflavonifractor capillosus	1.0840396511666639e-4	2.799142408924191e-4	-1.3604647369345806	0.1797026502908856	0.7335983792666142
Scardovia inopinata	0	8.642889323987826e-7	-0.8986254699898454	0.18132978574664457	0.7335983792666142
Ruminococcus gauvreauii	2.9623577450191325e-6	6.732424073977061e-7	1.2437126732612767	0.18169247094327698	0.7335983792666142
Cohcovirus hiberniae	1.4008501234511293e-6	0	1.263545344050305	0.18298684582868605	0.7335983792666142
Clostridium septicum	5.241951651819096e-7	0	0.6080476441783089	0.18298684582868605	0.7335983792666142
Veillonella sp S12025-13	0	4.8426328108735606e-6	-2.5466186237724857	0.1858234726216831	0.7335983792666142
Corynebacterium matruchotii	0	1.732339651371882e-6	-1.4501368331493507	0.1858234726216831	0.7335983792666142
Turicibacter bilis	1.0290271136311365e-4	6.6920462518383235e-6	3.755722054391258	0.18749065982917903	0.7335983792666142
Sutterella wadsworthensis	9.270368250546164e-8	1.36784798856182e-4	-6.9783706943894455	0.19271466826385666	0.7335983792666142
Shigella dysenteriae	0	0	0	0.19485064860886944	0.7335983792666142
Treponema medium	0	0	0	0.19485064860886944	0.7335983792666142
Bacillus altitudinis	0	0	0	0.19485064860886944	0.7335983792666142
Comamonas kerstersii	0	0	0	0.19485064860886944	0.7335983792666142
Mobiluncus curtisii	0	0	0	0.19485064860886944	0.7335983792666142
Niallia circulans	0	0	0	0.19485064860886944	0.7335983792666142
Halanaerobium praevalens	0	0	0	0.19485064860886944	0.7335983792666142
Ralstonia solanacearum	0	0	0	0.19485064860886944	0.7335983792666142
Rhodophyticola sp CCM32	0	0	0	0.19485064860886944	0.7335983792666142
Terrisporobacter hibernicus	0	0	0	0.19485064860886944	0.7335983792666142
Comamonas thiooxydans	0	0	0	0.19485064860886944	0.7335983792666142
Jahgtovirus secundus	0	0	0	0.19485064860886944	0.7335983792666142
Leucobacter chironomi	0	0	0	0.19485064860886944	0.7335983792666142
Paenibacillus curdlanolyticus	0	0	0	0.19485064860886944	0.7335983792666142
Pasteurella multocida	0	0	0	0.19485064860886944	0.7335983792666142
Actinokineospora spheciospongiae	0	0	0	0.19485064860886944	0.7335983792666142
Actinomyces gaoshouyii	0	0	0	0.19485064860886944	0.7335983792666142
Actinotalea ferrariae	0	0	0	0.19485064860886944	0.7335983792666142
Alcaligenes faecalis	0	0	0	0.19485064860886944	0.7335983792666142

Species	Median g1 (resp.)	Median g2 (non-resp.)	log2FC	p	padj
Amylolactobacillus amylophilus	0	0	0	0.19485064860886944	0.7335983792666142
Arenimonas oryziterrae	0	0	0	0.19485064860886944	0.7335983792666142
Bacillus pumilus	0	0	0	0.19485064860886944	0.7335983792666142
Bradyrhizobium sp ORS 285	0	0	0	0.19485064860886944	0.7335983792666142
Brevibacillus aydinogluensis	0	0	0	0.19485064860886944	0.7335983792666142
Burkholderia ubonensis	0	0	0	0.19485064860886944	0.7335983792666142
Carboxydocella thermautotrophica	0	0	0	0.19485064860886944	0.7335983792666142
Corynebacterium breve	0	0	0	0.19485064860886944	0.7335983792666142
Corynebacterium macginleyi	0	0	0	0.19485064860886944	0.7335983792666142
Cutaneotrichosporon curvatum	0	0	0	0.19485064860886944	0.7335983792666142
Dechloromonas denitrificans	0	0	0	0.19485064860886944	0.7335983792666142
Deinococcus soli (ex Cha et al 2016)	0	0	0	0.19485064860886944	0.7335983792666142
Desulfovibrio subterraneus	0	0	0	0.19485064860886944	0.7335983792666142
Devriesea agamarum	0	0	0	0.19485064860886944	0.7335983792666142
Endozoicomonas sp 8E	0	0	0	0.19485064860886944	0.7335983792666142
Erysipelothrix piscisicarius	0	0	0	0.19485064860886944	0.7335983792666142
Gardnerella leopoldii	0	0	0	0.19485064860886944	0.7335983792666142
Geothrix sp	0	0	0	0.19485064860886944	0.7335983792666142
Gordonia phthalatica	0	0	0	0.19485064860886944	0.7335983792666142
Hyphomonas sp CACIAM 19H1	0	0	0	0.19485064860886944	0.7335983792666142
Methanosphaerula palustris	0	0	0	0.19485064860886944	0.7335983792666142
Methylibium petroleiphilum	0	0	0	0.19485064860886944	0.7335983792666142
Methylobacterium radiodurans	0	0	0	0.19485064860886944	0.7335983792666142
Microbacterium arborescens	0	0	0	0.19485064860886944	0.7335983792666142
Microbacterium binotii	0	0	0	0.19485064860886944	0.7335983792666142
Mycolicibacterium frederiksbergense	0	0	0	0.19485064860886944	0.7335983792666142
Neorhizobium vignae	0	0	0	0.19485064860886944	0.7335983792666142
Nitratidesulfovibrio vulgaris	0	0	0	0.19485064860886944	0.7335983792666142
Nocardia sp NBC 01503	0	0	0	0.19485064860886944	0.7335983792666142
Ornithinimicrobium pratense	0	0	0	0.19485064860886944	0.7335983792666142
Paenibacillus mucilaginosus	0	0	0	0.19485064860886944	0.7335983792666142
Paenibacillus sp 32O-W	0	0	0	0.19485064860886944	0.7335983792666142
Paenibacillus sp H1-7	0	0	0	0.19485064860886944	0.7335983792666142
Parafrankia discariae	0	0	0	0.19485064860886944	0.7335983792666142

Species	Median g1 (resp.)	Median g2 (non-resp.)	log2FC	p	padj
Paramagnetospirillum caucaseum	0	0	0	0.19485064860886944	0.7335983792666142
Plesiocystis pacifica	0	0	0	0.19485064860886944	0.7335983792666142
Polaromonas naphthalenivorans	0	0	0	0.19485064860886944	0.7335983792666142
Pseudarthrobacter psychrotolerans	0	0	0	0.19485064860886944	0.7335983792666142
Rhodococcus sp NBC 00294	0	0	0	0.19485064860886944	0.7335983792666142
Rickettsia tillamookensis	0	0	0	0.19485064860886944	0.7335983792666142
Sphingomonas sp M1-B02	0	0	0	0.19485064860886944	0.7335983792666142
Streptomyces globosus	0	0	0	0.19485064860886944	0.7335983792666142
Streptomyces sp SM17	0	0	0	0.19485064860886944	0.7335983792666142
Synechococcus sp A15-28	0	0	0	0.19485064860886944	0.7335983792666142
Tessaracoccus timonensis	0	0	0	0.19485064860886944	0.7335983792666142
Venatoribacter cucullus	0	0	0	0.19485064860886944	0.7335983792666142
Acetobacterium wieringae	0	0	0	0.19485064860886944	0.7335983792666142
Alicyclobacillus pomorum	0	0	0	0.19485064860886944	0.7335983792666142
Aminipila terrae	0	0	0	0.19485064860886944	0.7335983792666142
Azorhizobium caulinodans	0	0	0	0.19485064860886944	0.7335983792666142
Bosea sp 685	0	0	0	0.19485064860886944	0.7335983792666142
Buchavirus faecalis	0	0	0	0.19485064860886944	0.7335983792666142
Clostridium isatidis	0	0	0	0.19485064860886944	0.7335983792666142
Clostridium tyrobutyricum	0	0	0	0.19485064860886944	0.7335983792666142
Crassaminicella indica	0	0	0	0.19485064860886944	0.7335983792666142
Culoivirus faecalis	0	0	0	0.19485064860886944	0.7335983792666142
Cytobacillus gottheilii	0	0	0	0.19485064860886944	0.7335983792666142
Defluviicoccus vanus	0	0	0	0.19485064860886944	0.7335983792666142
Desulfogranum marinum	0	0	0	0.19485064860886944	0.7335983792666142
Desulfosediminicola ganghwensis	0	0	0	0.19485064860886944	0.7335983792666142
Diaphorobacter sp HDW4A	0	0	0	0.19485064860886944	0.7335983792666142
Drivevirus gastrointestinalis	0	0	0	0.19485064860886944	0.7335983792666142
Dyella caseinilytica	0	0	0	0.19485064860886944	0.7335983792666142
Edwardsiella piscicida	0	0	0	0.19485064860886944	0.7335983792666142
Effusibacillus pohliae	0	0	0	0.19485064860886944	0.7335983792666142
Erythrobacter longus	0	0	0	0.19485064860886944	0.7335983792666142
Geothrix sp 21YS21S-2	0	0	0	0.19485064860886944	0.7335983792666142
Geothrix sp PMB-07	0	0	0	0.19485064860886944	0.7335983792666142

Species	Median g1 (resp.)	Median g2 (non-resp.)	log2FC	p	padj
Gordonia insulae	0	0	0	0.19485064860886944	0.7335983792666142
Haliangium ochraceum	0	0	0	0.19485064860886944	0.7335983792666142
Herbaspirillum rubrisubalbicans	0	0	0	0.19485064860886944	0.7335983792666142
Histoplasma capsulatum	0	0	0	0.19485064860886944	0.7335983792666142
Hyphomonas atlantica	0	0	0	0.19485064860886944	0.7335983792666142
Janibacter melonis	0	0	0	0.19485064860886944	0.7335983792666142
Janibacter sp CX7	0	0	0	0.19485064860886944	0.7335983792666142
Kitasatospora sp HUAS 3-15	0	0	0	0.19485064860886944	0.7335983792666142
Leifsonia sp 21MFCrub1 1	0	0	0	0.19485064860886944	0.7335983792666142
Niveibacterium microcysteis	0	0	0	0.19485064860886944	0.7335983792666142
Nocardia cyriacigeorgica	0	0	0	0.19485064860886944	0.7335983792666142
Oxalobacter vibrioformis	0	0	0	0.19485064860886944	0.7335983792666142
Paenibacillus albus	0	0	0	0.19485064860886944	0.7335983792666142
Paenibacillus marchantiae	0	0	0	0.19485064860886944	0.7335983792666142
Pantoea dispersa	0	0	0	0.19485064860886944	0.7335983792666142
Penicillium brevicompactum	0	0	0	0.19485064860886944	0.7335983792666142
Penicillium solitum	0	0	0	0.19485064860886944	0.7335983792666142
Piscinibacter gummiphilus	0	0	0	0.19485064860886944	0.7335983792666142
Pseudolysobacter antarcticus	0	0	0	0.19485064860886944	0.7335983792666142
Pseudomonas congelans	0	0	0	0.19485064860886944	0.7335983792666142
Pseudomonas sp SCB32	0	0	0	0.19485064860886944	0.7335983792666142
Pseudoxanthomonas sp JBR18	0	0	0	0.19485064860886944	0.7335983792666142
Rhinocladiella mackenziei	0	0	0	0.19485064860886944	0.7335983792666142
Rhizopus stolonifer	0	0	0	0.19485064860886944	0.7335983792666142
Rhodopseudomonas palustris	0	0	0	0.19485064860886944	0.7335983792666142
Rhodotorula mucilaginosa	0	0	0	0.19485064860886944	0.7335983792666142
Salmonella sp SSDFZ54	0	0	0	0.19485064860886944	0.7335983792666142
Serratia entomophila	0	0	0	0.19485064860886944	0.7335983792666142
Skunavirus i0139	0	0	0	0.19485064860886944	0.7335983792666142
Stigmatella aurantiaca	0	0	0	0.19485064860886944	0.7335983792666142
Stratiformator vulcanicus	0	0	0	0.19485064860886944	0.7335983792666142
Streptomyces sp NBC 00669	0	0	0	0.19485064860886944	0.7335983792666142
Streptomyces sp NBC 01244	0	0	0	0.19485064860886944	0.7335983792666142
Streptomyces sp NHF165	0	0	0	0.19485064860886944	0.7335983792666142

Species	Median g1 (resp.)	Median g2 (non-resp.)	log2FC	p	padj
Sulfuritalea hydrogenivorans	0	0	0	0.19485064860886944	0.7335983792666142
Suspvirus SUSP2	0	0	0	0.19485064860886944	0.7335983792666142
Talaromyces sp PF30-5	0	0	0	0.19485064860886944	0.7335983792666142
Thiomonas intermedia	0	0	0	0.19485064860886944	0.7335983792666142
Vibrio aerogenes	0	0	0	0.19485064860886944	0.7335983792666142
Williamsoniiplasma luminosum	0	0	0	0.19485064860886944	0.7335983792666142
Yersinia hibernica	0	0	0	0.19485064860886944	0.7335983792666142
Akkermansia sp AE01SA01	0	0	0	0.19485064860886944	0.7335983792666142
Buchavirus coli	0	0	0	0.19485064860886944	0.7335983792666142
Buchavirus hiberniae	0	0	0	0.19485064860886944	0.7335983792666142
Buchavirus hominis	0	0	0	0.19485064860886944	0.7335983792666142
Buchavirus oralis	0	0	0	0.19485064860886944	0.7335983792666142
Buchavirus splanchnicus	0	0	0	0.19485064860886944	0.7335983792666142
Chryseobacterium gallinarum	0	0	0	0.19485064860886944	0.7335983792666142
Paenibacillus sp FSL R7-0273	0	0	0	0.19485064860886944	0.7335983792666142
Acetivibrio thermocellus	0	0	0	0.19485064860886944	0.7335983792666142
Acetobacterium sp KB-1	0	0	0	0.19485064860886944	0.7335983792666142
Actinomadura citrea	0	0	0	0.19485064860886944	0.7335983792666142
Aerococcus christensenii	0	0	0	0.19485064860886944	0.7335983792666142
Aeromonas sp ASNIH1	0	0	0	0.19485064860886944	0.7335983792666142
Aquaspirillum serpens	0	0	0	0.19485064860886944	0.7335983792666142
Bordetella trematum	0	0	0	0.19485064860886944	0.7335983792666142
Burkholderia multivorans	0	0	0	0.19485064860886944	0.7335983792666142
Caldimonas thermodepolymerans	0	0	0	0.19485064860886944	0.7335983792666142
Campylobacter corcagiensis	0	0	0	0.19485064860886944	0.7335983792666142
Citrobacter werkmanii	0	0	0	0.19485064860886944	0.7335983792666142
Comamonas testosteroni	0	0	0	0.19485064860886944	0.7335983792666142
Couchioplanes caeruleus	0	0	0	0.19485064860886944	0.7335983792666142
Desulfatibacillum aliphaticivorans	0	0	0	0.19485064860886944	0.7335983792666142
Halomonas sp 18071143	0	0	0	0.19485064860886944	0.7335983792666142
Haploplasma axanthum	0	0	0	0.19485064860886944	0.7335983792666142
Heyndrickxia acidiproducens	0	0	0	0.19485064860886944	0.7335983792666142
Inquilinus limosus	0	0	0	0.19485064860886944	0.7335983792666142
Kluyvera genomosp 3	0	0	0	0.19485064860886944	0.7335983792666142

Species	Median g1 (resp.)	Median g2 (non-resp.)	log2FC	p	padj
Kosakonia oryzendophytica	0	0	0	0.19485064860886944	0.7335983792666142
Lentzea sp NBC 00516	0	0	0	0.19485064860886944	0.7335983792666142
Listeria newyorkensis	0	0	0	0.19485064860886944	0.7335983792666142
Methanoplanus limicola	0	0	0	0.19485064860886944	0.7335983792666142
Methylococcus sp Mc7	0	0	0	0.19485064860886944	0.7335983792666142
Parazoarcus communis	0	0	0	0.19485064860886944	0.7335983792666142
Pedobacter antarcticus	0	0	0	0.19485064860886944	0.7335983792666142
Phototrophicus methaneseepsis	0	0	0	0.19485064860886944	0.7335983792666142
Planococcus sp N016	0	0	0	0.19485064860886944	0.7335983792666142
Polynucleobacter sp AP-Titi-500A-B4	0	0	0	0.19485064860886944	0.7335983792666142
Prochlorococcus marinus	0	0	0	0.19485064860886944	0.7335983792666142
Pseudomonas sp ABC1	0	0	0	0.19485064860886944	0.7335983792666142
Pseudothauera hydrothermalis	0	0	0	0.19485064860886944	0.7335983792666142
Rheinheimera mangrovi	0	0	0	0.19485064860886944	0.7335983792666142
Rhizobium sp NXC14	0	0	0	0.19485064860886944	0.7335983792666142
Saksenaea vasiformis	0	0	0	0.19485064860886944	0.7335983792666142
Salinibacterium sp ZJ70	0	0	0	0.19485064860886944	0.7335983792666142
Scedosporium boydii	0	0	0	0.19485064860886944	0.7335983792666142
Sinimarinibacterium sp NLF-5-8	0	0	0	0.19485064860886944	0.7335983792666142
Solwaraspora sp WMMD791	0	0	0	0.19485064860886944	0.7335983792666142
Streptococcus marimammalium	0	0	0	0.19485064860886944	0.7335983792666142
Streptomyces liliifuscus	0	0	0	0.19485064860886944	0.7335983792666142
Stutzerimonas balearica	0	0	0	0.19485064860886944	0.7335983792666142
Sulfurimicrobium lacus	0	0	0	0.19485064860886944	0.7335983792666142
Thauera terpenica	0	0	0	0.19485064860886944	0.7335983792666142
Verminephrobacter eiseniae	0	0	0	0.19485064860886944	0.7335983792666142
Acinetobacter johnsonii	0	0	0	0.19485064860886944	0.7335983792666142
Aeromicrobium senzhongii	0	0	0	0.19485064860886944	0.7335983792666142
Caballeronia sp TF1N1	0	0	0	0.19485064860886944	0.7335983792666142
Campylobacter geochelonis	0	0	0	0.19485064860886944	0.7335983792666142
Chryseobacterium luteum	0	0	0	0.19485064860886944	0.7335983792666142
Dietzia kunjamensis	0	0	0	0.19485064860886944	0.7335983792666142
Enterobacter bugandensis	0	0	0	0.19485064860886944	0.7335983792666142
Enterobacter cancerogenus	0	0	0	0.19485064860886944	0.7335983792666142

Species	Median g1 (resp.)	Median g2 (non-resp.)	log2FC	p	padj
Frigoriglobus tundricola	0	0	0	0.19485064860886944	0.7335983792666142
Hymenobacter yonginensis	0	0	0	0.19485064860886944	0.7335983792666142
Janthinobacterium agaricidamnosum	0	0	0	0.19485064860886944	0.7335983792666142
Jeotgalibaca sp PTS2502	0	0	0	0.19485064860886944	0.7335983792666142
Lacticaseibacillus sp NCIMB 15471	0	0	0	0.19485064860886944	0.7335983792666142
Lactobacillus gigeriorum	0	0	0	0.19485064860886944	0.7335983792666142
Lactobacillus intestinalis	0	0	0	0.19485064860886944	0.7335983792666142
Lactobacillus phage KC5a	0	0	0	0.19485064860886944	0.7335983792666142
Lactobacillus sp IBH004	0	0	0	0.19485064860886944	0.7335983792666142
Listeria grayi	0	0	0	0.19485064860886944	0.7335983792666142
Marichromatium purpuratum	0	0	0	0.19485064860886944	0.7335983792666142
Neisseria sp RH3002v2g	0	0	0	0.19485064860886944	0.7335983792666142
Providencia sneebia	0	0	0	0.19485064860886944	0.7335983792666142
Providencia stuartii	0	0	0	0.19485064860886944	0.7335983792666142
Serratia proteamaculans	0	0	0	0.19485064860886944	0.7335983792666142
Sphaerospermopsis torques-reginae	0	0	0	0.19485064860886944	0.7335983792666142
Streptococcus devriesei	0	0	0	0.19485064860886944	0.7335983792666142
Streptococcus himalayensis	0	0	0	0.19485064860886944	0.7335983792666142
Streptomyces sp NBC 00358	0	0	0	0.19485064860886944	0.7335983792666142
Subtercola endophyticus	0	0	0	0.19485064860886944	0.7335983792666142
Synergistes jonesii	0	0	0	0.19485064860886944	0.7335983792666142
Tellurirhabdus bombi	0	0	0	0.19485064860886944	0.7335983792666142
Thermoascus crustaceus	0	0	0	0.19485064860886944	0.7335983792666142
Verrucosispora sp WMMD1129	0	0	0	0.19485064860886944	0.7335983792666142
Vibrio parahaemolyticus	0	0	0	0.19485064860886944	0.7335983792666142
Vibrio sp HDW18	0	0	0	0.19485064860886944	0.7335983792666142
Yersinia massiliensis	0	0	0	0.19485064860886944	0.7335983792666142
Eponavirus epona	0	0	0	0.19485064860886944	0.7335983792666142
Limosilactobacillus antri	0	0	0	0.19485064860886944	0.7335983792666142
Abyssogena phaseoliformis symbiont	0	0	0	0.19485064860886944	0.7335983792666142
Actinomycetospora chiangmaiensis	0	0	0	0.19485064860886944	0.7335983792666142
Aeromonas media	0	0	0	0.19485064860886944	0.7335983792666142
Alkalihalobacterium bogoriense	0	0	0	0.19485064860886944	0.7335983792666142
Burkholderia cepacia	0	0	0	0.19485064860886944	0.7335983792666142

Species	Median g1 (resp.)	Median g2 (non-resp.)	log2FC	p	padj
Cetobacterium somerae	0	0	0	0.19485064860886944	0.7335983792666142
Citrobacter sp 172116965	0	0	0	0.19485064860886944	0.7335983792666142
Citrobacter sp RHBSTW-00524	0	0	0	0.19485064860886944	0.7335983792666142
Citrobacter sp RHBSTW-00881	0	0	0	0.19485064860886944	0.7335983792666142
Citrobacter sp RHBSTW-00976	0	0	0	0.19485064860886944	0.7335983792666142
Clostridium phage CI461P1	0	0	0	0.19485064860886944	0.7335983792666142
Curvularia papendorfii	0	0	0	0.19485064860886944	0.7335983792666142
Enterococcus sp CR-Ec1	0	0	0	0.19485064860886944	0.7335983792666142
Enterococcus sp FDAARGOS 375	0	0	0	0.19485064860886944	0.7335983792666142
Erysipelothrix rhusiopathiae	0	0	0	0.19485064860886944	0.7335983792666142
Faecalibacter bovis	0	0	0	0.19485064860886944	0.7335983792666142
Flavobacterium aestivum	0	0	0	0.19485064860886944	0.7335983792666142
Fusobacterium hominis	0	0	0	0.19485064860886944	0.7335983792666142
Gardnerella piotii	0	0	0	0.19485064860886944	0.7335983792666142
Halobacillus kuroshimensis	0	0	0	0.19485064860886944	0.7335983792666142
Halocella sp SP3-1	0	0	0	0.19485064860886944	0.7335983792666142
Jiaodavirus kp179	0	0	0	0.19485064860886944	0.7335983792666142
Jiaodavirus kppv15	0	0	0	0.19485064860886944	0.7335983792666142
Jiaodavirus kpv477	0	0	0	0.19485064860886944	0.7335983792666142
Jiaodavirus mineola	0	0	0	0.19485064860886944	0.7335983792666142
Jiaodavirus pko111	0	0	0	0.19485064860886944	0.7335983792666142
Kitasatospora purpeofusca	0	0	0	0.19485064860886944	0.7335983792666142
Klebsiella sp w6	0	0	0	0.19485064860886944	0.7335983792666142
Leclercia sp LSNIH1	0	0	0	0.19485064860886944	0.7335983792666142
Megalodesulfovibrio gigas	0	0	0	0.19485064860886944	0.7335983792666142
Mycobacterium basiliense	0	0	0	0.19485064860886944	0.7335983792666142
Nocardia terpenica	0	0	0	0.19485064860886944	0.7335983792666142
Paenibacillus sp BIHB 4019	0	0	0	0.19485064860886944	0.7335983792666142
Pedobacter sp D749	0	0	0	0.19485064860886944	0.7335983792666142
Peptoclostridium acidaminophilum	0	0	0	0.19485064860886944	0.7335983792666142
Petrocella atlantisensis	0	0	0	0.19485064860886944	0.7335983792666142
Pluralibacter gergoviae	0	0	0	0.19485064860886944	0.7335983792666142
Pseudoalteromonas aliena	0	0	0	0.19485064860886944	0.7335983792666142
Pseudomonas sp BIOMIG1BAC	0	0	0	0.19485064860886944	0.7335983792666142

Species	Median g1 (resp.)	Median g2 (non-resp.)	log2FC	p	padj
Rhodobacter capsulatus	0	0	0	0.19485064860886944	0.7335983792666142
Rhodonellum psychrophilum	0	0	0	0.19485064860886944	0.7335983792666142
Salmonella bongori	0	0	0	0.19485064860886944	0.7335983792666142
Streptomyces sp GS7	0	0	0	0.19485064860886944	0.7335983792666142
Sulfurimonas paralvinellae	0	0	0	0.19485064860886944	0.7335983792666142
Sulfurospirillum multivorans	0	0	0	0.19485064860886944	0.7335983792666142
Treponema putidum	0	0	0	0.19485064860886944	0.7335983792666142
Vitreoscilla filiformis	0	0	0	0.19485064860886944	0.7335983792666142
Allobaculum stercoricanis	0	0	0	0.19485064860886944	0.7335983792666142
Amycolatopsis decaplanina	0	0	0	0.19485064860886944	0.7335983792666142
Anaerovibrio lipolyticus	0	0	0	0.19485064860886944	0.7335983792666142
Aspergillus sclerotiorum	0	0	0	0.19485064860886944	0.7335983792666142
Bartonella apihabitans	0	0	0	0.19485064860886944	0.7335983792666142
Botryosphaeria dothidea	0	0	0	0.19485064860886944	0.7335983792666142
Brevibacillus brevis	0	0	0	0.19485064860886944	0.7335983792666142
Burkholderia contaminans	0	0	0	0.19485064860886944	0.7335983792666142
Capnocytophaga canimorsus	0	0	0	0.19485064860886944	0.7335983792666142
Chelativorans sp AA-79	0	0	0	0.19485064860886944	0.7335983792666142
Chryseobacterium oranimense	0	0	0	0.19485064860886944	0.7335983792666142
Citrobacter sp RHBSTW-00903	0	0	0	0.19485064860886944	0.7335983792666142
Clavibacter michiganensis	0	0	0	0.19485064860886944	0.7335983792666142
Corynebacterium riegelii	0	0	0	0.19485064860886944	0.7335983792666142
Dolichospermum flos-aquae	0	0	0	0.19485064860886944	0.7335983792666142
Emergomyces orientalis	0	0	0	0.19485064860886944	0.7335983792666142
Enterococcus rotai	0	0	0	0.19485064860886944	0.7335983792666142
Erysipelothrix sp HDW6B	0	0	0	0.19485064860886944	0.7335983792666142
Geoalkalibacter halelectricus	0	0	0	0.19485064860886944	0.7335983792666142
Klebsiella sp WP8-S18-ESBL-06	0	0	0	0.19485064860886944	0.7335983792666142
Madurella mycetomatis	0	0	0	0.19485064860886944	0.7335983792666142
Massilia sp YMA4	0	0	0	0.19485064860886944	0.7335983792666142
Mesorhizobium sp NBSH29	0	0	0	0.19485064860886944	0.7335983792666142
Mesorhizobium terrae	0	0	0	0.19485064860886944	0.7335983792666142
Methylorubrum extorquens	0	0	0	0.19485064860886944	0.7335983792666142
Microvirga sp 17 mud 1-3	0	0	0	0.19485064860886944	0.7335983792666142

Species	Median g1 (resp.)	Median g2 (non-resp.)	log2FC	p	padj
Mycobacterium lacus	0	0	0	0.19485064860886944	0.7335983792666142
Nakamurella multipartita	0	0	0	0.19485064860886944	0.7335983792666142
Neokomagataea tanensis	0	0	0	0.19485064860886944	0.7335983792666142
Neorhizobium galegae	0	0	0	0.19485064860886944	0.7335983792666142
Oceanobacillus picturae	0	0	0	0.19485064860886944	0.7335983792666142
Pasteurella canis	0	0	0	0.19485064860886944	0.7335983792666142
Pediococcus claussenii	0	0	0	0.19485064860886944	0.7335983792666142
Pseudomonas benzenivorans	0	0	0	0.19485064860886944	0.7335983792666142
Scedosporium aurantiacum	0	0	0	0.19485064860886944	0.7335983792666142
Streptomyces rapamycinicus	0	0	0	0.19485064860886944	0.7335983792666142
Streptomyces sp ITFR-21	0	0	0	0.19485064860886944	0.7335983792666142
Streptomyces sp NBC 00376	0	0	0	0.19485064860886944	0.7335983792666142
Trichoderma harzianum	0	0	0	0.19485064860886944	0.7335983792666142
Wickerhamiella sorbophila	0	0	0	0.19485064860886944	0.7335983792666142
anthomonas vesicatoria	0	0	0	0.19485064860886944	0.7335983792666142
Arachnia rubra	1.6991801522744168e-6	5.3668882755805896e-6	-1.2380671781337003	0.20374074977591847	0.7335983792666142
Streptococcus sp S5	5.054556354006974e-6	7.763747250129612e-6	-0.5335266263432088	0.2051522023747494	0.7335983792666142
Olsenella uli	7.902374439585838e-5	4.935165382574007e-6	3.7530679994532035	0.20585563743408689	0.7335983792666142
Coprobacter fastidiosus	0	1.4769221035785062e-6	-1.3085484981571067	0.21103083214626514	0.7335983792666142
Bacteroides caccae	0.011808900512824861	5.486784220513211e-4	4.425265154580231	0.21288515406162467	0.7335983792666142
Lachnoclostridium phocaeense	3.039074652019996e-5	4.184937805429039e-5	-0.4489349253758536	0.21288515406162467	0.7335983792666142
Anaerococcus hydrogenalis	9.270368250546164e-8	0	0.1279022265007203	0.2132880129975615	0.7335983792666142
Akkermansia massiliensis	0	0	0	0.2197724356511933	0.7335983792666142
Hallella multisaccharivorax	0	0	0	0.2197724356511933	0.7335983792666142
Aggregatibacter sp 2125159857	0	0	0	0.22043638574536195	0.7335983792666142
Aggregatibacter sp oral taxon 513	0	0	0	0.22043638574536195	0.7335983792666142
Candidatus Minimicrobia vallesae	0	0	0	0.22043638574536195	0.7335983792666142
Alloscardovia criceti	0	0	0	0.22043638574536195	0.7335983792666142
Bifidobacterium cuniculi	0	0	0	0.22043638574536195	0.7335983792666142
Bifidobacterium eulemuris	0	0	0	0.22043638574536195	0.7335983792666142
Corynebacterium amycolatum	0	0	0	0.22043638574536195	0.7335983792666142
Corynebacterium variabile	0	0	0	0.22043638574536195	0.7335983792666142
Cutibacterium avidum	0	0	0	0.22043638574536195	0.7335983792666142
Helcococcus kunzii	0	0	0	0.22043638574536195	0.7335983792666142

Species	Median g1 (resp.)	Median g2 (non-resp.)	log2FC	p	padj
Pyramidobacter sp YE332	0	0	0	0.22043638574536195	0.7335983792666142
Staphylococcus xylosus	0	0	0	0.22043638574536195	0.7335983792666142
Streptococcus downei	0	0	0	0.22043638574536195	0.7335983792666142
Streptococcus phage PH15	0	0	0	0.22043638574536195	0.7335983792666142
Streptococcus sp NCTC 11567	0	0	0	0.22043638574536195	0.7335983792666142
Caproicibacterium amylolyticum	0	0	0	0.22043638574536195	0.7335983792666142
Acidilutibacter cellobiosedens	0	0	0	0.22043638574536195	0.7335983792666142
Lactobacillus hominis	0	0	0	0.22043638574536195	0.7335983792666142
Lactococcus sp NH2-7C	0	0	0	0.22043638574536195	0.7335983792666142
Loigolactobacillus coryniformis	0	0	0	0.22043638574536195	0.7335983792666142
Aristaeella hokkaidonensis	0	0	0	0.22043638574536195	0.7335983792666142
Capnocytophaga gingivalis	0	0	0	0.22043638574536195	0.7335983792666142
Citrobacter farmeri	0	0	0	0.22043638574536195	0.7335983792666142
Enterococcus malodoratus	0	0	0	0.22043638574536195	0.7335983792666142
Enterococcus thailandicus	0	0	0	0.22043638574536195	0.7335983792666142
Neisseria elongata	0	0	0	0.22043638574536195	0.7335983792666142
Tannerella serpentiformis	0	0	0	0.22043638574536195	0.7335983792666142
Tetragenococcus halophilus	0	0	0	0.22043638574536195	0.7335983792666142
Pseudoprevotella muciniphila	0	0	0	0.22043638574536195	0.7335983792666142
Selenomonas sp oral taxon 920	0	0	0	0.22043638574536195	0.7335983792666142
Treponema parvum	0	0	0	0.22043638574536195	0.7335983792666142
Actinotignum timonense	0	0	0	0.22043638574536195	0.7335983792666142
Campylobacter curvus	0	0	0	0.22043638574536195	0.7335983792666142
Fusobacterium sp oral taxon 203	0	0	0	0.22043638574536195	0.7335983792666142
Hoylesella saccharolytica	0	0	0	0.22043638574536195	0.7335983792666142
Leptotrichia wadei	0	0	0	0.22043638574536195	0.7335983792666142
Prevotella micans	0	0	0	0.22043638574536195	0.7335983792666142
Segatella oulorum	0	0	0	0.22043638574536195	0.7335983792666142
Variovorax paradoxus	0	0	0	0.22043638574536195	0.7335983792666142
Schaalia radingae	0	0	0	0.22043638574536195	0.7335983792666142
Taenia solium	0	0	0	0.22043638574536195	0.7335983792666142
Anaerocolumna sp AGMB13020	0	0	0	0.22043638574536195	0.7335983792666142
Bifidobacterium pseudolongum	0	0	0	0.22043638574536195	0.7335983792666142
Aspergillus latus	0	0	0	0.22043638574536195	0.7335983792666142

Species	Median g1 (resp.)	Median g2 (non-resp.)	log2FC	p	padj
Bifidobacterium reuteri	0	0	0	0.22043638574536195	0.7335983792666142
Fusobacterium gonidiaformans	0	0	0	0.22043638574536195	0.7335983792666142
uncultured Desulfobacter sp	0	0	0	0.22043638574536195	0.7335983792666142
Mahlunavirus rarus	0	0	0	0.22043638574536195	0.7335983792666142
Treponema vincentii	0	0	0	0.22043638574536195	0.7335983792666142
Campylobacter rectus	0	0	0	0.22043638574536195	0.7335983792666142
Planococcus halotolerans	0	0	0	0.22043638574536195	0.7335983792666142
Corynebacterium tuberculostearicum	7.430370645610722e-7	0	0.8016032477710648	0.22054301342015367	0.7335983792666142
Streptococcus sp DTU 2020 1001019 1 SI AUS MUR 006	0	4.913467331387013e-7	-0.5766157184022308	0.22056273122887296	0.7335983792666142
Veillonella parvula	2.6213316973451214e-5	2.929683727833333e-4	-3.4332762285217457	0.22315166548585114	0.7335983792666142
Actinomyces sp oral taxon 171	2.180869434708745e-6	1.5229080357252739e-5	-2.3510881901030665	0.22315166548585114	0.7335983792666142
Lactiplantibacillus plantarum	2.7811104751638493e-7	2.9120832692628917e-6	-1.6139238896137158	0.22777811082820532	0.7335983792666142
Romboutsia ilealis	0.0011705636770300354	3.300419844317494e-5	5.106578675140419	0.23307853957687966	0.7335983792666142
Staphylococcus aureus	0	4.771798290360108e-7	-0.5628454677181727	0.23703098025642214	0.7335983792666142
Scardovia wiggsiae	9.420680666661968e-7	1.3074044151607758e-5	-2.8573712728837344	0.23926721569120155	0.7335983792666142
Phocaeicola sartorii	2.0151080361734893e-5	1.3221679965869703e-4	-2.6549727679909774	0.2407331955466823	0.7335983792666142
Lactobacillus delbrueckii	1.038372873224126e-6	8.523627332828506e-6	-2.2240931845279994	0.2407331955466823	0.7335983792666142
Parabacteroides johnsonii	2.6853034139928408e-5	7.85532174300708e-6	1.6532178636333725	0.24146341472869742	0.7335983792666142
Streptococcus intermedius	1.210156601140848e-6	4.3509431553695224e-6	-1.2756446071317713	0.24146341472869742	0.7335983792666142
Bacteroides thetaiotaomicron	0.021228619444551834	0.0018832558381309391	3.494011742375302	0.24962292609351436	0.7335983792666142
Bifidobacterium adolescentis	3.6643701746266885e-4	0.003591550899886856	-3.2894397139064937	0.24962292609351436	0.7335983792666142
Phocaeicola dorei	0.008148932553433845	0.001558480222879549	2.385722863290047	0.24962292609351436	0.7335983792666142
Bacteroides uniformis	0.003150573758794434	0.007344883651037122	-1.2208636182224653	0.24962292609351436	0.7335983792666142
Clostridioides difficile	1.7908680911341598e-4	3.4227675362923695e-4	-0.9306796512007977	0.24962292609351436	0.7335983792666142
Streptococcus parasanguinis	3.093532875354782e-4	5.13349219931219e-4	-0.7288367886890466	0.24962292609351436	0.7335983792666142
Faecalibacterium sp I3-3-33	4.1224529286861224e-5	2.5322795971426783e-5	0.6817686018455016	0.24962292609351436	0.7335983792666142
Streptococcus parasuis	9.420680666661968e-7	0	0.9575937660098746	0.24986511409397325	0.7335983792666142
Peptoniphilus duerdenii	0	3.780193334757782e-7	-0.4625961290537894	0.24986511409397325	0.7335983792666142
Kineothrix sp MB12-C1	2.2995864423742035e-7	0	0.2986098076322935	0.260146873686418	0.7335983792666142
Thomasclavelia spiroformis	1.446452334574068e-6	0	1.2906911743125367	0.26332947003103757	0.7335983792666142
Granulicatella elegans	3.337790716011397e-7	0	0.41551971697112833	0.26332947003103757	0.7335983792666142
Intestinibaculum porci	2.527278177003487e-7	0	0.3250729919067554	0.26332947003103757	0.7335983792666142
Klebsiella grimontii	0	2.125796110643436e-7	-0.27807946954083773	0.26332947003103757	0.7335983792666142
Anaerococcus senegalensis	4.82150778191356e-7	0	0.5676922194953893	0.2646037266168433	0.7335983792666142

Species	Median g1 (resp.)	Median g2 (non-resp.)	log2FC	p	padj
Proteiniclasticum sp QWL-01	2.2995864423742035e-7	0	0.2986098076322935	0.2646037266168433	0.7335983792666142
Veillonella dispar	5.054556354006974e-7	1.1316882129913062e-5	-3.0323650026991347	0.26716541030483754	0.7335983792666142
Romboutsia sp CE17	6.94157400684113e-5	2.380646072183957e-6	4.380527061211533	0.27261131678146394	0.7335983792666142
Romboutsia sp 13368	2.2854201986870387e-5	1.35329061584014e-6	3.3414920235520422	0.2764344152459361	0.7335983792666142
Prevotella bivia	8.918896931383542e-6	1.237829373204915e-6	2.1480796491782543	0.2764344152459361	0.7335983792666142
Haemophilus sputorum	0	0	0	0.2796047974997405	0.7335983792666142
Veillonella seminalis	0	0	0	0.2796047974997405	0.7335983792666142
Bifidobacterium mongoliense	0	0	0	0.2796047974997405	0.7335983792666142
Sporopachydermia lactativora	0	0	0	0.2796047974997405	0.7335983792666142
Terrisporobacter mayombei	0	0	0	0.2796047974997405	0.7335983792666142
Canhaevirus faecalis	0	0	0	0.2796047974997405	0.7335983792666142
Faecalimicrobium dakarense	0	0	0	0.2796047974997405	0.7335983792666142
Kahnovirus oralis	0	0	0	0.2796047974997405	0.7335983792666142
Streptococcus infantarius	0	0	0	0.2796047974997405	0.7335983792666142
iamenia xianingshaonis	0	0	0	0.2796047974997405	0.7335983792666142
Ethanoligenens harbinense	0	0	0	0.2796047974997405	0.7335983792666142
Sarcina sp JB2	0	0	0	0.2796047974997405	0.7335983792666142
Methanobrevibacter sp TLL-48-HuF1	0	1.0708310327878015e-5	-3.5494609847761622	0.28031163502943507	0.7335983792666142
Clostridium sartagoforme	3.337790716011397e-7	0	0.41551971697112833	0.28031163502943507	0.7335983792666142
Enterococcus gilvus	0	2.5275681862069594e-7	-0.3251063902228827	0.28031163502943507	0.7335983792666142
Streptococcus sp LPB0220	7.612226489396345e-6	1.3411041506696772e-5	-0.7427164389669563	0.28045238545454537	0.7335983792666142
Streptococcus sp S1	3.435044981271927e-6	4.57208446967726e-6	-0.32926839179927575	0.28045238545454537	0.7335983792666142
Streptococcus sp FDAARGOS 192	1.873225046821169e-5	1.7664823363744188e-5	0.08023364955374139	0.2811989624284306	0.7335983792666142
Oscillibacter hominis	9.46575086873348e-5	3.032718852644479e-4	-1.6694109116775753	0.29077784960137903	0.7335983792666142
Blautia obeum	0.014952973580457458	0.006371243378263107	1.2306556147156869	0.29077784960137903	0.7335983792666142
Schaalia odontolytica	2.0304541003052072e-4	4.5564082001942015e-4	-1.1621695729068557	0.29077784960137903	0.7335983792666142
[Clostridium] scindens	0.001105621069143212	6.755802397024708e-4	0.7098283495607084	0.29077784960137903	0.7335983792666142
Oscillibacter sp PEA192	1.968567035498624e-4	2.815007027673602e-4	-0.5137985106275964	0.29077784960137903	0.7335983792666142
Peptoniphilus harei	5.311817990923596e-6	0	2.658055604055768	0.29343398212915317	0.7335983792666142
Prevotella amnii	0	1.081513140613345e-6	-1.057632666255151	0.29343398212915317	0.7335983792666142
Novisyntrophococcus fermenticellae	9.198345769496814e-7	2.256116834179555e-6	-0.7621704604119516	0.2940077869915829	0.7335983792666142
Bacteroides faecium	1.9523730506920216e-5	9.412404469891235e-7	3.4022422569041804	0.3003878986828706	0.7335983792666142
Turicibacter sp TJ11	2.892904669148136e-6	0	1.9608470158819093	0.30385148400424855	0.7335983792666142
Streptococcus phage YMC-2011	1.769094723902441e-6	0	1.469414405588887	0.30385148400424855	0.7335983792666142

Species	Median g1 (resp.)	Median g2 (non-resp.)	log2FC	p	padj
Peptoniphilus lacrimalis	1.314405548133816e-6	1.3051490741272467e-7	1.033661670681094	0.3110000785084484	0.7335983792666142
Schaalia turicensis	2.831197760862315e-7	1.4499611271637395e-6	-0.9331030098179081	0.3110000785084484	0.7335983792666142
Enterococcus durans	3.909296952036146e-6	0	2.295516434634877	0.31152507707860044	0.7335983792666142
Cardiobacterium hominis	0	6.441833783939607e-7	-0.7173712140717099	0.31152507707860044	0.7335983792666142
Segatella buccae	0	2.611783006799516e-7	-0.33477225292429297	0.31152507707860044	0.7335983792666142
Kingevirus communis	1.7430865233196462e-4	0	7.453753391624441	0.31368826616296425	0.7335983792666142
Weissella confusa	2.831197760862315e-7	0	0.3596558487597289	0.31368826616296425	0.7335983792666142
Lactobacillus iners	0	0	0	0.31536980210933613	0.7335983792666142
Streptococcus sp 1643	0	0	0	0.31536980210933613	0.7335983792666142
Phocaeicola abscessus	0	0	0	0.31536980210933613	0.7335983792666142
Prevotella nigrescens	0	0	0	0.31536980210933613	0.7335983792666142
Parolsenella catena	2.4639892107949765e-6	1.2815690212560952e-5	-1.9958012973211434	0.3203573923193902	0.7335983792666142
Lactobacillus helveticus	3.8110961589105143e-7	3.0764781595880664e-6	-1.5614954576159097	0.3203573923193902	0.7335983792666142
Mageeibacillus indolicus	7.354142796381178e-7	1.8352702824758002e-6	-0.7082061665279364	0.3203573923193902	0.7335983792666142
Bacteroides fluxus	1.1379039760090401e-6	1.8335981358485868e-5	-3.177019026381717	0.32440251017972743	0.7335983792666142
Ruminococcus albus	4.710340333330984e-7	3.1944056840971963e-6	-1.5116357813821837	0.32440251017972743	0.7335983792666142
Parolsenella massiliensis	2.3341460556938937e-6	5.954361728853645e-6	-1.060600805881234	0.32440251017972743	0.7335983792666142
Allocoprobacillus halotolerans	3.441514059674546e-6	1.699510902851211e-6	0.7183535145238259	0.32440251017972743	0.7335983792666142
Oribacterium parvum	9.270368250546164e-8	5.831215040224297e-7	-0.5348697595641322	0.3270730286802489	0.7335983792666142
Cohcovirus splanchnicus	6.589432346779246e-7	0	0.7302645213451511	0.3286688522735592	0.7335983792666142
Porphyromonas somerae	0	2.5275681862069594e-7	-0.3251063902228827	0.3286688522735592	0.7335983792666142
Duncaniella dubosii	0	1.3051490741272467e-7	-0.1769800161572092	0.3286688522735592	0.7335983792666142
Candida albicans	0	0	0	0.3348098486358626	0.7335983792666142
Desulfovibrio desulfuricans	0	0	0	0.3348098486358626	0.7335983792666142
Olsenella profusa	1.2015467485185337e-6	1.2327036586062754e-5	-2.5977666324219957	0.33548804137039434	0.7335983792666142
Lawsonibacter asaccharolyticus	2.1456325000846166e-4	8.332396447955889e-4	-1.952350630453665	0.33548804137039434	0.7335983792666142
Clostridium butyricum	7.087743989712991e-6	2.0427912515553313e-5	-1.4056820751856	0.33548804137039434	0.7335983792666142
Mogibacterium diversum	4.832866697310918e-5	1.24836966801359e-4	-1.351057594796588	0.33548804137039434	0.7335983792666142
Claveliimonas bilis	1.6339363288457064e-5	3.8141327246870376e-5	-1.1746417543449468	0.33548804137039434	0.7335983792666142
Roseburia sp 831b	2.410533520381505e-5	4.7809443306889114e-5	-0.9591663389653968	0.33548804137039434	0.7335983792666142
Christensenella minuta	2.8115655779162744e-5	5.18846893020679e-5	-0.8610549934232401	0.33548804137039434	0.7335983792666142
Streptococcus salivarius	0.0037656370115344427	0.004322647745330695	-0.19897198076994807	0.33548804137039434	0.7335983792666142
Oengusvirus oengus	8.493593282586945e-7	4.070291987836175e-6	-1.4550432656411683	0.34009737457712075	0.7335983792666142
Adlercreutzia mucosicola	3.4566224475721867e-6	1.353508576747047e-6	0.9211376345289303	0.34009737457712075	0.7335983792666142

Species	Median g1 (resp.)	Median g2 (non-resp.)	log2FC	p	padj
Methanobrevibacter smithii	0	3.3348930877783595e-4	-8.385816288773913	0.34182812897740666	0.7335983792666142
Alloprevotella tannerae	0	5.83269989876932e-7	-0.6629072945423433	0.34182812897740666	0.7335983792666142
Aggregatibacter segnis	0	0	0	0.3455570234313006	0.7335983792666142
Caproicibacter fermentans	0	0	0	0.3455570234313006	0.7335983792666142
Dehalococcoides mccartyi	0	0	0	0.3455570234313006	0.7335983792666142
Lactococcus piscium	0	0	0	0.3455570234313006	0.7335983792666142
Cellulosilyticum sp WCF-2	0	0	0	0.3455570234313006	0.7335983792666142
Fenollaria sporofastidiosus	0	0	0	0.3455570234313006	0.7335983792666142
Fructilactobacillus sanfranciscensis	0	0	0	0.3455570234313006	0.7335983792666142
Megasphaera stantonii	0	0	0	0.3455570234313006	0.7335983792666142
Selenomonas bovis	0	0	0	0.3455570234313006	0.7335983792666142
Enterococcus hirae	0	0	0	0.3455570234313006	0.7335983792666142
Anaerocolumna cellulosilytica	0	0	0	0.3455570234313006	0.7335983792666142
Bifidobacterium subtile	0	0	0	0.3455570234313006	0.7335983792666142
Streptococcus uberis	0	0	0	0.3455570234313006	0.7335983792666142
Corynebacterium kroppenstedtii	0	0	0	0.3455570234313006	0.7335983792666142
Enterococcus raffinosus	0	0	0	0.3455570234313006	0.7335983792666142
Enterococcus sp ZQ21	0	0	0	0.3455570234313006	0.7335983792666142
Haemophilus sp oral taxon 036	0	0	0	0.3455570234313006	0.7335983792666142
Lactobacillus phage phiPYB5	0	0	0	0.3455570234313006	0.7335983792666142
Leptogranulimonas caecicola	0	0	0	0.3455570234313006	0.7335983792666142
Leuconostoc kimchii	0	0	0	0.3455570234313006	0.7335983792666142
Metamycoplasma equirhinis	0	0	0	0.3455570234313006	0.7335983792666142
Neisseria macacae	0	0	0	0.3455570234313006	0.7335983792666142
Peptoniphilus mikwangii	0	0	0	0.3455570234313006	0.7335983792666142
Rothia amarae	0	0	0	0.3455570234313006	0.7335983792666142
Schleiferilactobacillus harbinensis	0	0	0	0.3455570234313006	0.7335983792666142
Akkermansia sp EB-AMDK43	0	0	0	0.3455570234313006	0.7335983792666142
Hymenobacter monticola	0	0	0	0.3455570234313006	0.7335983792666142
Kosakonia pseudosacchari	0	0	0	0.3455570234313006	0.7335983792666142
Lentilactobacillus buchneri	0	0	0	0.3455570234313006	0.7335983792666142
Peptostreptococcus sp CBA3647	0	0	0	0.3455570234313006	0.7335983792666142
Pseudomonas sp GCEP-101	0	0	0	0.3455570234313006	0.7335983792666142
Staphylococcus casei	0	0	0	0.3455570234313006	0.7335983792666142

Species	Median g1 (resp.)	Median g2 (non-resp.)	log2FC	p	padj
Streptococcus phage EJ-1	0	0	0	0.3455570234313006	0.7335983792666142
Streptomyces sp NEAU-S7GS2	0	0	0	0.3455570234313006	0.7335983792666142
Tetragenococcus koreensis	0	0	0	0.3455570234313006	0.7335983792666142
Variibacter gotjawalensis	0	0	0	0.3455570234313006	0.7335983792666142
Akkermansia sp RCC 12PD	0	0	0	0.3455570234313006	0.7335983792666142
Bacteroides graminisolvens	0	0	0	0.3455570234313006	0.7335983792666142
Blastocystis hominis	0	0	0	0.3455570234313006	0.7335983792666142
Colletotrichum coccodes	0	0	0	0.3455570234313006	0.7335983792666142
Corynebacterium aurimucosum	0	0	0	0.3455570234313006	0.7335983792666142
Corynebacterium heidelbergense	0	0	0	0.3455570234313006	0.7335983792666142
Latilactobacillus fuchuensis	0	0	0	0.3455570234313006	0.7335983792666142
Neisseria sicca	0	0	0	0.3455570234313006	0.7335983792666142
Occallatibacter riparius	0	0	0	0.3455570234313006	0.7335983792666142
Sporothrix globosa	0	0	0	0.3455570234313006	0.7335983792666142
Streptococcus ferus	0	0	0	0.3455570234313006	0.7335983792666142
Streptomyces nigrescens	0	0	0	0.3455570234313006	0.7335983792666142
Streptomyces sp NA02950	0	0	0	0.3455570234313006	0.7335983792666142
Treponema socranskii	0	0	0	0.3455570234313006	0.7335983792666142
Weissella paramesenteroides	0	0	0	0.3455570234313006	0.7335983792666142
Aurodevirus hiberniae	0	0	0	0.3455570234313006	0.7335983792666142
Capnocytophaga sputigena	0	0	0	0.3455570234313006	0.7335983792666142
Chlamydia psittaci	0	0	0	0.3455570234313006	0.7335983792666142
Cloacibacillus evryensis	0	0	0	0.3455570234313006	0.7335983792666142
Corynebacterium vitaeruminis	0	0	0	0.3455570234313006	0.7335983792666142
Enterococcus italicus	0	0	0	0.3455570234313006	0.7335983792666142
Gordonia bronchialis	0	0	0	0.3455570234313006	0.7335983792666142
Klebsiella huaxiensis	0	0	0	0.3455570234313006	0.7335983792666142
Lactobacillus equicursoris	0	0	0	0.3455570234313006	0.7335983792666142
Lentilactobacillus parafarraginis	0	0	0	0.3455570234313006	0.7335983792666142
Methanosphaera stadtmanae	0	0	0	0.3455570234313006	0.7335983792666142
Mycobacterium paragordonae	0	0	0	0.3455570234313006	0.7335983792666142
Pontibacter actiniarum	0	0	0	0.3455570234313006	0.7335983792666142
Porphyromonas crevioricanis	0	0	0	0.3455570234313006	0.7335983792666142
Prevotella scopos	0	0	0	0.3455570234313006	0.7335983792666142

Species	Median g1 (resp.)	Median g2 (non-resp.)	log2FC	p	padj
Prevotella sp E9-3	0	0	0	0.3455570234313006	0.7335983792666142
Propionibacterium australiense	0	0	0	0.3455570234313006	0.7335983792666142
Serinicoccus profundi	0	0	0	0.3455570234313006	0.7335983792666142
Streptococcus macacae	0	0	0	0.3455570234313006	0.7335983792666142
uncultured Desulfobulbus sp	0	0	0	0.3455570234313006	0.7335983792666142
Butyrivibrio proteoclasticus	0	0	0	0.3455570234313006	0.7335983792666142
Enterobacter cloacae	0	0	0	0.3455570234313006	0.7335983792666142
Hoylesella nanceiensis	0	0	0	0.3455570234313006	0.7335983792666142
Lacrimispora celerecrescens	0	0	0	0.3455570234313006	0.7335983792666142
Lactiplantibacillus paraplantarum	0	0	0	0.3455570234313006	0.7335983792666142
Leptotrichia sp oral taxon 221	0	0	0	0.3455570234313006	0.7335983792666142
Porphyromonas endodontalis	0	0	0	0.3455570234313006	0.7335983792666142
Prevotella sp oral taxon 299	0	0	0	0.3455570234313006	0.7335983792666142
Selenomonas dianae	0	0	0	0.3455570234313006	0.7335983792666142
Selenomonas sp oral taxon 478	0	0	0	0.3455570234313006	0.7335983792666142
Actinotignum schaalii	0	0	0	0.3455570234313006	0.7335983792666142
Agrobacterium vaccinii	0	0	0	0.3455570234313006	0.7335983792666142
Anaeropeptidivorans aminofermentans	0	0	0	0.3455570234313006	0.7335983792666142
Pannonibacter phragmitetus	0	0	0	0.3455570234313006	0.7335983792666142
Sulfitobacter geojensis	0	0	0	0.3455570234313006	0.7335983792666142
Sphaerochaeta associata	0	0	0	0.3455570234313006	0.7335983792666142
Akkermansia glycaniphila	0	0	0	0.3455570234313006	0.7335983792666142
Proteiniphilum propionicum	0	0	0	0.3455570234313006	0.7335983792666142
Corynebacterium otitidis	0	0	0	0.3455570234313006	0.7335983792666142
Prevotella herbatica	0	0	0	0.3455570234313006	0.7335983792666142
Acidipropionibacterium jensenii	0	0	0	0.3455570234313006	0.7335983792666142
Leptotrichia sp oral taxon 498	0	0	0	0.3455570234313006	0.7335983792666142
Actinomyces sp HMT 175	2.41075389095678e-7	9.247284931890888e-7	-0.6330641955354617	0.34647108115408987	0.7335983792666142
Campylobacter concisus	0	9.509187240109543e-7	-0.9641536759947203	0.35184053260698694	0.7335983792666142
Pyramidobacter piscolens	5.241951651819096e-7	2.125796110643436e-7	0.32996817463747097	0.35184053260698694	0.7335983792666142
Latilactobacillus sakei	1.132479104344926e-6	2.454609764832781e-6	-0.6959911469755311	0.35933256174351913	0.7335983792666142
Leuconostoc mesenteroides	1.0347666932696178e-6	0	1.0248633842905623	0.36520163627327457	0.7335983792666142
Hoylesella oralis	0	2.6102981482544935e-7	-0.334602386048627	0.36520163627327457	0.7335983792666142
Streptococcus cristatus	7.516469171833862e-7	5.212325944350191e-6	-1.8264215286651566	0.368608258455818	0.7335983792666142

Species	Median g1 (resp.)	Median g2 (non-resp.)	log2FC	p	padj
Leyella stercorea	5.358475937865802e-7	3.0255787851163695e-6	-1.3901611635948632	0.37111675299713726	0.7335983792666142
Mobiluncus sp Marseille-Q7826	2.7811104751638493e-7	0	0.35401318897758316	0.3721460515689588	0.7335983792666142
Enterobius vermicularis	2.2995864423742035e-7	0	0.2986098076322935	0.3721460515689588	0.7335983792666142
Leuconostoc gasicomitatum	0	0	0	0.3721460515689588	0.7335983792666142
Staphylococcus pasteuri	0	0	0	0.3721460515689588	0.7335983792666142
Lachnoanaerobaculum gingivalis	6.642293919772829e-7	9.793039043335085e-7	-0.25013883866939624	0.3726080838430366	0.7335983792666142
Slackia exigua	3.920454847806797e-5	5.92172162291291e-6	2.538155902039293	0.3733499217767347	0.7335983792666142
Bacteroides sp HF-162	1.8240864487414046e-5	5.60060361459887e-6	1.5435037527445288	0.3733499217767347	0.7335983792666142
Coprobacillus cateniformis	3.2986308945827825e-4	1.1667139918481522e-4	1.4914706495711785	0.3733499217767347	0.7335983792666142
Peptostreptococcus anaerobius	1.4219046751245145e-5	5.218688284573677e-6	1.2911957899590905	0.3733499217767347	0.7335983792666142
Intestinibacillus sp NTUH-41-i26	8.275790901017369e-5	3.9104889856720845e-5	1.0624472758462018	0.3733499217767347	0.7335983792666142
Prevotella falsenii	0	0	0	0.3740126751144955	0.7335983792666142
Paramuribaculum intestinale	0	0	0	0.3740126751144955	0.7335983792666142
Caproicibacter sp BJN0012	0	0	0	0.3740126751144955	0.7335983792666142
Mitsuokella jalaludinii	0	0	0	0.3740126751144955	0.7335983792666142
Mitsuokella multacida	0	0	0	0.3740126751144955	0.7335983792666142
Klebsiella pneumoniae	2.41075389095678e-7	4.281679677573662e-6	-2.089406053223556	0.37776390082678984	0.7335983792666142
Phoenicibacter congonensis	2.2995864423742035e-7	3.2415321040603763e-6	-1.785975674060616	0.37776390082678984	0.7335983792666142
Campylobacter gracilis	4.635184125273082e-7	1.1788546786552846e-6	-0.5741290809647898	0.37776390082678984	0.7335983792666142
Murdochiella vaginalis	2.41075389095678e-7	6.198623632621774e-7	-0.3842804805556139	0.37776390082678984	0.7335983792666142
Corynebacterium accolens	0	4.357268097477316e-7	-0.5217812591070929	0.38213038422617035	0.7335983792666142
Bifidobacterium longum	3.522804075621611e-4	0.022819429158193624	-6.013368371206833	0.3845076492135316	0.7335983792666142
Wujia chipingensis	1.384035449648464e-5	4.331414054402306e-5	-1.5782415785290225	0.3845076492135316	0.7335983792666142
Roseburia intestinalis	5.344394116712628e-4	0.0011635304465852413	-1.1209531198966975	0.3845076492135316	0.7335983792666142
Eubacterium ventriosum	5.803374380590943e-5	1.2424962788481976e-4	-1.085194574466851	0.3845076492135316	0.7335983792666142
Enterococcus faecalis	2.2665587601156027e-5	1.0318803106069042e-5	1.0640693336082703	0.3845076492135316	0.7335983792666142
Holdemanella biformis	2.772063684635538e-5	5.2078070119492025e-5	-0.88602817672418	0.3845076492135316	0.7335983792666142
Streptococcus infantis	3.51170176783194e-6	6.036769810753975e-6	-0.641241613878511	0.3845076492135316	0.7335983792666142
Anaerotruncus colihominis	3.3873494827190706e-4	2.2961354214721315e-4	0.558932400993127	0.3845076492135316	0.7335983792666142
[Ruminococcus] torques	0.012455122486740619	0.008826081073596826	0.4968466827409847	0.3845076492135316	0.7335983792666142
Streptococcus dysgalactiae	7.121094224287763e-7	0	0.7757749086027642	0.3950469150171537	0.7335983792666142
Gemella sanguinis	4.5725328141867e-6	3.1002454299979084e-5	-2.5217774390527206	0.39902572995276997	0.7335983792666142
Streptococcus mutans	8.809262288657861e-6	1.2056907832308552e-5	-0.412596727986833	0.39902572995276997	0.7335983792666142
Collinsella sp zg1085	0	0	0	0.4032253105267109	0.7335983792666142

Species	Median g1 (resp.)	Median g2 (non-resp.)	log2FC	p	padj
Pichia manshurica	0	0	0	0.4032253105267109	0.7335983792666142
Lactobacillus isalae	0	0	0	0.4032253105267109	0.7335983792666142
Prevotella corporis	0	1.435960953248976e-6	-1.2844910080367133	0.40483201676552083	0.7335983792666142
Paraclostridium bifermentans	3.538189447804882e-6	0	2.182116835917197	0.4107541169919414	0.7335983792666142
Turicibacter sanguinis	2.835904483386054e-5	4.657440402954355e-6	2.3755836454192147	0.421141170140234	0.7335983792666142
Streptococcus pasteurianus	4.622476779808436e-6	9.506217523019496e-7	1.527271747724753	0.421141170140234	0.7335983792666142
Raoultibacter timonensis	2.780335990192748e-6	1.3960092890551185e-6	0.6578809632616867	0.421141170140234	0.7335983792666142
Streptococcus sp D7B5	0	0	0	0.4243085065215573	0.7335983792666142
Chordicoccus furentiruminis	2.679237968932901e-6	1.0104479638279689e-5	-1.5936628927072054	0.4250178006822407	0.7335983792666142
Eubacterium sp MSJ-33	5.164372935071036e-6	1.875036202754428e-5	-1.67985304794696	0.42573622249538295	0.7335983792666142
Peptacetobacter hiranonis	1.9055480794552572e-7	0	0.2516340375180648	0.4348594842884019	0.7335983792666142
Porphyromonas gingivalis	0	0	0	0.4348594842884019	0.7335983792666142
Dialister hominis	0	0	0	0.4348594842884019	0.7335983792666142
Corynebacterium kefirresidentii	0	0	0	0.4348594842884019	0.7335983792666142
Phocaeicola paurosaccharolyticus	0	0	0	0.4348594842884019	0.7335983792666142
Faecalibacillus intestinalis	0.001672868581282285	1.5174882692010105e-4	3.453953057911632	0.43708252531781944	0.7335983792666142
Ruminococcus sp SR1/5	0.0012626244777612123	4.0499530497277046e-4	1.6380328400705975	0.43708252531781944	0.7335983792666142
Trichuris trichiura	1.2357473222787444e-5	3.995211328203497e-5	-1.616290777192659	0.43708252531781944	0.7335983792666142
Bifidobacterium catenulatum	4.657239541291207e-5	1.1858599359061204e-4	-1.329851848713842	0.43708252531781944	0.7335983792666142
Streptococcus australis	3.5546624254090945e-6	7.256635896827892e-6	-0.8582099554837234	0.43708252531781944	0.7335983792666142
Coprococcus comes	0.0064214160084402035	0.003554266355069834	0.8531584694825428	0.43708252531781944	0.7335983792666142
Dysosmobacter welbionis	2.3679413025027825e-4	4.0983800124486016e-4	-0.788856528859181	0.43708252531781944	0.7335983792666142
Faecalibacterium sp I4-3-84	3.313997711944038e-4	4.424094475546635e-4	-0.4157201204441755	0.43708252531781944	0.7335983792666142
Wansuia hejianensis	8.208445490402157e-4	6.158611644718492e-4	0.4139197137449422	0.43708252531781944	0.7335983792666142
Enterococcus cecorum	1.1197965367445631e-5	1.2117514739487832e-5	-0.10485388615335922	0.43708252531781944	0.7335983792666142
Streptococcus pyogenes	0	1.3051490741272467e-7	-0.1769800161572092	0.4406139868840486	0.7335983792666142
Clostridium paraputrificum	0	0	0	0.4410322960236078	0.7335983792666142
Klebsiella oxytoca	0	0	0	0.4410322960236078	0.7335983792666142
Lachnoanaerobaculum umeaense	0	0	0	0.4410322960236078	0.7335983792666142
Pediococcus acidilactici	0	0	0	0.4410322960236078	0.7335983792666142
Lentilactobacillus parabuchneri	0	0	0	0.4410322960236078	0.7335983792666142
Porphyromonas macacae	0	0	0	0.4410322960236078	0.7335983792666142
Streptococcus sobrinus	0	0	0	0.4410322960236078	0.7335983792666142
Cardiobacterium sp Marseille-Q4385	0	0	0	0.4410322960236078	0.7335983792666142

Species	Median g1 (resp.)	Median g2 (non-resp.)	log2FC	p	padj
Cutibacterium granulosum	0	0	0	0.4410322960236078	0.7335983792666142
Hoylesella shahii	0	0	0	0.4410322960236078	0.7335983792666142
Segatella oris	0	0	0	0.4410322960236078	0.7335983792666142
Enorma massiliensis	9.12645188797995e-6	6.346880845041597e-6	0.4629249886998357	0.45135075157155957	0.7335983792666142
Peptoanaerobacter stomatis	0	5.525905668366642e-7	-0.6346774272316917	0.45298880461588614	0.7335983792666142
Veillonella montpellierensis	0	4.400343517104501e-7	-0.5261032272586971	0.45298880461588614	0.7335983792666142
Barnesiella viscericola	8.165606290890208e-6	1.0038069377757485e-5	-0.2681856373524595	0.45346217410453693	0.7335983792666142
Hoylesella buccalis	7.652705542775875e-7	2.429161845453159e-6	-0.9579666812958557	0.4778265218869533	0.7335983792666142
Haemophilus influenzae	0	0	0	0.479393285813905	0.7335983792666142
Pediococcus pentosaceus	0	0	0	0.479393285813905	0.7335983792666142
Terrisporobacter glycolicus	0	0	0	0.479393285813905	0.7335983792666142
Thermophilibacter immobilis	0	0	0	0.479393285813905	0.7335983792666142
Citrobacter koseri	0	0	0	0.479393285813905	0.7335983792666142
Pseudobutyrivibrio xylanivorans	0	0	0	0.479393285813905	0.7335983792666142
Citrobacter portucalensis	0	0	0	0.479393285813905	0.7335983792666142
Culoivirus intestinalis	0	0	0	0.479393285813905	0.7335983792666142
Prevotella aurantiaca	0	0	0	0.479393285813905	0.7335983792666142
Treponema peruense	1.4081857211653737e-5	3.820449629954184e-6	1.645574470074229	0.4801307932863087	0.7335983792666142
Bulleidia extructa	2.742189614006868e-6	8.537213447797382e-7	1.0134582693674243	0.4801307932863087	0.7335983792666142
Bifidobacterium angulatum	1.6318596995849913e-6	2.61106604383481e-6	-0.45634222313906475	0.4801307932863087	0.7335983792666142
Limosilactobacillus fermentum	1.1725623946500302e-6	1.54773801986247e-6	-0.22981932407572767	0.4801307932863087	0.7335983792666142
Veillonella rogosae	4.685884069648005e-6	8.61608111293667e-6	-0.7580643814395208	0.4814989838197967	0.7335983792666142
Haemophilus parainfluenzae	4.19052715131721e-6	4.814546194102034e-5	-3.2431052335955677	0.4821790912834295	0.7335983792666142
Streptococcus sp NPS 308	0	0	0	0.488661290622056	0.7335983792666142
Streptococcus milleri	2.2995864423742035e-7	8.987686480184801e-7	-0.626454326033405	0.4927014026521794	0.7335983792666142
Lautropia mirabilis	9.270368250546164e-8	4.6066279633336255e-7	-0.41872093438736663	0.4927014026521794	0.7335983792666142
Solibaculum mannosilyticum	1.9837568627262775e-5	7.125306924399443e-5	-1.7938719226270354	0.49364361129067014	0.7335983792666142
Streptococcus sanguinis	1.9613598876153045e-5	6.909166943770681e-5	-1.7656465833243944	0.49364361129067014	0.7335983792666142
Actinomyces massiliensis	6.017112567524924e-5	1.4263431656629663e-4	-1.2314777435659898	0.49364361129067014	0.7335983792666142
Acutalibacter muris	4.460290934362699e-6	9.778334915697613e-6	-0.9810845937103434	0.49364361129067014	0.7335983792666142
Vescimonas fastidiosa	1.757389273494346e-4	3.466535610661594e-4	-0.9760305339670923	0.49364361129067014	0.7335983792666142
Bacteroides salyersiae	1.2143318299607061e-4	6.946695871616524e-5	0.7969757686011041	0.49364361129067014	0.7335983792666142
Coprococcus catus	0.0035320027083194693	0.0022067707356095718	0.6783044909179472	0.49364361129067014	0.7335983792666142
Anaerobutyricum hallii	0.03247463939082197	0.021259087550269166	0.6112103922134411	0.49364361129067014	0.7335983792666142

Species	Median g1 (resp.)	Median g2 (non-resp.)	log2FC	p	padj
Lacticaseibacillus paracasei	2.212674150803897e-5	3.3602764133507386e-5	-0.5813252789692394	0.49364361129067014	0.7335983792666142
Actinomyces johnsonii	1.7758209363499084e-5	2.2961318396719044e-5	-0.3531851736378377	0.49364361129067014	0.7335983792666142
Streptococcus vestibularis	5.581641561331481e-5	6.822827410379683e-5	-0.2850535626236347	0.49364361129067014	0.7335983792666142
Actinomyces israelii	2.7589064996583683e-5	3.061151315345025e-5	-0.1449866571159586	0.49364361129067014	0.7335983792666142
Bifidobacterium dentium	4.961936031764012e-4	5.258705310639252e-4	-0.0836407541153936	0.49364361129067014	0.7335983792666142
Canhaevirus hiberniae	9.198345769496814e-7	0	0.9409820061047992	0.5032750953764542	0.7335983792666142
Winkia neuii	2.527278177003487e-7	0	0.3250729919067554	0.5032750953764542	0.7335983792666142
Cellulomonas carbonis	1.9055480794552572e-7	0	0.2516340375180648	0.5032750953764542	0.7335983792666142
Streptococcus sp DTU 2020 1000888 1 SI GRL NUU 041A	4.710340333330984e-7	0	0.5568306246708563	0.5039191973059373	0.7335983792666142
Bacteroides pyogenes	0	2.4808318526901893e-7	-0.3197140936357452	0.5039191973059373	0.7335983792666142
Mogibacterium neglectum	1.771616497650853e-6	2.650186648772205e-6	-0.3972425878853748	0.5098833111244588	0.7335983792666142
Shouchella clausii	0	0	0	0.5144526269147639	0.7335983792666142
Gleimia europaea	0	0	0	0.5144526269147639	0.7335983792666142
Levilactobacillus brevis	0	0	0	0.5144526269147639	0.7335983792666142
Penicillium expansum	0	0	0	0.5144526269147639	0.7335983792666142
Bifidobacterium thermophilum	0	0	0	0.5144526269147639	0.7335983792666142
Enterococcus casseliflavus	0	0	0	0.5144526269147639	0.7335983792666142
Lactobacillus crispatus	0	0	0	0.5144526269147639	0.7335983792666142
Kandleria vitulina	0	0	0	0.5144526269147639	0.7335983792666142
Fusobacterium polymorphum	0	0	0	0.5144526269147639	0.7335983792666142
Enterococcus saigonensis	1.9323728364048246e-6	0	1.5520685465262622	0.5154450553605345	0.7335983792666142
Toxoplasma gondii	1.3178864693558492e-6	0	1.2128099045201917	0.5154450553605345	0.7335983792666142
Eggerthia catenaformis	1.1831434557618747e-6	0	1.1264069338634095	0.5154450553605345	0.7335983792666142
Emergencia timonensis	7.835666829170823e-7	0	0.8347651554264645	0.5154450553605345	0.7335983792666142
Lancefieldella sp Marseille-Q7238	6.53934506108078e-7	0	0.7259021065866595	0.5154450553605345	0.7335983792666142
Bifidobacterium animalis	1.9055480794552572e-7	8.57124245302102e-7	-0.6414363001489342	0.5154450553605345	0.7335983792666142
Faecalibaculum rodentium	5.526209709803023e-7	2.611783006799516e-7	0.29993342610713375	0.5241136158662147	0.7335983792666142
Pseudoramibacter alactolyticus	6.820980021543857e-6	1.0672919331346624e-6	1.9196072645968343	0.5305457666371869	0.7335983792666142
Parabacteroides faecis	1.1796198376497223e-5	2.3465541359835347e-6	1.9349670059512123	0.5352035608169541	0.7335983792666142
Phascolarctobacterium faecium	7.818180829143663e-5	1.8438700392218914e-6	4.799241572387023	0.5384475410657596	0.7335983792666142
Megasphaera micronuciformis	8.189673698728117e-7	4.721827123918283e-6	-1.6533562465634999	0.5384475410657596	0.7335983792666142
Parabacteroides chongii	1.3701908141177233e-5	4.274466959151401e-6	1.4789062060333003	0.5384475410657596	0.7335983792666142
Trueperella pyogenes	3.7081473002184654e-7	2.231954281556342e-6	-1.2373731907577137	0.5384475410657596	0.7335983792666142
Actinomyces sp HMT897	1.2980806279838517e-6	2.0692212699930076e-6	-0.41744324234297137	0.5384475410657596	0.7335983792666142

Species	Median g1 (resp.)	Median g2 (non-resp.)	log2FC	p	padj
Corynebacterium pyruviciproducens	1.242412603899097e-6	1.7337111967782977e-6	-0.2858090788742267	0.5384475410657596	0.7335983792666142
Collinsella tanakaei	2.029894098964903e-6	1.6443934329023999e-6	0.19633053240965911	0.5384475410657596	0.7335983792666142
Alistipes ihumii	7.722598463428716e-4	3.2263187108048875e-4	1.2565996085190472	0.5405730982285732	0.7335983792666142
Phocaeicola plebeius	1.2323781731632622e-4	2.6073273796498832e-5	2.1981631299115514	0.5418345194189556	0.7335983792666142
Mogibacterium timidum	1.3318860873839856e-5	6.178420322955419e-6	0.9961784188036387	0.5418345194189556	0.7335983792666142
Alistipes senegalensis	2.6077320264261623e-4	1.7583651029831732e-4	0.5659012487040843	0.5418345194189556	0.7335983792666142
Segatella copri	1.930222678865199e-5	1.322327393261271e-4	-2.714238761048557	0.5424613726475143	0.7335983792666142
Bacteroides caecimuris	9.058614053437123e-6	1.9900541792189105e-5	-1.0551088067364132	0.5424613726475143	0.7335983792666142
Amedibacillus dolichus	1.5286084187732573e-5	9.531020757854495e-6	0.6289944835313548	0.5424613726475143	0.7335983792666142
Blautia hydrogenotrophica	0.001162226451897558	1.9521891033647896e-4	2.567595990601656	0.5532212885154062	0.7335983792666142
Simiaoa sunii	2.6986983771189155e-4	8.763572339737549e-4	-1.6955646317093989	0.5532212885154062	0.7335983792666142
Massilistercora timonensis	2.9114188524235996e-5	7.278510714446999e-5	-1.2928862670616748	0.5532212885154062	0.7335983792666142
Corynebacterium durum	3.182939495823087e-5	7.877871343968181e-5	-1.2810156963690256	0.5532212885154062	0.7335983792666142
Streptococcus mitis	1.3211186423043268e-5	2.9163208592855586e-5	-1.0857628993498651	0.5532212885154062	0.7335983792666142
Bacteroides clarus	7.498938621839399e-5	3.619905483072469e-5	1.0305319596847924	0.5532212885154062	0.7335983792666142
Blautia parvula	1.0809753340533082e-5	4.923135618082911e-6	0.9955458087548497	0.5532212885154062	0.7335983792666142
Odoribacter splanchnicus	5.320335946194525e-4	0.00100664686260553	-0.918691756827528	0.5532212885154062	0.7335983792666142
Streptococcus suis	1.670162922644309e-5	3.140068352653806e-5	-0.8721420990994228	0.5532212885154062	0.7335983792666142
Roseburia rectibacter	6.748078871157418e-5	1.2405206030693621e-4	-0.868757604119667	0.5532212885154062	0.7335983792666142
Blautia sp SC05B48	4.5658775982373935e-4	2.857908766911927e-4	0.6740493313038417	0.5532212885154062	0.7335983792666142
Blautia pseudococcoides	4.557040775700274e-6	5.481856657717847e-6	-0.22209029035377148	0.5532212885154062	0.7335983792666142
Eubacterium ramulus	9.037608930329854e-4	0.0010524777892553087	-0.21955142035937755	0.5532212885154062	0.7335983792666142
Blautia producta	2.2876747198746536e-5	2.5510521784330713e-5	-0.15095875854469323	0.5532212885154062	0.7335983792666142
Aspergillus ruber	0	0	0	0.5556897902827946	0.7335983792666142
Bifidobacterium minimum	0	0	0	0.5556897902827946	0.7335983792666142
Malassezia restricta	0	0	0	0.5556897902827946	0.7335983792666142
Neisseria flavescens	0	0	0	0.5556897902827946	0.7335983792666142
Neisseria mucosa	0	0	0	0.5556897902827946	0.7335983792666142
Wickerhamomyces anomalus	0	0	0	0.5556897902827946	0.7335983792666142
Birpovirus hiberniae	0	0	0	0.5556897902827946	0.7335983792666142
Birpovirus hominis	0	0	0	0.5556897902827946	0.7335983792666142
Blohavirus americanus	0	0	0	0.5556897902827946	0.7335983792666142
Cytobacillus kochii	0	0	0	0.5556897902827946	0.7335983792666142
Ezakiella massiliensis	0	0	0	0.5556897902827946	0.7335983792666142

Species	Median g1 (resp.)	Median g2 (non-resp.)	log2FC	p	padj
Hafnia alvei	0	0	0	0.5556897902827946	0.7335983792666142
Hafnia paralvei	0	0	0	0.5556897902827946	0.7335983792666142
Kocuria rhizophila	0	0	0	0.5556897902827946	0.7335983792666142
Methanobrevibacter sp YE315	0	0	0	0.5556897902827946	0.7335983792666142
Mobiluncus holmesii	0	0	0	0.5556897902827946	0.7335983792666142
Occultella kanbiaonis	0	0	0	0.5556897902827946	0.7335983792666142
Peptoniphilus obesi	0	0	0	0.5556897902827946	0.7335983792666142
Porphyromonas levii	0	0	0	0.5556897902827946	0.7335983792666142
Staphylococcus carnosus	0	0	0	0.5556897902827946	0.7335983792666142
Stenotrophomonas maltophilia	0	0	0	0.5556897902827946	0.7335983792666142
Sutterella faecalis	0	0	0	0.5556897902827946	0.7335983792666142
Treponema ruminis	0	0	0	0.5556897902827946	0.7335983792666142
Trueperella abortisuis	0	0	0	0.5556897902827946	0.7335983792666142
Anaerotignum propionicum	0	0	0	0.5556897902827946	0.7335983792666142
Bacillus cereus	0	0	0	0.5556897902827946	0.7335983792666142
Brucella intermedia	0	0	0	0.5556897902827946	0.7335983792666142
Clostridium felsineum	0	0	0	0.5556897902827946	0.7335983792666142
Corynebacterium glucuronolyticum	0	0	0	0.5556897902827946	0.7335983792666142
Duganella dendranthematis	0	0	0	0.5556897902827946	0.7335983792666142
Ndongobacter massiliensis	0	0	0	0.5556897902827946	0.7335983792666142
Anaerocolumna sedimenticola	0	0	0	0.5556897902827946	0.7335983792666142
Clostridiisalibacter paucivorans	0	0	0	0.5556897902827946	0.7335983792666142
Jahgtovirus intestinalis	0	0	0	0.5556897902827946	0.7335983792666142
Paludicola sp MB14-C6	0	0	0	0.5556897902827946	0.7335983792666142
Rodentolepis nana	0	0	0	0.5556897902827946	0.7335983792666142
Sphaerobacter thermophilus	0	0	0	0.5556897902827946	0.7335983792666142
Agromyces marinus	0	0	0	0.5556897902827946	0.7335983792666142
Anaerocolumna sp MB42-C2	0	0	0	0.5556897902827946	0.7335983792666142
Brussowvirus bv858	0	0	0	0.5556897902827946	0.7335983792666142
Clostridium sp JS66	0	0	0	0.5556897902827946	0.7335983792666142
Corynebacterium suedekumii	0	0	0	0.5556897902827946	0.7335983792666142
Enterococcus sulfureus	0	0	0	0.5556897902827946	0.7335983792666142
Gordonia sputi	0	0	0	0.5556897902827946	0.7335983792666142
Janibacter hoylei	0	0	0	0.5556897902827946	0.7335983792666142

Species	Median g1 (resp.)	Median g2 (non-resp.)	log2FC	p	padj
Kroppenstedtia pulmonis	0	0	0	0.5556897902827946	0.7335983792666142
Ligilactobacillus acidipiscis	0	0	0	0.5556897902827946	0.7335983792666142
Ligilactobacillus saerimneri	0	0	0	0.5556897902827946	0.7335983792666142
Limosilactobacillus gastricus	0	0	0	0.5556897902827946	0.7335983792666142
Liquorilactobacillus nagelii	0	0	0	0.5556897902827946	0.7335983792666142
Listeria innocua	0	0	0	0.5556897902827946	0.7335983792666142
Loigolactobacillus backii	0	0	0	0.5556897902827946	0.7335983792666142
Mammaliicoccus sciuri	0	0	0	0.5556897902827946	0.7335983792666142
Mycolicibacterium psychrotolerans	0	0	0	0.5556897902827946	0.7335983792666142
Neobacillus bataviensis	0	0	0	0.5556897902827946	0.7335983792666142
Paracoccus liaowanqingii	0	0	0	0.5556897902827946	0.7335983792666142
Polynucleobacter sp AP-Jannik-300A-C4	0	0	0	0.5556897902827946	0.7335983792666142
Rickettsiales endosymbiont of Stachyamoeba lipophora	0	0	0	0.5556897902827946	0.7335983792666142
Sedimentibacter sp MB35-C1	0	0	0	0.5556897902827946	0.7335983792666142
Selenobaculum gbiensis	0	0	0	0.5556897902827946	0.7335983792666142
Streptococcus henryi	0	0	0	0.5556897902827946	0.7335983792666142
Streptococcus ruminantium	0	0	0	0.5556897902827946	0.7335983792666142
Talaromyces marneffei	0	0	0	0.5556897902827946	0.7335983792666142
Acetivibrio saccincola	0	0	0	0.5556897902827946	0.7335983792666142
Amycolatopsis sp NBC 00355	0	0	0	0.5556897902827946	0.7335983792666142
Aquibium oceanicum	0	0	0	0.5556897902827946	0.7335983792666142
Aspergillus glaucus	0	0	0	0.5556897902827946	0.7335983792666142
Aspergillus pseudoglaucus	0	0	0	0.5556897902827946	0.7335983792666142
Bacillus sp NSP9 1	0	0	0	0.5556897902827946	0.7335983792666142
Bacillus sp S3	0	0	0	0.5556897902827946	0.7335983792666142
Bdellovibrio bacteriovorus	0	0	0	0.5556897902827946	0.7335983792666142
Brevibacterium casei	0	0	0	0.5556897902827946	0.7335983792666142
Brevibacterium zhoupengii	0	0	0	0.5556897902827946	0.7335983792666142
Brucella pseudintermedia	0	0	0	0.5556897902827946	0.7335983792666142
Brucella rhizosphaerae	0	0	0	0.5556897902827946	0.7335983792666142
Caballeronia grimmiae	0	0	0	0.5556897902827946	0.7335983792666142
Candidatus Protofrankia datiscae	0	0	0	0.5556897902827946	0.7335983792666142
Capnocytophaga leadbetteri	0	0	0	0.5556897902827946	0.7335983792666142
Capnocytophaga ochracea	0	0	0	0.5556897902827946	0.7335983792666142

Species	Median g1 (resp.)	Median g2 (non-resp.)	log2FC	p	padj
Chlamydiifrater phoenicopteri	0	0	0	0.5556897902827946	0.7335983792666142
Citrobacter pasteurii	0	0	0	0.5556897902827946	0.7335983792666142
Clostridium baratii	0	0	0	0.5556897902827946	0.7335983792666142
Clostridium formicaceticum	0	0	0	0.5556897902827946	0.7335983792666142
Clostridium gelidum	0	0	0	0.5556897902827946	0.7335983792666142
Clostridium lundense	0	0	0	0.5556897902827946	0.7335983792666142
Clostridium pasteurianum	0	0	0	0.5556897902827946	0.7335983792666142
Clostridium thermobutyricum	0	0	0	0.5556897902827946	0.7335983792666142
Corynebacterium sphenisci	0	0	0	0.5556897902827946	0.7335983792666142
Dechloromonas agitata	0	0	0	0.5556897902827946	0.7335983792666142
Deinococcus radiopugnans	0	0	0	0.5556897902827946	0.7335983792666142
Delmidovirus intestinihominis	0	0	0	0.5556897902827946	0.7335983792666142
Delmidovirus splanchnicus	0	0	0	0.5556897902827946	0.7335983792666142
Devosia ginsengisoli	0	0	0	0.5556897902827946	0.7335983792666142
Dickeya dadantii	0	0	0	0.5556897902827946	0.7335983792666142
Enterobacter cloacae complex sp ECL414	0	0	0	0.5556897902827946	0.7335983792666142
Enterobacter huaxiensis	0	0	0	0.5556897902827946	0.7335983792666142
Enterobacter sp E20	0	0	0	0.5556897902827946	0.7335983792666142
Frigidibacter mobilis	0	0	0	0.5556897902827946	0.7335983792666142
Haloarcula pelagica	0	0	0	0.5556897902827946	0.7335983792666142
Hoylesella loescheii	0	0	0	0.5556897902827946	0.7335983792666142
Humibacter albus	0	0	0	0.5556897902827946	0.7335983792666142
Hymenobacter cellulosilyticus	0	0	0	0.5556897902827946	0.7335983792666142
Lentilactobacillus kisonensis	0	0	0	0.5556897902827946	0.7335983792666142
Ligilactobacillus ceti	0	0	0	0.5556897902827946	0.7335983792666142
Lysinibacillus sp G01H	0	0	0	0.5556897902827946	0.7335983792666142
Mammaliicoccus lentus	0	0	0	0.5556897902827946	0.7335983792666142
Methylobacterium terrae	0	0	0	0.5556897902827946	0.7335983792666142
Micrococcus lylae	0	0	0	0.5556897902827946	0.7335983792666142
Micromonospora inositola	0	0	0	0.5556897902827946	0.7335983792666142
Nectriaceae sp PG4201	0	0	0	0.5556897902827946	0.7335983792666142
Nitrosopumilus piranensis	0	0	0	0.5556897902827946	0.7335983792666142
Paraburkholderia dokdonensis	0	0	0	0.5556897902827946	0.7335983792666142
Paraburkholderia phymatum	0	0	0	0.5556897902827946	0.7335983792666142

Species	Median g1 (resp.)	Median g2 (non-resp.)	log2FC	p	padj
Phanerodontia chrysosporium	0	0	0	0.5556897902827946	0.7335983792666142
Polyangium aurulentum	0	0	0	0.5556897902827946	0.7335983792666142
Priestia flexa	0	0	0	0.5556897902827946	0.7335983792666142
Pseudomonas siliginis	0	0	0	0.5556897902827946	0.7335983792666142
Rhizobium binae	0	0	0	0.5556897902827946	0.7335983792666142
Rhizobium jaguaris	0	0	0	0.5556897902827946	0.7335983792666142
Rhodococcus qingshengii	0	0	0	0.5556897902827946	0.7335983792666142
Rothia terrae	0	0	0	0.5556897902827946	0.7335983792666142
Ruania alkalisoli	0	0	0	0.5556897902827946	0.7335983792666142
Saccharopolyspora gregorii	0	0	0	0.5556897902827946	0.7335983792666142
Sandaracinus amylolyticus	0	0	0	0.5556897902827946	0.7335983792666142
Sedimentibacter sp zth1	0	0	0	0.5556897902827946	0.7335983792666142
Sphingomonas sp QA11	0	0	0	0.5556897902827946	0.7335983792666142
Staphylococcus hominis	0	0	0	0.5556897902827946	0.7335983792666142
Stenotrophomonas sp ESTM1D MKCIP4 1	0	0	0	0.5556897902827946	0.7335983792666142
Streptococcus caballi	0	0	0	0.5556897902827946	0.7335983792666142
Streptococcus phage CHPC1148	0	0	0	0.5556897902827946	0.7335983792666142
Streptomyces exfoliatus	0	0	0	0.5556897902827946	0.7335983792666142
Streptomyces sp P3	0	0	0	0.5556897902827946	0.7335983792666142
Sulfurovum sp XGS-02	0	0	0	0.5556897902827946	0.7335983792666142
Symbiobacterium thermophilum	0	0	0	0.5556897902827946	0.7335983792666142
Symmachiella dynata	0	0	0	0.5556897902827946	0.7335983792666142
Synechococcus sp BMK-MC-1	0	0	0	0.5556897902827946	0.7335983792666142
Synechococcus sp MEDNS5	0	0	0	0.5556897902827946	0.7335983792666142
Trichlorobacter lovleyi	0	0	0	0.5556897902827946	0.7335983792666142
anthomonas translucens	0	0	0	0.5556897902827946	0.7335983792666142
Acidomonas methanolica	0	0	0	0.5556897902827946	0.7335983792666142
Azoarcus olearius	0	0	0	0.5556897902827946	0.7335983792666142
Bohxovirus oralis	0	0	0	0.5556897902827946	0.7335983792666142
Candida parapsilosis	0	0	0	0.5556897902827946	0.7335983792666142
Candidatus Formimonas warabiya	0	0	0	0.5556897902827946	0.7335983792666142
Candidatus Nucleicultrix amoebiphila	0	0	0	0.5556897902827946	0.7335983792666142
Ceduovirus CHPC1020	0	0	0	0.5556897902827946	0.7335983792666142
Ceduovirus CHPC1170	0	0	0	0.5556897902827946	0.7335983792666142

Species	Median g1 (resp.)	Median g2 (non-resp.)	log2FC	p	padj
Ceduovirus cv05802	0	0	0	0.5556897902827946	0.7335983792666142
Cequinquevirus Ld25A	0	0	0	0.5556897902827946	0.7335983792666142
Clostridium intestinale	0	0	0	0.5556897902827946	0.7335983792666142
Corynebacterium minutissimum	0	0	0	0.5556897902827946	0.7335983792666142
Corynebacterium sp FDAARGOS 1242	0	0	0	0.5556897902827946	0.7335983792666142
Cryobacterium soli	0	0	0	0.5556897902827946	0.7335983792666142
Cysteiniphilum sp QT6929	0	0	0	0.5556897902827946	0.7335983792666142
Deinococcus psychrotolerans	0	0	0	0.5556897902827946	0.7335983792666142
Desulfovibrio mangrovi	0	0	0	0.5556897902827946	0.7335983792666142
Desulfuromonas sp AOP6	0	0	0	0.5556897902827946	0.7335983792666142
Dyella thiooxydans	0	0	0	0.5556897902827946	0.7335983792666142
Fictibacillus phosphorivorans	0	0	0	0.5556897902827946	0.7335983792666142
Fusarium proliferatum	0	0	0	0.5556897902827946	0.7335983792666142
Gemmatirosa kalamazoonensis	0	0	0	0.5556897902827946	0.7335983792666142
Geobacter sulfurreducens	0	0	0	0.5556897902827946	0.7335983792666142
Geotalea daltonii	0	0	0	0.5556897902827946	0.7335983792666142
Halomicrococcus gelatinilyticus	0	0	0	0.5556897902827946	0.7335983792666142
Ilumatobacter coccineus	0	0	0	0.5556897902827946	0.7335983792666142
Kitasatospora sp CM 4170	0	0	0	0.5556897902827946	0.7335983792666142
Kocuria palustris	0	0	0	0.5556897902827946	0.7335983792666142
Kosakonia oryzae	0	0	0	0.5556897902827946	0.7335983792666142
Kribbella sp NBC 00359	0	0	0	0.5556897902827946	0.7335983792666142
Ktedonosporobacter rubrisoli	0	0	0	0.5556897902827946	0.7335983792666142
Larkinella insperata	0	0	0	0.5556897902827946	0.7335983792666142
Leifsonia aquatica	0	0	0	0.5556897902827946	0.7335983792666142
Lysinibacillus sphaericus	0	0	0	0.5556897902827946	0.7335983792666142
Maritalea myrionectae	0	0	0	0.5556897902827946	0.7335983792666142
Methanomethylovorans hollandica	0	0	0	0.5556897902827946	0.7335983792666142
Microbacterium protaetiae	0	0	0	0.5556897902827946	0.7335983792666142
Microcella alkaliphila	0	0	0	0.5556897902827946	0.7335983792666142
Micromonospora sp NBC 01740	0	0	0	0.5556897902827946	0.7335983792666142
Mortierella polycephala	0	0	0	0.5556897902827946	0.7335983792666142
Mucilaginibacter sp KACC 22063	0	0	0	0.5556897902827946	0.7335983792666142
Mycobacteroides chelonae	0	0	0	0.5556897902827946	0.7335983792666142

Species	Median g1 (resp.)	Median g2 (non-resp.)	log2FC	p	padj
Myxococcus hansupus	0	0	0	0.5556897902827946	0.7335983792666142
Paenibacillus guangzhouensis	0	0	0	0.5556897902827946	0.7335983792666142
Pandoraea pnomenusa	0	0	0	0.5556897902827946	0.7335983792666142
Penicillium chrysogenum	0	0	0	0.5556897902827946	0.7335983792666142
Penicillium decumbens	0	0	0	0.5556897902827946	0.7335983792666142
Penicillium paneum	0	0	0	0.5556897902827946	0.7335983792666142
Plantibacter flavus	0	0	0	0.5556897902827946	0.7335983792666142
Profundibacter amoris	0	0	0	0.5556897902827946	0.7335983792666142
Proteiniborus sp MB09-C3	0	0	0	0.5556897902827946	0.7335983792666142
Pseudarthrobacter chlorophenolicus	0	0	0	0.5556897902827946	0.7335983792666142
Pseudomonas sp 905 Psudmo1	0	0	0	0.5556897902827946	0.7335983792666142
Rhodanobacter thiooxydans	0	0	0	0.5556897902827946	0.7335983792666142
Rhodobacter xanthinilyticus	0	0	0	0.5556897902827946	0.7335983792666142
Rhodococcus globerulus	0	0	0	0.5556897902827946	0.7335983792666142
Sphingobium sp TKS	0	0	0	0.5556897902827946	0.7335983792666142
Spirillospora sp NBC 00431	0	0	0	0.5556897902827946	0.7335983792666142
Stachybotrys chartarum	0	0	0	0.5556897902827946	0.7335983792666142
Staphylococcus capitis	0	0	0	0.5556897902827946	0.7335983792666142
Streptomyces lunaelactis	0	0	0	0.5556897902827946	0.7335983792666142
Streptomyces nojiriensis	0	0	0	0.5556897902827946	0.7335983792666142
Streptomyces sp NBC 01304	0	0	0	0.5556897902827946	0.7335983792666142
Streptomyces sp WZ-12	0	0	0	0.5556897902827946	0.7335983792666142
Tepidiforma bonchosmolovskayae	0	0	0	0.5556897902827946	0.7335983792666142
Thermoactinomyces daqus	0	0	0	0.5556897902827946	0.7335983792666142
Thermodesulfovibrio yellowstonii	0	0	0	0.5556897902827946	0.7335983792666142
Thioclava nitratireducens	0	0	0	0.5556897902827946	0.7335983792666142
Trichophyton simii	0	0	0	0.5556897902827946	0.7335983792666142
Tumebacillus algifaecis	0	0	0	0.5556897902827946	0.7335983792666142
Aeromonas diversa	0	0	0	0.5556897902827946	0.7335983792666142
Aeromonas hydrophila	0	0	0	0.5556897902827946	0.7335983792666142
Bacillus amyloliquefaciens	0	0	0	0.5556897902827946	0.7335983792666142
Bradyrhizobium erythrophlei	0	0	0	0.5556897902827946	0.7335983792666142
Brevundimonas sp GW460-12-10-14-LB2	0	0	0	0.5556897902827946	0.7335983792666142
Buorbuivirus hominis	0	0	0	0.5556897902827946	0.7335983792666142

Species	Median g1 (resp.)	Median g2 (non-resp.)	log2FC	p	padj
Caballeronia sordidicola	0	0	0	0.5556897902827946	0.7335983792666142
Carnobacterium mobile	0	0	0	0.5556897902827946	0.7335983792666142
Cellulomonas sp S1-8	0	0	0	0.5556897902827946	0.7335983792666142
Chitinophaga oryzae	0	0	0	0.5556897902827946	0.7335983792666142
Chlamydia avium	0	0	0	0.5556897902827946	0.7335983792666142
Chuhaivirus simiae	0	0	0	0.5556897902827946	0.7335983792666142
Citrobacter sp RHBSTW-00986	0	0	0	0.5556897902827946	0.7335983792666142
Clostridium sp LQ25	0	0	0	0.5556897902827946	0.7335983792666142
Cutibacterium modestum	0	0	0	0.5556897902827946	0.7335983792666142
Cytobacillus solani	0	0	0	0.5556897902827946	0.7335983792666142
Dactylosporangium fulvum	0	0	0	0.5556897902827946	0.7335983792666142
Dyella sp GSA-30	0	0	0	0.5556897902827946	0.7335983792666142
Flavobacterium branchiophilum	0	0	0	0.5556897902827946	0.7335983792666142
Hahella sp HNIBRBA332	0	0	0	0.5556897902827946	0.7335983792666142
Halocatena marina	0	0	0	0.5556897902827946	0.7335983792666142
Herbidospora cretacea	0	0	0	0.5556897902827946	0.7335983792666142
Hymenobacter swuensis	0	0	0	0.5556897902827946	0.7335983792666142
Hymenobacter volaticus	0	0	0	0.5556897902827946	0.7335983792666142
Jejuia pallidilutea	0	0	0	0.5556897902827946	0.7335983792666142
Lactobacillus phage LL-H	0	0	0	0.5556897902827946	0.7335983792666142
Lentilactobacillus hilgardii	0	0	0	0.5556897902827946	0.7335983792666142
Leptolyngbya sp 7M	0	0	0	0.5556897902827946	0.7335983792666142
Limnochorda sp LNt	0	0	0	0.5556897902827946	0.7335983792666142
Limosilactobacillus panis	0	0	0	0.5556897902827946	0.7335983792666142
Methylomusa anaerophila	0	0	0	0.5556897902827946	0.7335983792666142
Mumia sp ZJ1417	0	0	0	0.5556897902827946	0.7335983792666142
Mycobacterium intracellulare	0	0	0	0.5556897902827946	0.7335983792666142
Neobacillus sp DY30	0	0	0	0.5556897902827946	0.7335983792666142
Nocardioides sp BP30	0	0	0	0.5556897902827946	0.7335983792666142
Nocardiopsis xinjiangensis	0	0	0	0.5556897902827946	0.7335983792666142
Oceanithermus profundus	0	0	0	0.5556897902827946	0.7335983792666142
Paenibacillus larvae	0	0	0	0.5556897902827946	0.7335983792666142
Prevotella sp E15-22	0	0	0	0.5556897902827946	0.7335983792666142
Prevotella sp E2-28	0	0	0	0.5556897902827946	0.7335983792666142

Species	Median g1 (resp.)	Median g2 (non-resp.)	log2FC	p	padj
Proteiniclasticum ruminis	0	0	0	0.5556897902827946	0.7335983792666142
Pseudorhizobium flavum	0	0	0	0.5556897902827946	0.7335983792666142
Roseimaritima multifibrata	0	0	0	0.5556897902827946	0.7335983792666142
Roseivivax sp THAF30	0	0	0	0.5556897902827946	0.7335983792666142
Schistosoma mansoni	0	0	0	0.5556897902827946	0.7335983792666142
Schleiferilactobacillus shenzhenensis	0	0	0	0.5556897902827946	0.7335983792666142
Siminovitchia fordii	0	0	0	0.5556897902827946	0.7335983792666142
Solibacillus isronensis	0	0	0	0.5556897902827946	0.7335983792666142
Stenotrophomonas sp WZN-1	0	0	0	0.5556897902827946	0.7335983792666142
Streptococcus sp 29887	0	0	0	0.5556897902827946	0.7335983792666142
Streptomyces platensis	0	0	0	0.5556897902827946	0.7335983792666142
Streptomyces sp NBC 00234	0	0	0	0.5556897902827946	0.7335983792666142
Streptomyces sp NBC 00455	0	0	0	0.5556897902827946	0.7335983792666142
Synechococcus sp ROS8604	0	0	0	0.5556897902827946	0.7335983792666142
Treponema saccharophilum	0	0	0	0.5556897902827946	0.7335983792666142
Treponema sp OMZ 857	0	0	0	0.5556897902827946	0.7335983792666142
enorhabdus szentirmaii	0	0	0	0.5556897902827946	0.7335983792666142
ylanibacter brevis	0	0	0	0.5556897902827946	0.7335983792666142
uncultured Sphingopyxis sp	0	0	0	0.5556897902827946	0.7335983792666142
Akkermansia sp N21116	0	0	0	0.5556897902827946	0.7335983792666142
Cytophaga aurantiaca	0	0	0	0.5556897902827946	0.7335983792666142
Metarhizium anisopliae	0	0	0	0.5556897902827946	0.7335983792666142
Aggregatibacter sp Marseille-P9115	0	0	0	0.5556897902827946	0.7335983792666142
Eikenella exigua	0	0	0	0.5556897902827946	0.7335983792666142
Moorena producens	0	0	0	0.5556897902827946	0.7335983792666142
Nitratidesulfovibrio liaohensis	0	0	0	0.5556897902827946	0.7335983792666142
Paenibacillus barengoltzii	0	0	0	0.5556897902827946	0.7335983792666142
Porphyromonas catoniae	0	0	0	0.5556897902827946	0.7335983792666142
Treponema pedis	0	0	0	0.5556897902827946	0.7335983792666142
Acidithiobacillus sp AMEEHan	0	0	0	0.5556897902827946	0.7335983792666142
Actinobacillus suis	0	0	0	0.5556897902827946	0.7335983792666142
Alloactinosynnema sp L-07	0	0	0	0.5556897902827946	0.7335983792666142
Aspergillus fumigatus	0	0	0	0.5556897902827946	0.7335983792666142
Bifidobacterium porcinum	0	0	0	0.5556897902827946	0.7335983792666142

Species	Median g1 (resp.)	Median g2 (non-resp.)	log2FC	p	padj
Chitinophaga horti	0	0	0	0.5556897902827946	0.7335983792666142
Chondromyces apiculatus	0	0	0	0.5556897902827946	0.7335983792666142
Kingella oralis	0	0	0	0.5556897902827946	0.7335983792666142
Lactobacillus hamsteri	0	0	0	0.5556897902827946	0.7335983792666142
Lactobacillus pasteurii	0	0	0	0.5556897902827946	0.7335983792666142
Legionella adelaidensis	0	0	0	0.5556897902827946	0.7335983792666142
Leptotrichia buccalis	0	0	0	0.5556897902827946	0.7335983792666142
Leptotrichia sp oral taxon 218	0	0	0	0.5556897902827946	0.7335983792666142
Ligilactobacillus animalis	0	0	0	0.5556897902827946	0.7335983792666142
Loigolactobacillus bifermentans	0	0	0	0.5556897902827946	0.7335983792666142
Microvirgula aerodenitrificans	0	0	0	0.5556897902827946	0.7335983792666142
Ottowia sp oral taxon 894	0	0	0	0.5556897902827946	0.7335983792666142
Paracidovorax avenae	0	0	0	0.5556897902827946	0.7335983792666142
Ruania halotolerans	0	0	0	0.5556897902827946	0.7335983792666142
Secundilactobacillus paracollinoides	0	0	0	0.5556897902827946	0.7335983792666142
Acidihalobacter aeolianus	0	0	0	0.5556897902827946	0.7335983792666142
Actinacidiphila bryophytorum	0	0	0	0.5556897902827946	0.7335983792666142
Arthrobacter caoxuetaonis	0	0	0	0.5556897902827946	0.7335983792666142
Bacillus paranthracis	0	0	0	0.5556897902827946	0.7335983792666142
Brochothrix campestris	0	0	0	0.5556897902827946	0.7335983792666142
Corynebacterium ulcerans	0	0	0	0.5556897902827946	0.7335983792666142
Curtobacterium sp MR MD2014	0	0	0	0.5556897902827946	0.7335983792666142
Fervidobacterium pennivorans	0	0	0	0.5556897902827946	0.7335983792666142
Halobacillus amylolyticus	0	0	0	0.5556897902827946	0.7335983792666142
Helicobacter canadensis	0	0	0	0.5556897902827946	0.7335983792666142
Hymenobacter oligotrophus	0	0	0	0.5556897902827946	0.7335983792666142
Kolpuevirus coli	0	0	0	0.5556897902827946	0.7335983792666142
Kolpuevirus hominis	0	0	0	0.5556897902827946	0.7335983792666142
Leptospira kirschneri	0	0	0	0.5556897902827946	0.7335983792666142
Mahstovirus faecalis	0	0	0	0.5556897902827946	0.7335983792666142
Methylobacterium sp WL1	0	0	0	0.5556897902827946	0.7335983792666142
Mycobacterium noviomagense	0	0	0	0.5556897902827946	0.7335983792666142
Paenibacillus sp PK3 47	0	0	0	0.5556897902827946	0.7335983792666142
Paracoccus pantotrophus	0	0	0	0.5556897902827946	0.7335983792666142

Species	Median g1 (resp.)	Median g2 (non-resp.)	log2FC	p	padj
Paroceanicella profunda	0	0	0	0.5556897902827946	0.7335983792666142
Phaeobacter gallaeciensis	0	0	0	0.5556897902827946	0.7335983792666142
Photobacterium sp CCB-ST2H9	0	0	0	0.5556897902827946	0.7335983792666142
Pragia fontium	0	0	0	0.5556897902827946	0.7335983792666142
Ralstonia pickettii	0	0	0	0.5556897902827946	0.7335983792666142
Rhodococcus erythropolis	0	0	0	0.5556897902827946	0.7335983792666142
Sphingomonas profundi	0	0	0	0.5556897902827946	0.7335983792666142
Staphylococcus caprae	0	0	0	0.5556897902827946	0.7335983792666142
Staphylococcus kloosii	0	0	0	0.5556897902827946	0.7335983792666142
Streptomyces sp NBC 01343	0	0	0	0.5556897902827946	0.7335983792666142
Streptomyces zinciresistens	0	0	0	0.5556897902827946	0.7335983792666142
Sulfuriflexus mobilis	0	0	0	0.5556897902827946	0.7335983792666142
Synoicihabitans lomoniglobus	0	0	0	0.5556897902827946	0.7335983792666142
Thalassovita sp	0	0	0	0.5556897902827946	0.7335983792666142
Thiomicrospira pelophila	0	0	0	0.5556897902827946	0.7335983792666142
Vibrio zhugei	0	0	0	0.5556897902827946	0.7335983792666142
Vulgatibacter incomptus	0	0	0	0.5556897902827946	0.7335983792666142
[Candida] silvatica	0	0	0	0.5556897902827946	0.7335983792666142
Actinokineospora enzanensis	0	0	0	0.5556897902827946	0.7335983792666142
Actinomyces capricornis	0	0	0	0.5556897902827946	0.7335983792666142
Aliarcobacter butzleri	0	0	0	0.5556897902827946	0.7335983792666142
Alysiella filiformis	0	0	0	0.5556897902827946	0.7335983792666142
Anaeromusa acidaminophila	0	0	0	0.5556897902827946	0.7335983792666142
Anatilimnocola aggregata	0	0	0	0.5556897902827946	0.7335983792666142
Aquitalea magnusonii	0	0	0	0.5556897902827946	0.7335983792666142
Atlantibacter hermannii	0	0	0	0.5556897902827946	0.7335983792666142
Bordetella sp H567	0	0	0	0.5556897902827946	0.7335983792666142
Burkholderia sp THE68	0	0	0	0.5556897902827946	0.7335983792666142
Caminicella sporogenes	0	0	0	0.5556897902827946	0.7335983792666142
Carvajevirus carvaje	0	0	0	0.5556897902827946	0.7335983792666142
Chryseobacterium suipulveris	0	0	0	0.5556897902827946	0.7335983792666142
Citrobacter sp CFNIH10	0	0	0	0.5556897902827946	0.7335983792666142
Citrobacter sp MGH 55	0	0	0	0.5556897902827946	0.7335983792666142
Corynebacterium camporealensis	0	0	0	0.5556897902827946	0.7335983792666142

Species	Median g1 (resp.)	Median g2 (non-resp.)	log2FC	p	padj
Cryobacterium arcticum	0	0	0	0.5556897902827946	0.7335983792666142
Dermabacter vaginalis	0	0	0	0.5556897902827946	0.7335983792666142
Desulfosarcina widdelii	0	0	0	0.5556897902827946	0.7335983792666142
Fluviicola taffensis	0	0	0	0.5556897902827946	0.7335983792666142
Furfurilactobacillus rossiae	0	0	0	0.5556897902827946	0.7335983792666142
Herbinix luporum	0	0	0	0.5556897902827946	0.7335983792666142
Hugenholtzia roseola	0	0	0	0.5556897902827946	0.7335983792666142
Klebsiella sp PL-2018	0	0	0	0.5556897902827946	0.7335983792666142
Lacticaseibacillus zeae	0	0	0	0.5556897902827946	0.7335983792666142
Mycobacterium sp MS1601	0	0	0	0.5556897902827946	0.7335983792666142
Mycoplasma sp NEAQ87857	0	0	0	0.5556897902827946	0.7335983792666142
Neisseria lisongii	0	0	0	0.5556897902827946	0.7335983792666142
Oceanirhabdus sp W0125-5	0	0	0	0.5556897902827946	0.7335983792666142
Paenibacillus agaridevorans	0	0	0	0.5556897902827946	0.7335983792666142
Paenibacillus bovis	0	0	0	0.5556897902827946	0.7335983792666142
Paenibacillus dendritiformis	0	0	0	0.5556897902827946	0.7335983792666142
Paenibacillus harenae	0	0	0	0.5556897902827946	0.7335983792666142
Pararhizobium qamdonense	0	0	0	0.5556897902827946	0.7335983792666142
Pseudomonas parafulva	0	0	0	0.5556897902827946	0.7335983792666142
Rathayibacter sp VKM Ac-2760	0	0	0	0.5556897902827946	0.7335983792666142
Rubrobacter tropicus	0	0	0	0.5556897902827946	0.7335983792666142
Saccharomonospora xinjiangensis	0	0	0	0.5556897902827946	0.7335983792666142
Salipaludibacillus sp LMS25	0	0	0	0.5556897902827946	0.7335983792666142
Solidesulfovibrio magneticus	0	0	0	0.5556897902827946	0.7335983792666142
Sporocytophaga myxococcoides	0	0	0	0.5556897902827946	0.7335983792666142
Streptacidiphilus albus	0	0	0	0.5556897902827946	0.7335983792666142
Streptomyces decoyicus	0	0	0	0.5556897902827946	0.7335983792666142
Streptomyces noursei	0	0	0	0.5556897902827946	0.7335983792666142
Stutzerimonas stutzeri	0	0	0	0.5556897902827946	0.7335983792666142
Thermanaerosceptrum fracticalcis	0	0	0	0.5556897902827946	0.7335983792666142
Thiolapillus brandeum	0	0	0	0.5556897902827946	0.7335983792666142
Vectrevirus cee	0	0	0	0.5556897902827946	0.7335983792666142
Weissella halotolerans	0	0	0	0.5556897902827946	0.7335983792666142
Corynebacterium flavescens	0	0	0	0.5556897902827946	0.7335983792666142

Species	Median g1 (resp.)	Median g2 (non-resp.)	log2FC	p	padj
Enterococcus gallinarum	0	0	0	0.5556897902827946	0.7335983792666142
Gordonia paraffinivorans	0	0	0	0.5556897902827946	0.7335983792666142
Lactococcus paracarnosus	0	0	0	0.5556897902827946	0.7335983792666142
Lawsonia intracellularis	0	0	0	0.5556897902827946	0.7335983792666142
Streptococcus urinalis	0	0	0	0.5556897902827946	0.7335983792666142
Treponema maltophilum	0	0	0	0.5556897902827946	0.7335983792666142
Yersinia ruckeri	0	0	0	0.5556897902827946	0.7335983792666142
Acetivibrio cellulolyticus	0	0	0	0.5556897902827946	0.7335983792666142
Allofustis seminis	0	0	0	0.5556897902827946	0.7335983792666142
Aminipila butyrica	0	0	0	0.5556897902827946	0.7335983792666142
Arthrobacter sp zg-Y1171	0	0	0	0.5556897902827946	0.7335983792666142
Bacteroides propionicifaciens	0	0	0	0.5556897902827946	0.7335983792666142
Brussowvirus CHPC1230	0	0	0	0.5556897902827946	0.7335983792666142
Catenuloplanes japonicus	0	0	0	0.5556897902827946	0.7335983792666142
Cellulosilyticum lentocellum	0	0	0	0.5556897902827946	0.7335983792666142
Chlorobaculum parvum	0	0	0	0.5556897902827946	0.7335983792666142
Citrobacter arsenatis	0	0	0	0.5556897902827946	0.7335983792666142
Citrobacter freundii complex sp CFNIH9	0	0	0	0.5556897902827946	0.7335983792666142
Clostridium kluyveri	0	0	0	0.5556897902827946	0.7335983792666142
Cronobacter sakazakii	0	0	0	0.5556897902827946	0.7335983792666142
Dysgonomonas capnocytophagoides	0	0	0	0.5556897902827946	0.7335983792666142
Dysgonomonas gadei	0	0	0	0.5556897902827946	0.7335983792666142
Dysgonomonas mossii	0	0	0	0.5556897902827946	0.7335983792666142
Dysgonomonas sp HDW5A	0	0	0	0.5556897902827946	0.7335983792666142
Dysgonomonas sp HDW5B	0	0	0	0.5556897902827946	0.7335983792666142
Enterococcus sp FDAARGOS 553	0	0	0	0.5556897902827946	0.7335983792666142
Enterococcus sp FZMF	0	0	0	0.5556897902827946	0.7335983792666142
Epilithonimonas vandammei	0	0	0	0.5556897902827946	0.7335983792666142
Erysipelothrix larvae	0	0	0	0.5556897902827946	0.7335983792666142
Erysipelothrix tonsillarum	0	0	0	0.5556897902827946	0.7335983792666142
Fructobacillus fructosus	0	0	0	0.5556897902827946	0.7335983792666142
Gelria sp Kuro-4	0	0	0	0.5556897902827946	0.7335983792666142
Geoalkalibacter subterraneus	0	0	0	0.5556897902827946	0.7335983792666142
Jeotgalibaca ciconiae	0	0	0	0.5556897902827946	0.7335983792666142

Species	Median g1 (resp.)	Median g2 (non-resp.)	log2FC	p	padj
Kosakonia cowanii	0	0	0	0.5556897902827946	0.7335983792666142
Labrys sp KNU-23	0	0	0	0.5556897902827946	0.7335983792666142
Lacticigenium naphthae	0	0	0	0.5556897902827946	0.7335983792666142
Levilactobacillus zymae	0	0	0	0.5556897902827946	0.7335983792666142
Marinobacterium rhizophilum	0	0	0	0.5556897902827946	0.7335983792666142
Methanocella arvoryzae	0	0	0	0.5556897902827946	0.7335983792666142
Metschnikowia hawaiiensis	0	0	0	0.5556897902827946	0.7335983792666142
Microbacterium aurum	0	0	0	0.5556897902827946	0.7335983792666142
Moellerella wisconsensis	0	0	0	0.5556897902827946	0.7335983792666142
Pantoea alhagi	0	0	0	0.5556897902827946	0.7335983792666142
Pectobacterium odoriferum	0	0	0	0.5556897902827946	0.7335983792666142
Proteus vulgaris	0	0	0	0.5556897902827946	0.7335983792666142
Providencia alcalifaciens	0	0	0	0.5556897902827946	0.7335983792666142
Pseudoduganella violaceinigra	0	0	0	0.5556897902827946	0.7335983792666142
Raoultella sp HC6	0	0	0	0.5556897902827946	0.7335983792666142
Ruminiclostridium cellulolyticum	0	0	0	0.5556897902827946	0.7335983792666142
Ruminiclostridium josui	0	0	0	0.5556897902827946	0.7335983792666142
Sediminispirochaeta smaragdinae	0	0	0	0.5556897902827946	0.7335983792666142
Selenomonas ruminantium	0	0	0	0.5556897902827946	0.7335983792666142
Sphingobacterium multivorum	0	0	0	0.5556897902827946	0.7335983792666142
Streptococcus halotolerans	0	0	0	0.5556897902827946	0.7335983792666142
Streptomyces chromofuscus	0	0	0	0.5556897902827946	0.7335983792666142
Streptomyces sp SH5	0	0	0	0.5556897902827946	0.7335983792666142
Streptomyces sp W1SF4	0	0	0	0.5556897902827946	0.7335983792666142
Talaromyces funiculosus	0	0	0	0.5556897902827946	0.7335983792666142
Tellurirhabdus rosea	0	0	0	0.5556897902827946	0.7335983792666142
Yersinia enterocolitica	0	0	0	0.5556897902827946	0.7335983792666142
uncultured Trichococcus sp	0	0	0	0.5556897902827946	0.7335983792666142
Bradyrhizobium daqingense	0	0	0	0.5556897902827946	0.7335983792666142
Staphylococcus saprophyticus	0	0	0	0.5556897902827946	0.7335983792666142
Megamonas hypermegale	0	0	0	0.5556897902827946	0.7335983792666142
Salinimonas sediminis	0	0	0	0.5556897902827946	0.7335983792666142
Kingella denitrificans	0	0	0	0.5556897902827946	0.7335983792666142
Paenibacillus antarcticus	0	0	0	0.5556897902827946	0.7335983792666142

Species	Median g1 (resp.)	Median g2 (non-resp.)	log2FC	p	padj
Petroclostridium sp X23	0	0	0	0.5556897902827946	0.7335983792666142
Lactobacillus phage phiAQ113	0	0	0	0.5556897902827946	0.7335983792666142
Priestia filamentosa	0	0	0	0.5556897902827946	0.7335983792666142
Cokeromyces recurvatus	0	0	0	0.5556897902827946	0.7335983792666142
Duffyella gerundensis	0	0	0	0.5556897902827946	0.7335983792666142
Clostridium sp BJN0001	0	0	0	0.5556897902827946	0.7335983792666142
Veillonella magna	0	0	0	0.5556897902827946	0.7335983792666142
Weissella viridescens	0	0	0	0.5556897902827946	0.7335983792666142
Limosilactobacillus coleohominis	0	0	0	0.5556897902827946	0.7335983792666142
Mycolicibacterium aurum	0	0	0	0.5556897902827946	0.7335983792666142
Pichia kudriavzevii	0	0	0	0.5556897902827946	0.7335983792666142
Clostridium estertheticum	0	0	0	0.5556897902827946	0.7335983792666142
Bacillus thuringiensis	0	0	0	0.5556897902827946	0.7335983792666142
Caldinitratiruptor microaerophilus	0	0	0	0.5556897902827946	0.7335983792666142
Cohnella laeviribosi	0	0	0	0.5556897902827946	0.7335983792666142
Rothia phage Spartoi	0	0	0	0.5556897902827946	0.7335983792666142
Aureobasidium pullulans	0	0	0	0.5556897902827946	0.7335983792666142
Gemella sp zg-570	0	0	0	0.5556897902827946	0.7335983792666142
Schaalia suimastitidis	0	0	0	0.5556897902827946	0.7335983792666142
Arthrinium phaeospermum	0	0	0	0.5556897902827946	0.7335983792666142
Keratinibaculum paraultunense	0	0	0	0.5556897902827946	0.7335983792666142
Pseudoduganella lutea	0	0	0	0.5556897902827946	0.7335983792666142
Actinomyces respiraculi	0	0	0	0.5556897902827946	0.7335983792666142
Actinoplanes ianthinogenes	0	0	0	0.5556897902827946	0.7335983792666142
Alicyclobacillus sp SO9	0	0	0	0.5556897902827946	0.7335983792666142
Amycolatopsis vancoresmycina	0	0	0	0.5556897902827946	0.7335983792666142
Arachidicoccus soli	0	0	0	0.5556897902827946	0.7335983792666142
Arthrobacter polaris	0	0	0	0.5556897902827946	0.7335983792666142
Bacilladnaviridae sp	0	0	0	0.5556897902827946	0.7335983792666142
Caballeronia insecticola	0	0	0	0.5556897902827946	0.7335983792666142
Candidatus Puniceispirillum marinum	0	0	0	0.5556897902827946	0.7335983792666142
Desulfitobacterium metallireducens	0	0	0	0.5556897902827946	0.7335983792666142
Fictibacillus sp KU28468	0	0	0	0.5556897902827946	0.7335983792666142
Halomonas sp HG01	0	0	0	0.5556897902827946	0.7335983792666142

Species	Median g1 (resp.)	Median g2 (non-resp.)	log2FC	p	padj
Halotalea alkalilenta	0	0	0	0.5556897902827946	0.7335983792666142
Heliorestis convoluta	0	0	0	0.5556897902827946	0.7335983792666142
Kribbella qitaiheensis	0	0	0	0.5556897902827946	0.7335983792666142
Mixta calida	0	0	0	0.5556897902827946	0.7335983792666142
Mycoplasma yeatsii	0	0	0	0.5556897902827946	0.7335983792666142
Neolewinella persica	0	0	0	0.5556897902827946	0.7335983792666142
Paenibacillus donghaensis	0	0	0	0.5556897902827946	0.7335983792666142
Paenibacillus rhizovicinus	0	0	0	0.5556897902827946	0.7335983792666142
Paracoccus sp AK26	0	0	0	0.5556897902827946	0.7335983792666142
Penicillium capsulatum	0	0	0	0.5556897902827946	0.7335983792666142
Rhizobium sp CSC1952	0	0	0	0.5556897902827946	0.7335983792666142
Salinicoccus carnicancri	0	0	0	0.5556897902827946	0.7335983792666142
Sphingobium chlorophenolicum	0	0	0	0.5556897902827946	0.7335983792666142
Sphingobium sp V4	0	0	0	0.5556897902827946	0.7335983792666142
Streptomyces tanashiensis	0	0	0	0.5556897902827946	0.7335983792666142
Synechococcus sp RS9909	0	0	0	0.5556897902827946	0.7335983792666142
Vibrio nigripulchritudo	0	0	0	0.5556897902827946	0.7335983792666142
[Pseudomonas] carboxydohydrogena	0	0	0	0.5556897902827946	0.7335983792666142
Burzaovirus coli	0	0	0	0.5556897902827946	0.7335983792666142
Burzaovirus faecalis	0	0	0	0.5556897902827946	0.7335983792666142
Burzaovirus intestinihominis	0	0	0	0.5556897902827946	0.7335983792666142
Endlipuvirus intestinihominis	0	0	0	0.5556897902827946	0.7335983792666142
Kutzneria chonburiensis	0	0	0	0.5556897902827946	0.7335983792666142
Selenomonas infelix	0	0	0	0.5556897902827946	0.7335983792666142
Actinomyces slackii	0	0	0	0.5556897902827946	0.7335983792666142
Arcanobacterium haemolyticum	0	0	0	0.5556897902827946	0.7335983792666142
Bacillus atrophaeus	0	0	0	0.5556897902827946	0.7335983792666142
Bifidobacterium imperatoris	0	0	0	0.5556897902827946	0.7335983792666142
Brevibacillus humidisoli	0	0	0	0.5556897902827946	0.7335983792666142
Brevibacterium ravenspurgense	0	0	0	0.5556897902827946	0.7335983792666142
Caldicellulosiruptor morganii	0	0	0	0.5556897902827946	0.7335983792666142
Citrifermentans bremense	0	0	0	0.5556897902827946	0.7335983792666142
Corynebacterium atypicum	0	0	0	0.5556897902827946	0.7335983792666142
Corynebacterium endometrii	0	0	0	0.5556897902827946	0.7335983792666142

Species	Median g1 (resp.)	Median g2 (non-resp.)	log2FC	p	padj
Corynebacterium uberis	0	0	0	0.5556897902827946	0.7335983792666142
Enterobacter cloacae complex sp ECNIH7	0	0	0	0.5556897902827946	0.7335983792666142
Gemella sp oral taxon 928	0	0	0	0.5556897902827946	0.7335983792666142
Gordonia iterans	0	0	0	0.5556897902827946	0.7335983792666142
Halalkalibacterium halodurans	0	0	0	0.5556897902827946	0.7335983792666142
Helicobacter pylori	0	0	0	0.5556897902827946	0.7335983792666142
Kitasatospora sp MMS16-BH015	0	0	0	0.5556897902827946	0.7335983792666142
Lactobacillus amylolyticus	0	0	0	0.5556897902827946	0.7335983792666142
Liquorilactobacillus hordei	0	0	0	0.5556897902827946	0.7335983792666142
Mesoflavibacter profundi	0	0	0	0.5556897902827946	0.7335983792666142
Micropruina glycogenica	0	0	0	0.5556897902827946	0.7335983792666142
Miniimonas sp S16	0	0	0	0.5556897902827946	0.7335983792666142
Muricauda sp SCSIO 65647	0	0	0	0.5556897902827946	0.7335983792666142
Nakaseomyces glabratus	0	0	0	0.5556897902827946	0.7335983792666142
Novibacillus thermophilus	0	0	0	0.5556897902827946	0.7335983792666142
Paenibacillus sabinae	0	0	0	0.5556897902827946	0.7335983792666142
Pamirivirus faecium	0	0	0	0.5556897902827946	0.7335983792666142
Penicillium janthinellum	0	0	0	0.5556897902827946	0.7335983792666142
Peptoniphilus indolicus	0	0	0	0.5556897902827946	0.7335983792666142
Pleomorphomonas koreensis	0	0	0	0.5556897902827946	0.7335983792666142
Pseudanabaena galeata	0	0	0	0.5556897902827946	0.7335983792666142
Pseudodesulfovibrio portus	0	0	0	0.5556897902827946	0.7335983792666142
Pseudomonas putida	0	0	0	0.5556897902827946	0.7335983792666142
Pseudomonas solani	0	0	0	0.5556897902827946	0.7335983792666142
Rhizorhabdus wittichii	0	0	0	0.5556897902827946	0.7335983792666142
Rhodoferax sp AJA081-3	0	0	0	0.5556897902827946	0.7335983792666142
Rubrobacter marinus	0	0	0	0.5556897902827946	0.7335983792666142
Salinivirga cyanobacteriivorans	0	0	0	0.5556897902827946	0.7335983792666142
Scheffersomyces spartinae	0	0	0	0.5556897902827946	0.7335983792666142
Secundilactobacillus malefermentans	0	0	0	0.5556897902827946	0.7335983792666142
Skermanella stibiiresistens	0	0	0	0.5556897902827946	0.7335983792666142
Solicola gregarius	0	0	0	0.5556897902827946	0.7335983792666142
Sphingopyxis sp OPL5	0	0	0	0.5556897902827946	0.7335983792666142
Streptococcus phage phiARI0131-2	0	0	0	0.5556897902827946	0.7335983792666142

Species	Median g1 (resp.)	Median g2 (non-resp.)	log2FC	p	padj
Streptomyces changanensis	0	0	0	0.5556897902827946	0.7335983792666142
Streptomyces formicae	0	0	0	0.5556897902827946	0.7335983792666142
Streptomyces sp MMBL 11-1	0	0	0	0.5556897902827946	0.7335983792666142
Streptomyces sp WMMC500	0	0	0	0.5556897902827946	0.7335983792666142
Thermobacillus composti	0	0	0	0.5556897902827946	0.7335983792666142
Thermospira aquatica	0	0	0	0.5556897902827946	0.7335983792666142
Treponema brennaborense	0	0	0	0.5556897902827946	0.7335983792666142
Treponema sp OMZ 906	0	0	0	0.5556897902827946	0.7335983792666142
uncultured Carboxylicivirga sp	0	0	0	0.5556897902827946	0.7335983792666142
Acidihalobacter ferrooxydans	0	0	0	0.5556897902827946	0.7335983792666142
Actinospica robiniae	0	0	0	0.5556897902827946	0.7335983792666142
Alicyclobacillus acidocaldarius	0	0	0	0.5556897902827946	0.7335983792666142
Anaerophaga thermohalophila	0	0	0	0.5556897902827946	0.7335983792666142
Bifidobacterium sp ESL0728	0	0	0	0.5556897902827946	0.7335983792666142
Brachybacterium huguangmaarense	0	0	0	0.5556897902827946	0.7335983792666142
Candidatus Kuenenia stuttgartiensis	0	0	0	0.5556897902827946	0.7335983792666142
Corynebacterium nuruki	0	0	0	0.5556897902827946	0.7335983792666142
Delmidovirus copri	0	0	0	0.5556897902827946	0.7335983792666142
Desulfosporosinus youngiae	0	0	0	0.5556897902827946	0.7335983792666142
Geomonas sp RF6	0	0	0	0.5556897902827946	0.7335983792666142
Halopseudomonas sabulinigri	0	0	0	0.5556897902827946	0.7335983792666142
Lactococcus formosensis	0	0	0	0.5556897902827946	0.7335983792666142
Lysinibacillus sp CD3-6	0	0	0	0.5556897902827946	0.7335983792666142
Mariniblastus fucicola	0	0	0	0.5556897902827946	0.7335983792666142
Mesorhizobium sp L-8-10	0	0	0	0.5556897902827946	0.7335983792666142
Methylomagnum ishizawai	0	0	0	0.5556897902827946	0.7335983792666142
Methyloversatilis sp RAC08	0	0	0	0.5556897902827946	0.7335983792666142
Micromonospora sp NBC 01813	0	0	0	0.5556897902827946	0.7335983792666142
Mycobacterium gallinarum	0	0	0	0.5556897902827946	0.7335983792666142
Nevskia soli	0	0	0	0.5556897902827946	0.7335983792666142
Paenibacillus sp FSL R7-0331	0	0	0	0.5556897902827946	0.7335983792666142
Paraglaciecola polaris	0	0	0	0.5556897902827946	0.7335983792666142
Pontibacillus litoralis	0	0	0	0.5556897902827946	0.7335983792666142
Pseudarthrobacter sp NS4	0	0	0	0.5556897902827946	0.7335983792666142

Species	Median g1 (resp.)	Median g2 (non-resp.)	log2FC	p	padj
Pseudomonas sp ADAK13	0	0	0	0.5556897902827946	0.7335983792666142
Rahnella inusitata	0	0	0	0.5556897902827946	0.7335983792666142
Roseovarius indicus	0	0	0	0.5556897902827946	0.7335983792666142
Sanguibacter keddieii	0	0	0	0.5556897902827946	0.7335983792666142
Schnuerera ultunensis	0	0	0	0.5556897902827946	0.7335983792666142
Shewanella pealeana	0	0	0	0.5556897902827946	0.7335983792666142
Shinella zoogloeoides	0	0	0	0.5556897902827946	0.7335983792666142
Sphingomonas astaxanthinifaciens	0	0	0	0.5556897902827946	0.7335983792666142
Sphingopyxis terrae	0	0	0	0.5556897902827946	0.7335983792666142
Streptomyces sp NBC 01451	0	0	0	0.5556897902827946	0.7335983792666142
Succinatimonas hippei	0	0	0	0.5556897902827946	0.7335983792666142
Thalassolituus sp C2-1	0	0	0	0.5556897902827946	0.7335983792666142
Tissierella sp Yu-01	0	0	0	0.5556897902827946	0.7335983792666142
Vibrio artabrorum	0	0	0	0.5556897902827946	0.7335983792666142
Altererythrobacter sp BO-6	0	0	0	0.5556897902827946	0.7335983792666142
Archangium violaceum	0	0	0	0.5556897902827946	0.7335983792666142
Arthrobacter sp YA7-1	0	0	0	0.5556897902827946	0.7335983792666142
Aspergillus westerdijkiae	0	0	0	0.5556897902827946	0.7335983792666142
Bradyrhizobium roseus	0	0	0	0.5556897902827946	0.7335983792666142
Burkholderia sp MSMB0856	0	0	0	0.5556897902827946	0.7335983792666142
Candidatus Rhabdochlamydia porcellionis	0	0	0	0.5556897902827946	0.7335983792666142
Cohnella candidum	0	0	0	0.5556897902827946	0.7335983792666142
Corynebacterium capitovis	0	0	0	0.5556897902827946	0.7335983792666142
Cupriavidus basilensis	0	0	0	0.5556897902827946	0.7335983792666142
Desulfitibacter alkalitolerans	0	0	0	0.5556897902827946	0.7335983792666142
Dietzia lutea	0	0	0	0.5556897902827946	0.7335983792666142
Diorhovirus copri	0	0	0	0.5556897902827946	0.7335983792666142
Diorhovirus intestinalis	0	0	0	0.5556897902827946	0.7335983792666142
Fohxhuevirus gastrointestinalis	0	0	0	0.5556897902827946	0.7335983792666142
Frondihabitans sucicola	0	0	0	0.5556897902827946	0.7335983792666142
Granulicella tundricola	0	0	0	0.5556897902827946	0.7335983792666142
Inhella inkyongensis	0	0	0	0.5556897902827946	0.7335983792666142
Lahndsivirus rarus	0	0	0	0.5556897902827946	0.7335983792666142
Maribacter sp HTCC2170	0	0	0	0.5556897902827946	0.7335983792666142

Species	Median g1 (resp.)	Median g2 (non-resp.)	log2FC	p	padj
Methanobrevibacter millerae	0	0	0	0.5556897902827946	0.7335983792666142
Methylomonas montana	0	0	0	0.5556897902827946	0.7335983792666142
Micavibrio aeruginosavorus	0	0	0	0.5556897902827946	0.7335983792666142
Microbacterium sp 4R-513	0	0	0	0.5556897902827946	0.7335983792666142
Mycolicibacterium rufum	0	0	0	0.5556897902827946	0.7335983792666142
Mycoplasmopsis arginini	0	0	0	0.5556897902827946	0.7335983792666142
Nodosilinea sp E11	0	0	0	0.5556897902827946	0.7335983792666142
Nonomuraea phyllanthi	0	0	0	0.5556897902827946	0.7335983792666142
Paenibacillus senegalensis	0	0	0	0.5556897902827946	0.7335983792666142
Pedobacter endophyticus	0	0	0	0.5556897902827946	0.7335983792666142
Plantibacter sp PA-3-X8	0	0	0	0.5556897902827946	0.7335983792666142
Pseudobacteroides cellulosolvens	0	0	0	0.5556897902827946	0.7335983792666142
Pseudodesulfovibrio tunisiensis	0	0	0	0.5556897902827946	0.7335983792666142
Pseudomonas sp WJP1	0	0	0	0.5556897902827946	0.7335983792666142
Rhodanobacter denitrificans	0	0	0	0.5556897902827946	0.7335983792666142
Roseomonas mucosa	0	0	0	0.5556897902827946	0.7335983792666142
Saccharomonospora iraqiensis	0	0	0	0.5556897902827946	0.7335983792666142
Sciscionella marina	0	0	0	0.5556897902827946	0.7335983792666142
Sebaldella termitidis	0	0	0	0.5556897902827946	0.7335983792666142
Shewanella cyperi	0	0	0	0.5556897902827946	0.7335983792666142
Spiribacter halobius	0	0	0	0.5556897902827946	0.7335983792666142
Thermococcus nautili	0	0	0	0.5556897902827946	0.7335983792666142
Tichowtungia aerotolerans	0	0	0	0.5556897902827946	0.7335983792666142
Trichococcus shcherbakoviae	0	0	0	0.5556897902827946	0.7335983792666142
Odoribacter laneus	2.831197760862315e-7	2.472518946466292e-6	-1.4363267169799536	0.5565216129897126	0.7337298108101211
Segatella hominis	3.8110961589105143e-6	3.706350854694296e-6	0.031756758380149984	0.5565216129897126	0.7337298108101211
Lactococcus raffinolactis	0	0	0	0.55793569486749806	0.734627550929347
Lactobacillus johnsonii	0	0	0	0.55793569486749806	0.734627550929347
Corynebacterium jeikeium	0	0	0	0.5628033649843449	0.7395789792974604
Leuconostoc citreum	0	0	0	0.5628033649843449	0.7395789792974604
Prevotella dentasini	0	0	0	0.5628033649843449	0.7395789792974604
Peptoniphilus sp SAHP1	1.3284587839545658e-6	0	1.219375345241505	0.5717235253484472	0.7484385694849893
Muribaculum gordoncarteri	0	2.5275681862069594e-7	-0.3251063902228827	0.5717235253484472	0.7484385694849893
Citrobacter freundii	0	2.4808318526901893e-7	-0.3197140936357452	0.5717235253484472	0.7484385694849893

Species	Median g1 (resp.)	Median g2 (non-resp.)	log2FC	p	padj
Bacteroides sp MSB163	1.0247492361299247e-6	8.94958510099537e-6	-2.296893124115451	0.5721596249755507	0.7484385694849893
Phascolarctobacterium sp Marseille-Q4147	4.73352101591784e-6	7.557672952825398e-6	-0.5777971593513402	0.5721596249755507	0.7484385694849893
Prevotella melaninogenica	2.3906631362259168e-6	3.847946119176798e-6	-0.5158062054255272	0.5721596249755507	0.7484385694849893
Lacticaseibacillus rhamnosus	1.7814048818831574e-6	1.4757869310727974e-6	0.1679266093569389	0.5721596249755507	0.7484385694849893
Bacteroides eggerthii	3.582649771866322e-4	1.2720411944862518e-4	1.4866056822931533	0.5739513698312535	0.7498034844470873
Alistipes putredinis	0.00686190182520929	0.00330971140831637	1.0516774590612172	0.5739513698312535	0.7498034844470873
Oxalobacter aliiformigenes	4.599172884748407e-7	2.2420438830765145e-6	-1.15101698313977	0.5820972925193774	0.7594550613338752
Paraclostridium sordellii	1.446452334574068e-6	2.1697348887728803e-7	1.0073934342657123	0.5820972925193774	0.7594550613338752
Streptococcus sp NSJ-72	5.080696917538052e-7	1.3051490741272467e-7	0.4157230846140719	0.5898767906195113	0.7631080836701463
Aspergillus flavus	5.308388652167336e-7	2.4808318526901893e-7	0.294608340353482	0.5898767906195113	0.7631080836701463
Haemophilus haemolyticus	0	0	0	0.5937641313338187	0.7631080836701463
Anaerococcus lactolyticus	0	0	0	0.5937641313338187	0.7631080836701463
Thermocaproicibacter melissae	0	0	0	0.5937641313338187	0.7631080836701463
Propionibacterium freudenreichii	0	0	0	0.5937641313338187	0.7631080836701463
Faecalispora jeddahensis	0	0	0	0.5937641313338187	0.7631080836701463
Prevotella multiformis	0	0	0	0.5937641313338187	0.7631080836701463
Bacteroides stercorirosoris	8.493593282586945e-7	1.7344536260508088e-6	-0.5642270295934205	0.595637123521487	0.7631080836701463
Bifidobacterium ruminantium	7.64423395432825e-5	1.3464191360575111e-5	2.4206368674982506	0.602699834770017	0.7631080836701463
Clostridium sp C1	1.0063459433732655e-6	2.2602511614717795e-6	-0.7004127268037312	0.602699834770017	0.7631080836701463
Gemella haemolysans	1.3955639661517711e-6	1.0760796887605513e-6	0.2065035151839361	0.602699834770017	0.7631080836701463
Barnesiella intestinihominis	0.0017315309087659828	0.001831400216181537	-0.08085354755693643	0.6057226196064835	0.7631080836701463
Alistipes megaguti	7.930172861778797e-6	7.571459868579557e-6	0.05914716062982623	0.6057226196064835	0.7631080836701463
Bacteroides sp D2	4.650346964445906e-6	1.1005811697552712e-5	-1.0873215803315013	0.6062817742964273	0.7631080836701463
Mogibacterium pumilum	1.172026710903617e-6	1.5945680000644312e-6	-0.25645250236534417	0.6062817742964273	0.7631080836701463
Beauveria bassiana	0	0	0	0.6069054272179508	0.7631080836701463
Clostridium beijerinckii	0	0	0	0.6069054272179508	0.7631080836701463
Streptococcus macedonicus	0	0	0	0.6069054272179508	0.7631080836701463
Anaerococcus provencensis	0	0	0	0.6069054272179508	0.7631080836701463
Enterococcus montenegrensis	0	0	0	0.6069054272179508	0.7631080836701463
Kluyvera cryocrescens	0	0	0	0.6069054272179508	0.7631080836701463
Bifidobacterium psychraerophilum	0	0	0	0.6069054272179508	0.7631080836701463
Clostridium saccharoperbutylacetonicum	0	0	0	0.6069054272179508	0.7631080836701463
Granulosicoccus antarcticus	0	0	0	0.6069054272179508	0.7631080836701463
Staphylococcus equorum	0	0	0	0.6069054272179508	0.7631080836701463

Species	Median g1 (resp.)	Median g2 (non-resp.)	log2FC	p	padj
Limosilactobacillus frumenti	0	0	0	0.6069054272179508	0.7631080836701463
Penicillium polonicum	0	0	0	0.6069054272179508	0.7631080836701463
Corynebacterium provencense	0	0	0	0.6069054272179508	0.7631080836701463
Parabacteroides phage YZ-2015b	0	0	0	0.6069054272179508	0.7631080836701463
Ruminiclostridium herbifermentans	0	0	0	0.6069054272179508	0.7631080836701463
Comamonas aquatica	0	0	0	0.6069054272179508	0.7631080836701463
Fusobacterium ulcerans	0	0	0	0.6069054272179508	0.7631080836701463
Kluyvera ascorbata	0	0	0	0.6069054272179508	0.7631080836701463
Leptotrichia trevisanii	0	0	0	0.6069054272179508	0.7631080836701463
Selenomonas timonae	0	0	0	0.6069054272179508	0.7631080836701463
Pasteurella bettyae	0	0	0	0.6069054272179508	0.7631080836701463
Klebsiella pasteurii	0	0	0	0.6069054272179508	0.7631080836701463
Klebsiella sp FDAARGOS 511	0	0	0	0.6069054272179508	0.7631080836701463
Klebsiella variicola	0	0	0	0.6069054272179508	0.7631080836701463
Microbacterium testaceum	0	0	0	0.6069054272179508	0.7631080836701463
Cladosporium sphaerospermum	0	0	0	0.6069054272179508	0.7631080836701463
Corynebacterium pseudodiphtheriticum	0	0	0	0.6069831763324417	0.7631080836701463
Klebsiella quasipneumoniae	0	0	0	0.6069831763324417	0.7631080836701463
Anaerococcus prevotii	0	0	0	0.6069831763324417	0.7631080836701463
Campylobacter ureolyticus	0	0	0	0.6069831763324417	0.7631080836701463
Porphyromonas bennonis	0	0	0	0.6069831763324417	0.7631080836701463
Enterococcus lactis	0	0	0	0.6069831763324417	0.7631080836701463
Enterococcus mundtii	0	0	0	0.6069831763324417	0.7631080836701463
Leuconostoc pseudomesenteroides	0	0	0	0.6069831763324417	0.7631080836701463
Staphylococcus warneri	0	0	0	0.6069831763324417	0.7631080836701463
Lactobacillus acidophilus	0	0	0	0.6069831763324417	0.7631080836701463
Cardiobacterium valvarum	0	0	0	0.6069831763324417	0.7631080836701463
Lacrimispora sphenoides	0	0	0	0.6069831763324417	0.7631080836701463
Prevotella histicola	0	0	0	0.6069831763324417	0.7631080836701463
Raoultella terrigena	0	0	0	0.6069831763324417	0.7631080836701463
Streptococcus acidominimus	0	0	0	0.6069831763324417	0.7631080836701463
Raoultella ornithinolytica	0	0	0	0.6069831763324417	0.7631080836701463
Adlercreutzia equolifaciens	6.55888100087841e-4	1.255002406307841e-4	2.376507501280575	0.6164619694031459	0.7673228488719903
Sellimonas intestinalis	4.608743943793362e-5	1.1086455125912295e-5	1.9619510985330304	0.6164619694031459	0.7673228488719903

Species	Median g1 (resp.)	Median g2 (non-resp.)	log2FC	p	padj
Enterocloster clostridioformis	6.3395421819862255e-05	2.377422462466614e-4	-1.8904238497970085	0.6164619694031459	0.7673228488719903
Anaerostipes hadrus	0.014178460363628987	0.004543617886448515	1.6415717259250182	0.6164619694031459	0.7673228488719903
Alistipes communis	0.0016527499152971053	8.858140596419064e-4	0.8990375344753109	0.6164619694031459	0.7673228488719903
Subdoligranulum variabile	6.30197278660166e-4	3.6017279564880733e-4	0.8054017554785586	0.6164619694031459	0.7673228488719903
Faecalibacterium sp HTF-F	2.0457124530514367e-4	1.225512303263599e-4	0.7345291016163754	0.6164619694031459	0.7673228488719903
Saccharomyces cerevisiae	1.339076249761919e-5	7.703228627555175e-6	0.7255204351793709	0.6164619694031459	0.7673228488719903
Phocaeicola coprocola	5.491458045723469e-5	8.185171740306692e-5	-0.5673070691528511	0.6164619694031459	0.7673228488719903
[Clostridium] hylemonae	3.985344219177916e-5	2.7500186151890314e-5	0.5194862983460363	0.6164619694031459	0.7673228488719903
Phocaeicola barnesiae	2.2276469385125435e-5	1.6176547655506213e-5	0.4384321485758336	0.6164619694031459	0.7673228488719903
Streptococcus sp A12	3.5546624254090945e-6	4.975093765817624e-6	-0.3916172223492426	0.6164619694031459	0.7673228488719903
Intestinimonas butyriciproducens	7.535259942234441e-4	8.771165862471249e-4	-0.21884189298758763	0.6164619694031459	0.7673228488719903
Faecalibacterium sp I4-1-79	1.3187619509362386e-4	1.1575119421707563e-4	0.18664536051330094	0.6164619694031459	0.7673228488719903
Dorea longicatena	0.006644333458338446	0.007284806159030502	-0.13274701749531828	0.6164619694031459	0.7673228488719903
Candidatus Nanosynbacter sp HMT-352	4.54037592791467e-6	4.7180850394784316e-6	-0.045548205973334414	0.6164619694031459	0.7673228488719903
Streptococcus sp oral taxon 061	3.7081473002184654e-7	6.266812779507625e-7	-0.24689800633477504	0.6241245162128684	0.7763783553634936
Streptococcus canis	5.716644238365771e-7	0	0.6522932113952349	0.6316674107896681	0.7838151648436501
Pseudobutyrivibrio ruminis	1.8540736501092327e-7	0	0.2453829262015322	0.6316674107896681	0.7838151648436501
Rhodococcus fascians	0	0	0	0.6316674107896681	0.7838151648436501
Streptococcus gwangjuense	0	0	0	0.6316674107896681	0.7838151648436501
Butyricimonas paravirosa	1.1938778853986444e-4	4.604283127390322e-5	1.3556422641138792	0.6378348845703727	0.7909784088360315
Bifidobacterium breve	1.6968338268278786e-4	3.7097194527552324e-5	2.1635659410696597	0.6394022407214592	0.7924317194841091
Streptococcus peroris	1.9055480794552572e-7	3.2209168919698035e-7	-0.15118819603160968	0.6525194161725885	0.8063543724258803
Eubacterium xylanophilum	0	0	0	0.6526480998377134	0.8063543724258803
Carjivirus hominis	0	0	0	0.6526480998377134	0.8063543724258803
Ligilactobacillus murinus	0	0	0	0.6526480998377134	0.8063543724258803
Lacticaseibacillus casei	0	0	0	0.6526480998377134	0.8063543724258803
Dialister micraerophilus	2.2995864423742035e-7	0	0.2986098076322935	0.6554308638719771	0.8082975084304259
Arabiibacter massiliensis	9.270368250546164e-8	0	0.1279022265007203	0.6554308638719771	0.8082975084304259
Weissella cibaria	9.270368250546164e-8	0	0.1279022265007203	0.6554308638719771	0.8082975084304259
Oscillibacter ruminantium	0	4.1402404825678827e-7	-0.4998066562192964	0.6592043578028524	0.8124511273289767
Streptococcus sp KS 6	1.3905552375819246e-6	6.967566245731809e-7	0.49456609604767027	0.6641455588201837	0.818037922480423
Streptococcus lactarius	2.7603322793780655e-6	2.4818145959577167e-6	0.11102076777396805	0.6677137997839668	0.8219277977684702
Anaeroglobus geminatus	6.927392958093586e-6	1.914704559735344e-6	1.4434968356641518	0.6701941278483534	0.8234635390607603
Olsenella timonensis	7.23226167287034e-7	3.673490185112549e-6	-1.4393883015225564	0.6701941278483534	0.8234635390607603

Species	Median g1 (resp.)	Median g2 (non-resp.)	log2FC	p	padj
Rikenella microfusus	5.995091557423318e-6	2.415631975371622e-6	1.0341904036288307	0.6701941278483534	0.8234635390607603
Bacteroides sp DH3716P	4.036345633433316e-6	2.1794749050164737e-5	-2.178252325919733	0.6727796304677776	0.8241957265836878
Actinomyces sp oral taxon 414	1.2485404257537078e-5	5.96924361241344e-6	0.952324779702062	0.6727796304677776	0.8241957265836878
Lancefieldella rimae	8.686089405271705e-6	4.7726075228302225e-6	0.7466911790254445	0.6727796304677776	0.8241957265836878
Bacteroides zhangwenhongii	7.024052804720451e-6	8.013391836047335e-6	-0.16773900976109438	0.6727796304677776	0.8241957265836878
Parvimonas micra	1.1722044077802701e-5	1.646050836605828e-5	-0.4567650738042577	0.6732576868350784	0.8241957265836878
Eggerthella sp YY7918	1.54913928077108e-5	1.3113327193542132e-5	0.22465510810249625	0.6732576868350784	0.8241957265836878
Turicimonas muris	0	0	0	0.6782456123960454	0.824778544257404
Limosilactobacillus mucosae	0	0	0	0.6782456123960454	0.824778544257404
Streptococcus massiliensis	0	0	0	0.6782456123960454	0.824778544257404
Citrobacter braakii	0	0	0	0.6782456123960454	0.824778544257404
Streptococcus gallolyticus	7.622192317821029e-7	0	0.8173934162731256	0.6808645675964644	0.824778544257404
Roseburia hominis	8.118766865086861e-4	0.0031513604885232524	-1.955324106774952	0.6819650937297996	0.824778544257404
Enterocloster bolteae	0.0022590179893439834	7.005403308773141e-4	1.687736305794809	0.6819650937297996	0.824778544257404
Phocaeicola coprophilus	1.4624229305255269e-5	4.4686231632016186e-5	-1.547974420813778	0.6819650937297996	0.824778544257404
Streptococcus anginosus	3.183236081380388e-5	7.470443941534811e-5	-1.2052594136133605	0.6819650937297996	0.824778544257404
Parabacteroides distasonis	0.0011867705671383287	0.0027096229514689965	-1.1903682594720202	0.6819650937297996	0.824778544257404
Bacteroides stercoris	6.486409605499457e-4	0.0014511054355150575	-1.1604317054228146	0.6819650937297996	0.824778544257404
Blautia luti	0.002738824496532597	0.0012842123135047981	1.09207677388257	0.6819650937297996	0.824778544257404
Mediterraneibacter gnavus	0.0021016292537039604	0.0010409367391171946	1.0129268016885944	0.6819650937297996	0.824778544257404
Flavonifractor plautii	7.654017766536257e-4	0.0015096314789118704	-0.9789789449344675	0.6819650937297996	0.824778544257404
Arachnia propionica	2.644236205974353e-5	1.663475130048997e-5	0.6379834347888276	0.6819650937297996	0.824778544257404
Bacteroides ovatus	2.0883749188820028e-4	3.2205448714746907e-4	-0.6225050450546763	0.6819650937297996	0.824778544257404
Lancefieldella parvula	3.987114166043949e-6	2.7768658684020348e-6	0.4010156810303985	0.6819650937297996	0.824778544257404
Faecalibacterium duncaniae	1.2522771464595434e-4	1.62020895069037e-4	-0.36902818831841017	0.6819650937297996	0.824778544257404
Holdemania massiliensis	3.1543016459477155e-6	3.83505448369757e-6	-0.21892617196489791	0.6819650937297996	0.824778544257404
Faecalibacterium sp I3-3-89	1.3083724539531355e-4	1.4107879320754505e-4	-0.10793322854474958	0.6819650937297996	0.824778544257404
Prevotella intermedia	5.66239552172463e-7	2.4567336656935067e-6	-1.1421045652870312	0.6950503761849752	0.8390816257695403
Shuttleworthia satelles	2.2995864423742035e-7	4.6066279633336255e-7	-0.24801335325579332	0.6950503761849752	0.8390816257695403
Dialister succinatiphilus	1.698718656517389e-6	1.5418053579019426e-6	0.0864210242864577	0.6950503761849752	0.8390816257695403
Denitrobacterium detoxificans	0	0	0	0.6962026725069004	0.8390816257695403
Lachnospira multipara	0	0	0	0.6996533915473562	0.8390816257695403
Streptococcus orisratti	0	0	0	0.6996533915473562	0.8390816257695403
Campylobacter hominis	0	0	0	0.6996533915473562	0.8390816257695403

Species	Median g1 (resp.)	Median g2 (non-resp.)	log2FC	p	padj
Butyrivibrio hungatei	0	0	0	0.6996533915473562	0.8390816257695403
Listeria monocytogenes	0	0	0	0.6996533915473562	0.8390816257695403
Streptococcus phage PH10	0	0	0	0.6996533915473562	0.8390816257695403
Bacteroides sp ZJ-18	0	0	0	0.6996533915473562	0.8390816257695403
Mycobacterium tuberculosis	0	0	0	0.6996533915473562	0.8390816257695403
Fusobacterium hwasookii	0	0	0	0.6996533915473562	0.8390816257695403
Hoylesella enoeca	0	0	0	0.6996533915473562	0.8390816257695403
Bacteroides luhongzhouii	8.493593282586945e-7	2.312868207746512e-6	-0.8410552455214582	0.702766134376241	0.8423106060346813
Acidaminococcus intestini	7.514262355671808e-5	4.737579117442952e-6	3.7301902324086265	0.7064838843760959	0.8447456469508927
Butyricimonas faecihominis	6.286374053696984e-5	3.741781142545576e-5	0.7332217205635861	0.7064838843760959	0.8447456469508927
Streptococcus pneumoniae	1.4760566719824513e-6	2.139419578245978e-6	-0.34245352028467	0.7064838843760959	0.8447456469508927
Selenomonas sputigena	8.865652512917488e-7	1.3683442806176397e-6	-0.32811681737569753	0.7064838843760959	0.8447456469508927
Peptoniphilus grossensis	9.270368250546164e-8	0	0.1279022265007203	0.7092585209309872	0.8464939839928651
Candidatus Nanosynbacter lyticus	0	1.3051490741272467e-7	-0.1769800161572092	0.7189419735997014	0.8464939839928651
Toutatisvirus toutatis	2.41075389095678e-7	4.357268097477316e-7	-0.21019050450023544	0.7261840560286119	0.8464939839928651
Streptococcus sp oral taxon 431	7.465310244963753e-7	5.223566013599032e-7	0.19818593064185083	0.7315914776242494	0.8464939839928651
Bosea sp REN20	0	0	0	0.7316005889599014	0.8464939839928651
Escherichia marmotae	0	0	0	0.7316005889599014	0.8464939839928651
Lactococcus carnosus	0	0	0	0.7316005889599014	0.8464939839928651
Desulfovibrio sp G11	0	0	0	0.7316005889599014	0.8464939839928651
Fastidiosipila sanguinis	0	0	0	0.7316005889599014	0.8464939839928651
Streptobacillus moniliformis	0	0	0	0.7316005889599014	0.8464939839928651
Berryella wangjianweii	0	0	0	0.7316005889599014	0.8464939839928651
Megamonas funiformis	0	0	0	0.7316005889599014	0.8464939839928651
Megamonas rupellensis	0	0	0	0.7316005889599014	0.8464939839928651
Carnobacterium divergens	0	0	0	0.7316005889599014	0.8464939839928651
Clostridium cellulovorans	0	0	0	0.7316005889599014	0.8464939839928651
Lactobacillus sp JM1	0	0	0	0.7316005889599014	0.8464939839928651
Leuconostoc garlicum	0	0	0	0.7316005889599014	0.8464939839928651
Streptococcus troglodytae	0	0	0	0.7316005889599014	0.8464939839928651
Buchnera aphidicola	0	0	0	0.7316005889599014	0.8464939839928651
Clostridium tetani	0	0	0	0.7316005889599014	0.8464939839928651
Corynebacterium testudinoris	0	0	0	0.7316005889599014	0.8464939839928651
Enterobacter mori	0	0	0	0.7316005889599014	0.8464939839928651

Species	Median g1 (resp.)	Median g2 (non-resp.)	log2FC	p	padj
Franconibacter pulveris	0	0	0	0.7316005889599014	0.8464939839928651
Heyndrickxia coagulans	0	0	0	0.7316005889599014	0.8464939839928651
Tumebacillus avium	0	0	0	0.7316005889599014	0.8464939839928651
uncultured Pseudodesulfovibrio sp	0	0	0	0.7316005889599014	0.8464939839928651
Carnobacterium viridans	0	0	0	0.7316005889599014	0.8464939839928651
Coriobacterium glomerans	0	0	0	0.7316005889599014	0.8464939839928651
Dehalobacter restrictus	0	0	0	0.7316005889599014	0.8464939839928651
Oxalobacter formigenes	0	0	0	0.7316005889599014	0.8464939839928651
Porphyromonas cangingivalis	0	0	0	0.7316005889599014	0.8464939839928651
Porphyromonas canoris	0	0	0	0.7316005889599014	0.8464939839928651
uncultured Caproiciproducens sp	0	0	0	0.7316005889599014	0.8464939839928651
uncultured Sporomusa sp	0	0	0	0.7316005889599014	0.8464939839928651
Fusobacterium mortiferum	0	0	0	0.7316005889599014	0.8464939839928651
Fusobacterium varium	0	0	0	0.7316005889599014	0.8464939839928651
Lactiplantibacillus pentosus	0	0	0	0.7316005889599014	0.8464939839928651
Mycolicibacterium neoaurum	0	0	0	0.7316005889599014	0.8464939839928651
Salicibibacter cibarius	0	0	0	0.7316005889599014	0.8464939839928651
Bordetella hinzii	0	0	0	0.7316005889599014	0.8464939839928651
Heliomicrobium modesticaldum	0	0	0	0.7316005889599014	0.8464939839928651
Klebsiella aerogenes	0	0	0	0.7316005889599014	0.8464939839928651
Staphylococcus simiae	0	0	0	0.7316005889599014	0.8464939839928651
Chakrabartyella piscis	0	0	0	0.7316005889599014	0.8464939839928651
Lactobacillus kitasatonis	0	0	0	0.7316005889599014	0.8464939839928651
Mameliella alba	0	0	0	0.7316005889599014	0.8464939839928651
Paracoccus versutus	0	0	0	0.7316005889599014	0.8464939839928651
Treponema sp OMZ 788	0	0	0	0.7316005889599014	0.8464939839928651
Gallibacterium anatis	0	0	0	0.7316005889599014	0.8464939839928651
Aerococcus loyolae	0	0	0	0.7316005889599014	0.8464939839928651
Enterococcus innesii	0	0	0	0.7316005889599014	0.8464939839928651
Facklamia languida	0	0	0	0.7316005889599014	0.8464939839928651
Klebsiella sp BDA134-6	0	0	0	0.7316005889599014	0.8464939839928651
Streptococcus porcinus	0	0	0	0.7316005889599014	0.8464939839928651
uncultured Bacteroides sp	0	0	0	0.7316005889599014	0.8464939839928651
Terrisporobacter petrolearius	0	0	0	0.7316005889599014	0.8464939839928651

Species	Median g1 (resp.)	Median g2 (non-resp.)	log2FC	p	padj
Butyricimonas virosa	2.4229105922335246e-4	1.666496622653311e-5	3.7837204071444694	0.7404622722295098	0.8531999406299478
Gordonibacter urolithinfaciens	9.617694977003364e-5	3.0052737686701752e-5	1.6458936836208953	0.7404622722295098	0.8531999406299478
Clostridium celatum	3.565610255285018e-6	9.213255926667251e-7	1.24870568626494	0.7404622722295098	0.8531999406299478
Akkermansia muciniphila	3.4449700189675165e-6	1.0150654980745658e-5	-1.3268828620953368	0.7417704634866991	0.8531999406299478
Collinsella stercoris	3.919299063695933e-6	6.431126173183905e-6	-0.5951281001710216	0.7417704634866991	0.8531999406299478
Bacteroides humanifaecis	6.278013776333144e-6	7.25343509177954e-6	-0.18144991412152486	0.7417704634866991	0.8531999406299478
Dialister pneumosintes	5.070206918081505e-6	1.474040199416104e-6	1.294876753043451	0.74254595147204006	0.8531999406299478
Paraprevotella clara	1.590588407048345e-5	6.610411666972441e-6	1.151479061920499	0.74254595147204006	0.8531999406299478
Oribacterium asaccharolyticum	1.0413032291872776e-6	1.166539979753864e-6	-0.0859023531915129	0.74254595147204006	0.8531999406299478
Agathobaculum sp NTUH-O15-33	4.808027464896089e-6	5.0216265530873515e-6	-0.05210496279218031	0.74254595147204006	0.8531999406299478
Ruminococcus champanellensis	3.420696509640909e-5	4.982470889995966e-5	-0.5296691773560753	0.7429310860275742	0.8531999406299478
Olsenella sp oral taxon 807	2.139672206562954e-5	1.575173529347922e-5	0.4189770476259467	0.7429310860275742	0.8531999406299478
Prevotella disiens	4.483060107825628e-6	3.760858305217531e-6	0.20375959780598427	0.7429310860275742	0.8531999406299478
Bacteroides fragilis	0.0051954712825942745	4.6042946137573676e-4	3.4933502534366183	0.7502693385046326	0.8567178087540078
Hungatella hathewayi	7.990938521611622e-4	2.195208279830332e-4	1.8592543167360824	0.7502693385046326	0.8567178087540078
Lachnospira eligens	1.0760500426368826e-4	3.477417023894717e-4	-1.6830683106351998	0.7502693385046326	0.8567178087540078
Phocaeicola vulgatus	0.004460397792007402	0.010684284033169799	-1.2600575637099232	0.7502693385046326	0.8567178087540078
Faecalitalea cylindroides	5.503089055750351e-5	3.0485634285906022e-5	0.8315286919173035	0.7502693385046326	0.8567178087540078
Pusillibacter faecalis	1.8867312696304418e-4	3.145455675440649e-4	-0.7343330669874341	0.7502693385046326	0.8567178087540078
Thomasclavelia ramosa	7.645344631177897e-5	1.1184413625567486e-4	-0.5429301147453447	0.7502693385046326	0.8567178087540078
Bacteroides gallinarum	6.5833597218051195e-6	9.449380841689528e-6	-0.46250839580152814	0.7502693385046326	0.8567178087540078
Roseburia inulinivorans	0.0022894240918028903	0.003147453427921564	-0.45902857658977136	0.7502693385046326	0.8567178087540078
Schaalia sp HMT-172	2.0778093617962996e-4	2.3648713879912824e-4	-0.18585940068469017	0.7502693385046326	0.8567178087540078
Streptococcus sp Marseille-Q6470	4.635184125273082e-7	0	0.5494408964092794	0.757730258176996	0.8647445543204443
Ezakiella coagulans	0	0	0	0.7669800766183361	0.8743049337560556
Bifidobacterium choerinum	0	0	0	0.7669800766183361	0.8743049337560556
Streptococcus sinensis	2.527278177003487e-7	3.780193334757782e-7	-0.13752313714703396	0.7687518242045348	0.8753287816510726
Ligilactobacillus salivarius	7.23226167287034e-7	8.76970899266354e-7	-0.12329422009528511	0.7687518242045348	0.8753287816510726
Bacillus paralicheniformis	5.5226085857505555e-6	2.9010810691173428e-6	0.7415750770487682	0.7776085351078856	0.8844083452645873
Bacteroides timonensis	7.885754114869289e-7	1.4614575568794363e-6	-0.460701915484235	0.7776085351078856	0.8844083452645873
Caproicibacterium argilliputei	6.4310941169530175e-6	7.346171491407682e-6	-0.16753992284363148	0.7786177188876373	0.8849879467961245
Clostridium bornimense	0	0	0	0.7803261985971816	0.8849879467961245
Enorma timonensis	0	0	0	0.7803261985971816	0.8849879467961245
Morganella morganii	0	0	0	0.7803261985971816	0.8849879467961245

Species	Median g1 (resp.)	Median g2 (non-resp.)	log2FC	p	padj
Prevotella dentalis	0	0	0	0.7803261985971816	0.8849879467961245
Sodaliphilus pleomorphus	0	0	0	0.7899905138162662	0.8949355509823614
Limosilactobacillus reuteri	0	0	0	0.7899905138162662	0.8949355509823614
Aggregatibacter aphrophilus	0	0	0	0.7970303567388294	0.8988457146339978
Serratia marcescens	0	0	0	0.7970303567388294	0.8988457146339978
Bacteroides coprosuis	0	0	0	0.7970303567388294	0.8988457146339978
Prevotella fusca	0	0	0	0.7970303567388294	0.8988457146339978
Petrimonas mucosa	0	0	0	0.7970303567388294	0.8988457146339978
Scopulariopsis brevicaulis	0	0	0	0.7970303567388294	0.8988457146339978
Lactobacillus amylovorus	0	0	0	0.7970303567388294	0.8988457146339978
Tannerella forsythia	0	0	0	0.7970303567388294	0.8988457146339978
Limosilactobacillus oris	6.489257775382315e-7	2.014444371924447e-7	0.45675653059466426	0.8024735243671983	0.9044752209403067
Streptococcus equinus	4.3163019704120374e-7	2.125796110643436e-7	0.2395794100764018	0.8064512980421437	0.9084476679462934
Cryptobacterium curtum	4.710340333330984e-7	7.095870772952545e-7	-0.21681728320289212	0.8093842794488877	0.9089845604681628
Coprobacter secundus	1.379657867507698e-5	9.93657715816292e-6	0.4361023429190148	0.8129664333310503	0.9089845604681628
Bacteroides helcogenes	1.3852069220452746e-6	1.940936812532403e-6	-0.3021613588609976	0.8129664333310503	0.9089845604681628
Anaerofustis stercorihominis	1.3230339122904517e-6	1.7907570880020782e-6	-0.26464634020461436	0.8129664333310503	0.9089845604681628
Paraprevotella xylaniphila	8.725521292514697e-6	8.388620091134244e-6	0.05086245047177847	0.8129664333310503	0.9089845604681628
Cutibacterium acnes	9.65372923875538e-7	5.091130000944683e-7	0.38110224897727135	0.8139261697348091	0.9089845604681628
Brigitvirus brigit	2.558667451732219e-6	2.0871514345794424e-6	0.2050608695657536	0.8139261697348091	0.9089845604681628
Butyricimonas faecalis	1.1110037404445527e-5	7.74457785771999e-6	0.4697426759088167	0.8144949690877508	0.9089845604681628
Bacteroides faecichinchillae	2.2758079520180803e-6	7.184739872041281e-6	-1.3210857857823446	0.8147774199066066	0.9089845604681628
Schaalia georgiae	3.3157220882223004e-6	6.187458674532419e-6	-0.7358797907729879	0.8147774199066066	0.9089845604681628
Ruminococcus bicirculans (ex Wegman et al 2014)	3.157698173210821e-5	8.700512900000768e-4	-4.740832822344435	0.8200818789054083	0.9089845604681628
Ruthenibacterium lactatiformans	0.0028677418078804128	0.008053421765112641	-1.4893628914469617	0.8200818789054083	0.9089845604681628
Pauljensenia hongkongensis	6.191569277568174e-6	1.7093270272567158e-5	-1.331074670131	0.8200818789054083	0.9089845604681628
Bifidobacterium pseudocatenulatum	0.0010088869221793998	0.001984347958297586	-0.9751981184377806	0.8200818789054083	0.9089845604681628
Bacteroides xylanisolvens	1.7815550314790538e-4	3.196198092128595e-4	-0.839651204169233	0.8200818789054083	0.9089845604681628
Fusicatenibacter saccharivorans	0.01140140943037676	0.007117211263109503	0.6797520541884383	0.8200818789054083	0.9089845604681628
Clostridium sp M62/1	2.9240795167938537e-4	4.148998549325271e-4	-0.5033282115974358	0.8200818789054083	0.9089845604681628
Ligilactobacillus ruminis	4.743690567905756e-6	6.390346471486197e-6	-0.3636639734726852	0.8200818789054083	0.9089845604681628
Agathobacter rectalis	0.003308002104168467	0.00400576353904493	-0.2760411619340534	0.8200818789054083	0.9089845604681628
Parabacteroides merdae	0.0033454212177111624	0.0028252257100599334	0.24374236259743154	0.8200818789054083	0.9089845604681628
Coprococcus eutactus	3.9077722026216924e-5	4.5611532370580414e-5	-0.21788643310628136	0.8200818789054083	0.9089845604681628

Species	Median g1 (resp.)	Median g2 (non-resp.)	log2FC	p	padj
Agathobaculum desmolans	2.8956653897049868e-5	2.4904360686251954e-5	0.20968150778167743	0.8200818789054083	0.9089845604681628
Enterocloster asparagiformis	2.0230638807216929e-4	1.805466346176807e-4	0.1633153599570536	0.8200818789054083	0.9089845604681628
Maliibacterium massiliense	6.442792374545118e-5	6.837270435140107e-5	-0.08446163644302036	0.8200818789054083	0.9089845604681628
Phocaeicola salanitronis	2.057109822905714e-5	1.943575394832759e-5	0.07800415840554405	0.8200818789054083	0.9089845604681628
Escherichia coli	4.4644270887527285e-4	4.6702356893196616e-4	-0.06487821377226771	0.8200818789054083	0.9089845604681628
Monoglobus pectinilyticus	1.1657765843664493e-5	1.1143137104225274e-5	0.05988160357760068	0.8200818789054083	0.9089845604681628
Blautia hansenii	2.8164933204629756e-5	2.928772188799152e-5	-0.05449829890167197	0.8200818789054083	0.9089845604681628
Blautia wexlerae	0.01391350869206117	0.014375303115734215	-0.047102763248924845	0.8200818789054083	0.9089845604681628
Prevotella jejuni	4.599172884748407e-7	0	0.5458866356208033	0.8370707097238814	0.9267898907661097
Clostridium botulinum	2.41075389095678e-7	0	0.3115907546068575	0.8370707097238814	0.9267898907661097
Alistipes sp dk3624	3.84030935876492e-5	9.752439447393759e-7	4.318206158188963	0.8446060147311816	0.9346164845506835
Streptococcus ilei	5.077623071387707e-6	6.275169076731367e-6	-0.25947357399007137	0.8506483386103296	0.9407832619067885
Alistipes finegoldii	6.656793720310337e-4	0.0011272957698107637	-0.7590803093258851	0.851336131279971	0.9410246040182361
Granulicoccus phenolivorans	0	0	0	0.8588828517303749	0.9483202395965131
Plectosphaerella cucumerina	0	0	0	0.8588828517303749	0.9483202395965131
Prevotella veroralis	0	0	0	0.8671053804448808	0.9552936681756686
Leuconostoc falkenbergense	0	0	0	0.8671053804448808	0.9552936681756686
Streptococcus periodonticum	0	0	0	0.8671053804448808	0.9552936681756686
Klebsiella michiganensis	0	0	0	0.8671053804448808	0.9552936681756686
Bifidobacterium biavatii	2.527278177003487e-7	0	0.3250729919067554	0.8730435405590365	0.9586735645371557
Amygdalobacter nucleatus	2.41075389095678e-7	0	0.3115907546068575	0.8730435405590365	0.9586735645371557
Fusobacterium nucleatum	9.270368250546164e-8	0	0.1279022265007203	0.8730435405590365	0.9586735645371557
Streptococcus toyakuensis	9.270368250546164e-8	0	0.1279022265007203	0.8730435405590365	0.9586735645371557
Salmonella enterica	0	0	0	0.8730435405590365	0.9586735645371557
Lughvirus lugh	0	0	0	0.8730435405590365	0.9586735645371557
Bifidobacterium boum	9.270368250546164e-8	1.3051490741272467e-7	-0.04907778965648909	0.8806949402371275	0.9616807557667432
Butyricimonas synergistica	2.41075389095678e-7	2.1697348887728803e-7	0.028293014560032935	0.8806949402371275	0.9616807557667432
Bacteroides oleiciplenus	3.22662326742882e-7	4.28562122651051e-7	-0.11111898363962806	0.8831288805964441	0.9616807557667432
Parabacteroides gordonii	1.2358938158138602e-6	7.383581297159625e-7	0.3631263440555172	0.8849222703005275	0.9616807557667432
Treponema denticola	7.009926775705188e-7	1.0866472763795958e-6	-0.2948098196700157	0.8849222703005275	0.9616807557667432
Streptococcus sp Marseille-Q3533	1.0483903303638191e-6	1.0447132027198064e-6	0.002592152971953447	0.8849222703005275	0.9616807557667432
Actinomyces sp oral taxon 897	2.114094777569814e-6	1.166539979753864e-6	0.5234199942518749	0.8862144186644128	0.9616807557667432
Abiotrophia defectiva	7.354142796381178e-7	1.2188162542240796e-6	-0.3545100939033839	0.8862144186644128	0.9616807557667432
Lactobacillus gasseri	7.246134760044993e-5	5.460258844441541e-6	3.5073214856793817	0.8871111814326641	0.9616807557667432

Species	Median g1 (resp.)	Median g2 (non-resp.)	log2FC	p	padj
Eubacterium limosum	2.982418314793357e-6	2.321049868257902e-6	0.2620053840354334	0.8871111814326641	0.9616807557667432
Streptococcus rubneri	1.2615403565488381e-6	9.053616790739124e-7	0.24724086208433738	0.8871111814326641	0.9616807557667432
Fannyhessea vaginae	1.7691939052083758e-6	1.6406591473419569e-6	0.06856798557835067	0.8871111814326641	0.9616807557667432
Varibaculum cambriense	1.410590151861311e-6	2.515847052397863e-6	-0.5444859262068218	0.8876973768173103	0.9616807557667432
Acinetobacter baumannii	2.6546891865040814e-5	3.29987556526204e-5	-0.30359239729639775	0.8880447410102551	0.9616807557667432
Komagataeibacter oboediens	1.704143528181503e-5	1.9773232466811644e-5	-0.20341161114686807	0.8880447410102551	0.9616807557667432
Ruminococcus bovis	2.5493264140588864e-6	3.048798742390684e-6	-0.18994867328632578	0.8880447410102551	0.9616807557667432
Clostridium sp SY8519	5.3487120581240656e-5	4.7130219584906916e-5	0.17897224713080184	0.8880447410102551	0.9616807557667432
Streptococcus koreensis	1.2151653297106945e-6	1.434455368671719e-6	-0.13618467275560014	0.8880447410102551	0.9616807557667432
Clostridium sp MD294	8.189673698728117e-7	7.095870772952545e-7	0.0894717551019544	0.8880447410102551	0.9616807557667432
Actinomyces dentalis	3.001627019280519e-5	1.9987865056550918e-5	0.5634697888616795	0.8882172187345627	0.9616807557667432
Bifidobacterium pullorum	7.6218520886329645e-6	1.1616677636522133e-5	-0.5492623360510143	0.8882172187345627	0.9616807557667432
Faecalibacterium sp IP-1-18	7.034669139021924e-5	3.1927409812813805e-5	1.1155574992781623	0.8916181857358327	0.9616807557667432
Butyrivibrio crossotus	1.0072346591927524e-4	1.5327244076131084e-4	-0.6008278383454676	0.8916181857358327	0.9616807557667432
Actinomyces pacaensis	2.124654723185528e-5	3.2391037346933735e-5	-0.5858794733295996	0.8916181857358327	0.9616807557667432
Amedibacterium intestinale	6.044965360953577e-6	3.920252361886147e-6	0.517860299116402	0.8916181857358327	0.9616807557667432
Blautia coccoides	1.3867712121180757e-5	1.0357374914367886e-5	0.38855324292383636	0.8916181857358327	0.9616807557667432
Rothia mucilaginosa	7.569343178487697e-5	9.487242020807742e-5	-0.32201282519603036	0.8916181857358327	0.9616807557667432
Coprococcus sp ART55/1	1.1661633885915975e-4	1.4068503351405038e-4	-0.26859888306220986	0.8916181857358327	0.9616807557667432
Blautia argi	3.360235880334492e-5	4.054496469404235e-5	-0.26380324290917245	0.8916181857358327	0.9616807557667432
Propionibacterium acidifaciens	1.2024549969495404e-5	1.450475402539766e-5	-0.2514771138762846	0.8916181857358327	0.9616807557667432
Eubacterium callanderi	1.7662019325396234e-4	1.5210757447004094e-4	0.21424995090498192	0.8916181857358327	0.9616807557667432
Lactococcus cremoris	6.626014699068513e-6	6.070710667730384e-6	0.10907408616419557	0.8916181857358327	0.9616807557667432
Catenibacterium sp co 0103	1.540966672655173e-5	1.4818055908818475e-5	0.052973639885366124	0.8916181857358327	0.9616807557667432
Enterobacter asburiae	0	0	0	0.8976772276808522	0.9666551124516001
Lacrimispora xylanolytica	0	0	0	0.8976772276808522	0.9666551124516001
Aspergillus nomiae	0	0	0	0.8976772276808522	0.9666551124516001
Segatella baroniae	1.0109112708013948e-6	0	1.007849425751222	0.9181051103274143	0.9796918229167008
Segatella salivae	4.82150778191356e-7	0	0.5676922194953893	0.9181051103274143	0.9796918229167008
Hallella bergensis	3.8110961589105143e-7	0	0.4658278279424796	0.9181051103274143	0.9796918229167008
Ruminococcus flavefaciens	2.41075389095678e-7	0	0.3115907546068575	0.9181051103274143	0.9796918229167008
Filifactor alocis	1.8540736501092327e-7	0	0.2453829262015322	0.9181051103274143	0.9796918229167008
Berryella intestinalis	1.5163669062020923e-6	4.737579117442952e-7	0.7718427356929977	0.9219301876171699	0.9796918229167008
Lactobacillus paragasseri	1.297851555076463e-6	6.653822654925909e-7	0.4644322345390753	0.9219301876171699	0.9796918229167008

Species	Median g1 (resp.)	Median g2 (non-resp.)	log2FC	p	padj
Streptococcus equi	2.831197760862315e-7	3.691048219307301e-7	-0.09357705823811323	0.9219301876171699	0.9796918229167008
Gallibacterium salpingitidis	1.1433288476731543e-6	6.301346367561794e-7	0.3948620924500596	0.9239992707820663	0.9796918229167008
Desulfovibrio piger	5.046949010468958e-6	3.3827595155601474e-6	0.4643678942817575	0.9249934749771813	0.9796918229167008
Bradyrhizobium sp SK17	9.137809752325597e-7	5.22208115505401e-7	0.33026011160624	0.9249934749771813	0.9796918229167008
Intestinibacter bartlettii	7.552073348643073e-5	6.74150002977411e-5	0.16153802545051235	0.9249934749771813	0.9796918229167008
Streptococcus oralis	3.362956075315675e-5	9.754592744024533e-6	1.6870511946006912	0.9253419524207791	0.9796918229167008
Bifidobacterium bifidum	2.1702857172781993e-5	2.6744207257323147e-5	-0.28931270779549423	0.9253419524207791	0.9796918229167008
Gemella morbillorum	3.996226095191926e-6	4.087353364882701e-6	-0.026076541535277168	0.9253419524207791	0.9796918229167008
Amygdalobacter indicium	0	0	0	0.9425378416084825	0.9796918229167008
Anaerococcus obesiensis	0	0	0	0.9425378416084825	0.9796918229167008
Anaerostipes rhamnosivorans	0	0	0	0.9425378416084825	0.9796918229167008
Campylobacter showae	0	0	0	0.9425378416084825	0.9796918229167008
Clostridium chauvoei	0	0	0	0.9425378416084825	0.9796918229167008
Enterococcus avium	0	0	0	0.9425378416084825	0.9796918229167008
Escherichia albertii	0	0	0	0.9425378416084825	0.9796918229167008
Escherichia fergusonii	0	0	0	0.9425378416084825	0.9796918229167008
Haemophilus paraphrohaemolyticus	0	0	0	0.9425378416084825	0.9796918229167008
Kineothrix alysoides	0	0	0	0.9425378416084825	0.9796918229167008
Lactococcus garvieae	0	0	0	0.9425378416084825	0.9796918229167008
Neisseria subflava	0	0	0	0.9425378416084825	0.9796918229167008
Streptococcus alactolyticus	0	0	0	0.9425378416084825	0.9796918229167008
Streptococcus parauberis	0	0	0	0.9425378416084825	0.9796918229167008
Ancylostoma duodenale	0	0	0	0.9425378416084825	0.9796918229167008
Culoivirus americanus	0	0	0	0.9425378416084825	0.9796918229167008
Dolosigranulum pigrum	0	0	0	0.9425378416084825	0.9796918229167008
Enterobacter kobei	0	0	0	0.9425378416084825	0.9796918229167008
Ligilactobacillus agilis	0	0	0	0.9425378416084825	0.9796918229167008
Porphyromonas asaccharolytica	0	0	0	0.9425378416084825	0.9796918229167008
Porphyromonas uenonis	0	0	0	0.9425378416084825	0.9796918229167008
Varibaculum prostatecancerukia	0	0	0	0.9425378416084825	0.9796918229167008
Leptotrichia sp oral taxon 212	0	0	0	0.9425378416084825	0.9796918229167008
Brevibacterium sp CS2	0	0	0	0.9425378416084825	0.9796918229167008
Dermabacter jinjuensis	0	0	0	0.9425378416084825	0.9796918229167008
Agrococcus sp REN33	0	0	0	0.9425378416084825	0.9796918229167008

Species	Median g1 (resp.)	Median g2 (non-resp.)	log2FC	p	padj
Anaerocolumna sp AGMB13025	0	0	0	0.9425378416084825	0.9796918229167008
Lactobacillus kefiranofaciens	0	0	0	0.9425378416084825	0.9796918229167008
Leuconostoc gelidum	0	0	0	0.9425378416084825	0.9796918229167008
Limosilactobacillus portuensis	0	0	0	0.9425378416084825	0.9796918229167008
Macrococcoides caseolyticum	0	0	0	0.9425378416084825	0.9796918229167008
Streptococcus ruminicola	0	0	0	0.9425378416084825	0.9796918229167008
Debaryomyces hansenii	0	0	0	0.9425378416084825	0.9796918229167008
Parascedosporium putredinis	0	0	0	0.9425378416084825	0.9796918229167008
Staphylococcus haemolyticus	0	0	0	0.9425378416084825	0.9796918229167008
Actinomyces radicidentis	0	0	0	0.9425378416084825	0.9796918229167008
Corynebacterium striatum	0	0	0	0.9425378416084825	0.9796918229167008
Bacteroides reticulotermitis	0	0	0	0.9425378416084825	0.9796918229167008
Geotrichum candidum	0	0	0	0.9425378416084825	0.9796918229167008
Segatella albensis	0	0	0	0.9425378416084825	0.9796918229167008
Fusobacterium canifelinum	0	0	0	0.9425378416084825	0.9796918229167008
Moineauvirus Abc2	0	0	0	0.9425378416084825	0.9796918229167008
Providencia rettgeri	0	0	0	0.9425378416084825	0.9796918229167008
Selenomonas sp oral taxon 136	0	0	0	0.9425378416084825	0.9796918229167008
Centipeda periodontii	0	0	0	0.9425378416084825	0.9796918229167008
Hoylesella pleuritidis	0	0	0	0.9425378416084825	0.9796918229167008
Jonquetella anthropi	0	0	0	0.9425378416084825	0.9796918229167008
Prevotella sp oral taxon 475	0	0	0	0.9425378416084825	0.9796918229167008
Selenomonas flueggei	0	0	0	0.9425378416084825	0.9796918229167008
Selenomonas sp oral taxon 126	0	0	0	0.9425378416084825	0.9796918229167008
Kehishuvirus primarius	0	0	0	0.9425378416084825	0.9796918229167008
Kehishuvirus splanchnicus	0	0	0	0.9425378416084825	0.9796918229167008
Peptoniphilus equinus	0	0	0	0.9527406585935637	0.9877366803498894
Exophiala spinifera	0	0	0	0.9555180290603862	0.9877366803498894
Duodenibacillus massiliensis	0	0	0	0.9555180290603862	0.9877366803498894
Bulleidia sp zg-1006	0	0	0	0.9555180290603862	0.9877366803498894
Enterobacter hormaechei	0	0	0	0.9555180290603862	0.9877366803498894
Actinotignum urinale	0	0	0	0.9575210961581013	0.9877366803498894
Corynebacterium propinquum	0	0	0	0.9575210961581013	0.9877366803498894
Lawsonella clevelandensis	0	0	0	0.9575210961581013	0.9877366803498894

Species	Median g1 (resp.)	Median g2 (non-resp.)	log2FC	p	padj
Dielma fastidiosa	0	0	0	0.9575210961581013	0.9877366803498894
Junduvirus communis	9.059832834759408e-6	7.157697435540163e-7	2.5516784707933753	0.9600989297624472	0.9877366803498894
Hoylesella timonensis	2.081418237993545e-6	1.166539979753864e-6	0.5082016520047542	0.9615217159265494	0.9877366803498894
Parafannyhessea umbonata	6.675581432022794e-7	8.368358521394655e-7	-0.13948564140705844	0.9615217159265494	0.9877366803498894
Lachnobacterium bovis	1.4265873381241416e-6	1.6153947011641897e-6	-0.10809989985309779	0.9619564151388587	0.9877366803498894
Parabacteroides sp CT06	1.668326698131506e-6	2.775736427234378e-6	-0.500822743484371	0.962258047126084	0.9877366803498894
Streptococcus constellatus	1.4708285592762356e-6	4.866730997870243e-7	0.7329074591535011	0.9624551943783576	0.9877366803498894
uncultured Alistipes sp	7.575706512806977e-6	7.643106739546363e-6	-0.011294444986290172	0.9625720100595966	0.9877366803498894
Streptococcus agalactiae	4.225996833520876e-6	5.399550940708461e-6	-0.29226442360548655	0.9626300104906452	0.9877366803498894
Homo sapiens	0.007402485041006476	0.0010645557913929998	2.796598417065537	0.9635854341736694	0.9877366803498894
Enterococcus faecium	4.1454150185498776e-5	5.605747273803915e-5	-0.4265102526097832	0.9635854341736694	0.9877366803498894
Bacteroides finegoldii	1.0331854448861133e-4	1.2518687988997108e-4	-0.2745662703299132	0.9635854341736694	0.9877366803498894
Faecalibacterium prausnitzii	0.003100941658447976	0.0036076292790597573	-0.21827938789330562	0.9635854341736694	0.9877366803498894
Ruminococcus callidus	4.446531340234866e-5	5.081263865840779e-5	-0.18853775979218923	0.9635854341736694	0.9877366803498894
Faecalibacterium sp IP-3-29	1.0250455356483008e-4	1.1629834800424627e-4	-0.18048845616862177	0.9635854341736694	0.9877366803498894
Qiania dongpingensis	1.142509087226596e-5	1.1291520581584756e-5	0.015593000864901799	0.9635854341736694	0.9877366803498894
Faecalibacterium sp I2-3-92	2.9620616765068373e-4	1.7545913768489958e-4	0.7521299151738562	0.9635854341736696	0.9877366803498894
Flintibacter sp KGMB00164	1.4908525634348639e-5	1.7372230169214365e-5	-0.207726626820118	0.9635854341736696	0.9877366803498894
[Clostridium] innocuum	5.515934651141212e-4	6.278350531501501e-4	-0.18646313882242507	0.9635854341736696	0.9877366803498894
Clostridium perfringens	4.2821561437711936e-5	5.636496251315934e-6	2.7231472164026673	1	1
Alistipes onderdonkii	0.007176361445781839	0.003329051064335435	1.1079092750980242	1	1
Bifidobacterium moukalabense	2.1355067236926774e-6	5.008400038897148e-7	1.0629284257084313	1	1
Bacteroides cellulosilyticus	1.3734135687633255e-4	2.4975968460333144e-4	-0.858072908505818	1	1
Adlercreutzia hattorii	3.377336486655481e-4	2.3945563816586942e-4	0.4943805517455242	1	1
Bacteroides sp CACC 737	1.9109353805659815e-5	1.3382438433244887e-5	0.4835584282938161	1	1
Alistipes shahii	5.875026248188623e-4	8.05465013668707e-4	-0.45456309733010863	1	1
Marvinbryantia formatexigens	2.7210328346610357e-5	3.6958039518165196e-5	-0.4281820231329253	1	1
Lactococcus lactis	4.923540348976054e-6	6.699253879678304e-6	-0.3782589480376343	1	1
Streptococcus lutetiensis	2.831197760862315e-7	0	0.3596558487597289	1	1
Fusobacterium animalis	2.7811104751638493e-7	0	0.35401318897758316	1	1
Aeromonas salmonicida	6.342284332709441e-6	4.848388098763623e-6	0.3281899327815994	1	1
Streptococcus pseudopneumoniae	2.527278177003487e-7	0	0.3250729919067554	1	1
Junduvirus copri	2.41075389095678e-7	0	0.3115907546068575	1	1
Dorea formicigenerans	0.004454276871537525	0.005506195017900206	-0.30580260706893475	1	1

Species	Median g1 (resp.)	Median g2 (non-resp.)	log2FC	p	padj
Phocaeicola massiliensis	1.9393186831418736e-5	2.338679113957259e-5	-0.2580126959760775	1	1
Stomatobaculum sp F0698	1.9055480794552572e-7	0	0.2516340375180648	1	1
Bacteroides nordii	1.1607910819535492e-5	1.3455105706500169e-5	-0.19724992369241162	1	1
Granulicatella adiacens	1.4189398256673642e-5	1.2309595164596125e-5	0.19059802741469384	1	1
Longicatena caecimuris	5.9538886671018645e-6	6.384627290066418e-6	-0.08670513901987266	1	1
Holdemania filiformis	1.3476513339370555e-4	1.393807979771849e-4	-0.04823258919764698	1	1
Alistipes timonensis	1.536844292282348e-5	1.4912539941778034e-5	0.040752953999731494	1	1
Staphylococcus epidermidis	2.2995864423742035e-7	2.014444371924447e-7	0.0338398774670196	1	1
Haemophilus parahaemolyticus	0	0	0	1	1
Haemophilus pittmaniae	0	0	0	1	1
Megasphaera massiliensis	0	0	0	1	1
Aristaeella lactis	0	0	0	1	1
Kocuria varians	0	0	0	1	1
Pseudomonas aeruginosa	0	0	0	1	1
Desulfovibrio fairfieldensis	0	0	0	1	1
Dabirmavirus hominis	0	0	0	1	1
Neisseria sp DTU 2021 1001991 1 SI NGA ILE 055	0	0	0	1	1
Rothia kristinae	0	0	0	1	1
Lactobacillus acetotolerans	0	0	0	1	1
Aggregatibacter actinomycetemcomitans	0	0	0	1	1
Dialister massiliensis	0	0	0	1	1
Enterobacter ludwigii	0	0	0	1	1
Sinanaerobacter sp ZZT-01	0	0	0	1	1
Caproicibacterium lactatifermentans	0	0	0	1	1
Penicillium citrinum	0	0	0	1	1
Rickettsiella massiliensis	0	0	0	1	1
Slackia heliotrinireducens	0	0	0	1	1
Alloprevotella rava	0	0	0	1	1
Cloacibacillus porcorum	0	0	0	1	1
Prevotella sp Rep29	0	0	0	1	1
Carnobacterium maltaromaticum	0	0	0	1	1
Citrobacter amalonaticus	0	0	0	1	1
Histophilus somni	0	0	0	1	1
Hoylesella marshii	0	0	0	1	1

Species	Median g1 (resp.)	Median g2 (non-resp.)	log2FC	p	padj
Agromyces soli	0	0	0	nan	nan
Anaeroarcus burkinensis	0	0	0	nan	nan
Bifidobacterium stellenboschense	0	0	0	nan	nan
Candidatus Methanomassiliicoccus intestinalis	0	0	0	nan	nan
Clostridium argentinense	0	0	0	nan	nan
Clostridium sp JN-1	0	0	0	nan	nan
Clostridium taeniosporum	0	0	0	nan	nan
Corynebacterium argentoratense	0	0	0	nan	nan
Corynebacterium kutscheri	0	0	0	nan	nan
Corynebacterium phocae	0	0	0	nan	nan
Cupriavidus sp EM10	0	0	0	nan	nan
Dickeya fangzhongdai	0	0	0	nan	nan
Erwinia oleae	0	0	0	nan	nan
Escherichia sp E4742	0	0	0	nan	nan
Haloechinothrix halophila	0	0	0	nan	nan
Human mastadenovirus C	0	0	0	nan	nan
Limnoglobus roseus	0	0	0	nan	nan
Paracoccus yeei	0	0	0	nan	nan
Pseudomonas tremae	0	0	0	nan	nan
Sedimentibacter sp MB31-C6	0	0	0	nan	nan
Sphingobium yanoikuyae	0	0	0	nan	nan
Spirosoma foliorum	0	0	0	nan	nan
Streptococcus oriscaviae	0	0	0	nan	nan
[Clostridium] colinum	0	0	0	nan	nan
[Haemophilus] ducreyi	0	0	0	nan	nan
Actinomyces urogenitalis	0	0	0	nan	nan
Anoxybacillus flavithermus	0	0	0	nan	nan
Bifidobacterium sp ESL0732	0	0	0	nan	nan
Blohavirus faecalis	0	0	0	nan	nan
Brevefilum fermentans	0	0	0	nan	nan
Caldicellulosiruptor diazotrophicus	0	0	0	nan	nan
Clostridium sp DL-VIII	0	0	0	nan	nan
Companilactobacillus alimentarius	0	0	0	nan	nan
Companilactobacillus heilongjiangensis	0	0	0	nan	nan

Species	Median g1 (resp.)	Median g2 (non-resp.)	log2FC	p	padj
Corynebacterium silvaticum	0	0	0	nan	nan
Devosia sp RR2S18	0	0	0	nan	nan
Flavobacterium sp GSB-24	0	0	0	nan	nan
Geobacillus stearothermophilus	0	0	0	nan	nan
Gleimia hominis	0	0	0	nan	nan
Halorientalis sp IM1011	0	0	0	nan	nan
Heyndrickxia ginsengihumi	0	0	0	nan	nan
Leeuwenhoekiella blandensis	0	0	0	nan	nan
Limnohabitans sp 103DPR2	0	0	0	nan	nan
Lysinibacillus irui	0	0	0	nan	nan
Metallosphaera hakonensis	0	0	0	nan	nan
Methanobrevibacter sp AbM4	0	0	0	nan	nan
Micromonospora rifamycinica	0	0	0	nan	nan
Mobiluncus mulieris	0	0	0	nan	nan
Mucor irregularis	0	0	0	nan	nan
Naganishia albida	0	0	0	nan	nan
Neobacillus drentensis	0	0	0	nan	nan
Obesumbacterium proteus	0	0	0	nan	nan
Ornithobacterium rhinotracheale	0	0	0	nan	nan
Paenibacillus odorifer	0	0	0	nan	nan
Pantoea sp MT58	0	0	0	nan	nan
Paraburkholderia fungorum	0	0	0	nan	nan
Paracidovorax citrulli	0	0	0	nan	nan
Phaeobacter inhibens	0	0	0	nan	nan
Prolixibacter bellariivorans	0	0	0	nan	nan
Pseudomonas denitrificans (nom rej)	0	0	0	nan	nan
Pseudomonas saxonica	0	0	0	nan	nan
Rhizobium tumorigenes	0	0	0	nan	nan
Ruminiclostridium papyrosolvens	0	0	0	nan	nan
Sporomusa ovata	0	0	0	nan	nan
Staphylococcus shinii	0	0	0	nan	nan
Streptococcus merionis	0	0	0	nan	nan
Tepidimonas taiwanensis	0	0	0	nan	nan
Thermoanaerobacterium xylanolyticum	0	0	0	nan	nan

Species	Median g1 (resp.)	Median g2 (non-resp.)	log2FC	p	padj
Thermosynechococcus vestitus	0	0	0	nan	nan
Thermothielavioides terrestris	0	0	0	nan	nan
uncultured Methanobacterium sp	0	0	0	nan	nan
Actinoplanes sichuanensis	0	0	0	nan	nan
Alkalitalea saponilacus	0	0	0	nan	nan
Campylobacter fetus	0	0	0	nan	nan
Candidatus Haliotispira prima	0	0	0	nan	nan
Catellatospora sp IY07-71	0	0	0	nan	nan
Caulobacter sp FWC26	0	0	0	nan	nan
Caulobacter sp NIBR2454	0	0	0	nan	nan
Caulobacter vibrioides	0	0	0	nan	nan
Chlamydoabsidia padenii	0	0	0	nan	nan
Cohnella herbarum	0	0	0	nan	nan
Comamonas sp B21-038	0	0	0	nan	nan
Dechloromonas sp HYN0024	0	0	0	nan	nan
Dehalobacter sp CF	0	0	0	nan	nan
Desulfomicrobium escambiense	0	0	0	nan	nan
Endomicrobium proavitum	0	0	0	nan	nan
Geminisphaera colitermitum	0	0	0	nan	nan
Halarcobacter bivalviorum	0	0	0	nan	nan
Methylobacter sp S3L5C	0	0	0	nan	nan
Micromonospora chokoriensis	0	0	0	nan	nan
Moraxella osloensis	0	0	0	nan	nan
Naasia aerilata	0	0	0	nan	nan
Neptunomonas japonica	0	0	0	nan	nan
Nocardioides ochotonae	0	0	0	nan	nan
Nocardioides seonyuensis	0	0	0	nan	nan
Paenibacillus sp RUD330	0	0	0	nan	nan
Paraburkholderia sp SOS3	0	0	0	nan	nan
Paracoccus contaminans	0	0	0	nan	nan
Pelodictyon luteolum	0	0	0	nan	nan
Phytobacter ursingii	0	0	0	nan	nan
Planococcus sp 107-1	0	0	0	nan	nan
Polynucleobacter sp MWH-UH25E	0	0	0	nan	nan

Species	Median g1 (resp.)	Median g2 (non-resp.)	log2FC	p	padj
Pseudoglutamicibacter albus	0	0	0	nan	nan
Pseudomonas canavaninivorans	0	0	0	nan	nan
Pseudomonas sp PIA16	0	0	0	nan	nan
Streptomyces anulatus	0	0	0	nan	nan
Streptomyces collinus	0	0	0	nan	nan
Streptomyces phaeolivaceus	0	0	0	nan	nan
Streptomyces sp ST1015	0	0	0	nan	nan
Streptomyces spectabilis	0	0	0	nan	nan
Streptosporangium roseum	0	0	0	nan	nan
Sulfurospirillum halorespirans	0	0	0	nan	nan
Synechococcus sp Minos11	0	0	0	nan	nan
Synechococcus sp WH 8101	0	0	0	nan	nan
Thermochromatium tepidum	0	0	0	nan	nan
Trichlorobacter ammonificans	0	0	0	nan	nan
Trichocoleus desertorum	0	0	0	nan	nan
iashengella succiniciproducens	0	0	0	nan	nan
[Enterobacter] lignolyticus	0	0	0	nan	nan
Alternaria alternata	0	0	0	nan	nan
Aneurinibacillus sp B1	0	0	0	nan	nan
Azospirillum sp TSH100	0	0	0	nan	nan
Candidatus Enterovibrio luxaltus	0	0	0	nan	nan
Chryseobacterium gambrini	0	0	0	nan	nan
Clonorchis sinensis	0	0	0	nan	nan
Clostridium sp JN-9	0	0	0	nan	nan
Dietzia alimentaria	0	0	0	nan	nan
Fervidibacillus albus	0	0	0	nan	nan
Listeria rocourtiae	0	0	0	nan	nan
Meyerozyma guilliermondii	0	0	0	nan	nan
Mycolicibacterium thermoresistibile	0	0	0	nan	nan
Neobacillus sp PS3-34	0	0	0	nan	nan
Paecilomyces variotii	0	0	0	nan	nan
Paenibacillus algicola	0	0	0	nan	nan
Pseudooceanicola atlanticus	0	0	0	nan	nan
Schaalia vaccimaxillae	0	0	0	nan	nan

Species	Median g1 (resp.)	Median g2 (non-resp.)	log2FC	p	padj
Sporosarcina sp PTS2304	0	0	0	nan	nan
Acinetobacter schindleri	0	0	0	nan	nan
Aerococcus urinaeequi	0	0	0	nan	nan
Chlamydia suis	0	0	0	nan	nan
Coraliomargarita akajimensis	0	0	0	nan	nan
Corynebacterium ciconiae	0	0	0	nan	nan
Egibacter rhizosphaerae	0	0	0	nan	nan
Methanosarcina mazei	0	0	0	nan	nan
Novosphingobium decolorationis	0	0	0	nan	nan
Pseudonocardia sp HH130629-09	0	0	0	nan	nan
Riemerella anatipestifer	0	0	0	nan	nan
Shigella boydii	0	0	0	nan	nan
Sinomonas atrocyanea	0	0	0	nan	nan
Stenotrophomonas sp PAMC25021	0	0	0	nan	nan
Streptomyces flavovariabilis	0	0	0	nan	nan
Streptomyces olivoreticuli	0	0	0	nan	nan
Trichosporon sp PB005A	0	0	0	nan	nan
Trueperella bernardiae	0	0	0	nan	nan
Aerococcus sp Group 1	0	0	0	nan	nan
Alloalcanivorax xenomutans	0	0	0	nan	nan
Arachidicoccus sp BS20	0	0	0	nan	nan
Arcobacter roscoffensis	0	0	0	nan	nan
Arthrobacter sp CJ23	0	0	0	nan	nan
Bryobacter aggregatus	0	0	0	nan	nan
Candidatus Epulopiscium viviparus	0	0	0	nan	nan
Capillimicrobium parvum	0	0	0	nan	nan
Chlamydia pneumoniae	0	0	0	nan	nan
Exiguobacterium sp MH3	0	0	0	nan	nan
Flavobacterium subsaxonicum	0	0	0	nan	nan
Globicatella sp PHS-GS-PNBC-21-1553	0	0	0	nan	nan
Gorillibacterium massiliense	0	0	0	nan	nan
Helcococcus ovis	0	0	0	nan	nan
Hydrogenovibrio halophilus	0	0	0	nan	nan
Hymenobacter sublimis	0	0	0	nan	nan

Species	Median g1 (resp.)	Median g2 (non-resp.)	log2FC	p	padj
Idiomarina sp OXR-189	0	0	0	nan	nan
Magnetospirillum fulvum	0	0	0	nan	nan
Maribacter sp BPC-D8	0	0	0	nan	nan
Mesorhizobium australicum	0	0	0	nan	nan
Microsporum audouinii	0	0	0	nan	nan
Paenibacillus lutimineralis	0	0	0	nan	nan
Phialophora macrospora	0	0	0	nan	nan
Planktothrix tepida	0	0	0	nan	nan
Polynucleobacter sp KF022	0	0	0	nan	nan
Pseudocitrobacter corydidari	0	0	0	nan	nan
Rhodocytophaga rosea	0	0	0	nan	nan
Saccharomycetales sp PK3305	0	0	0	nan	nan
Saccharospirillum impatiens	0	0	0	nan	nan
Salicibibacter kimchii	0	0	0	nan	nan
Secundilactobacillus oryzae	0	0	0	nan	nan
Solitalea lacus	0	0	0	nan	nan
Sphingobacterium psychroaquaticum	0	0	0	nan	nan
Sphingomonas psychrotolerans	0	0	0	nan	nan
Streptomyces cellulosae	0	0	0	nan	nan
Streptomyces coeruleorubidus	0	0	0	nan	nan
Streptomyces sp NBC 01268	0	0	0	nan	nan
Sulfitobacter sp OXR-159	0	0	0	nan	nan
Ureaplasma urealyticum	0	0	0	nan	nan
Vibrio furnissii	0	0	0	nan	nan
[Candida] boidinii	0	0	0	nan	nan
uncultured Sphaerochaeta sp	0	0	0	nan	nan
Abyssalbus ytuae	0	0	0	nan	nan
Acetomicrobium mobile	0	0	0	nan	nan
Achromobacter xylosoxidans	0	0	0	nan	nan
Actinomyces procaprae	0	0	0	nan	nan
Amycolatopsis sp FU40	0	0	0	nan	nan
Aquella oligotrophica	0	0	0	nan	nan
Aspergillus clavatus	0	0	0	nan	nan
Aspergillus terreus	0	0	0	nan	nan

Species	Median g1 (resp.)	Median g2 (non-resp.)	log2FC	p	padj
Brachybacterium halotolerans	0	0	0	nan	nan
Brachybacterium vulturis	0	0	0	nan	nan
Brucella pseudogrignonensis	0	0	0	nan	nan
Brussowvirus P4761	0	0	0	nan	nan
Brussowvirus P7951	0	0	0	nan	nan
Candida dubliniensis	0	0	0	nan	nan
Chaetomiaceae sp PYS4301M	0	0	0	nan	nan
Citrobacter youngae	0	0	0	nan	nan
Corynebacterium glutamicum	0	0	0	nan	nan
Deinococcus gobiensis	0	0	0	nan	nan
Desulfitobacterium hafniense	0	0	0	nan	nan
Dracunculus medinensis	0	0	0	nan	nan
Dyella terrae	0	0	0	nan	nan
Enterobacter sp Colony194	0	0	0	nan	nan
Erwinia billingiae	0	0	0	nan	nan
Fusobacterium russii	0	0	0	nan	nan
Hippea jasoniae	0	0	0	nan	nan
Homoserinibacter sp YIM 151385	0	0	0	nan	nan
Klebsiella electrica	0	0	0	nan	nan
Klebsiella quasivariicola	0	0	0	nan	nan
Kluyveromyces marxianus	0	0	0	nan	nan
Kytococcus sedentarius	0	0	0	nan	nan
Limnobaculum zhutongyuii	0	0	0	nan	nan
Mergibacter septicus	0	0	0	nan	nan
Mesomycoplasma ovipneumoniae	0	0	0	nan	nan
Methanohalobium evestigatum	0	0	0	nan	nan
Methylobacterium oryzae	0	0	0	nan	nan
Micrococcus yunnanensis	0	0	0	nan	nan
Moineauvirus Sfi21	0	0	0	nan	nan
Mydovirus BIS47	0	0	0	nan	nan
Mydovirus KpS8	0	0	0	nan	nan
Orrella marina	0	0	0	nan	nan
Paenibacillus azoreducens	0	0	0	nan	nan
Pseudodesulfovibrio sediminis	0	0	0	nan	nan

Species	Median g1 (resp.)	Median g2 (non-resp.)	log2FC	p	padj
Psychrilyobacter atlanticus	0	0	0	nan	nan
Serratia plymuthica	0	0	0	nan	nan
Serratia symbiotica	0	0	0	nan	nan
Shigella sonnei	0	0	0	nan	nan
Sphingomonas koreensis	0	0	0	nan	nan
Streptococcus phage CHPC1033	0	0	0	nan	nan
Streptococcus phage CHPC1041	0	0	0	nan	nan
Streptococcus phage CHPC640	0	0	0	nan	nan
Streptococcus phage L5A1	0	0	0	nan	nan
Streptococcus phage P7601	0	0	0	nan	nan
Streptococcus phage SW1	0	0	0	nan	nan
Streptococcus phage SW1151	0	0	0	nan	nan
Streptomyces sp NBC 01320	0	0	0	nan	nan
Uliginosibacterium gangwonense	0	0	0	nan	nan
Ureibacillus thermosphaericus	0	0	0	nan	nan
Yersinia aldovae	0	0	0	nan	nan
uncultured Ilyobacter sp	0	0	0	nan	nan
Aeromonas bestiarum	0	0	0	nan	nan
Anaerohalosphaera lusitana	0	0	0	nan	nan
Aspergillaceae sp S1701	0	0	0	nan	nan
Candidatus Annandia adelgestsuga	0	0	0	nan	nan
Cellulophaga sp HaHa 2 95	0	0	0	nan	nan
Citricoccus sp SGAir0253	0	0	0	nan	nan
Corynebacterium pseudokroppenstedtii	0	0	0	nan	nan
Corynespora cassiicola	0	0	0	nan	nan
Devosia rhizoryzae	0	0	0	nan	nan
Enterobacteriaceae endosymbiont of Plateumaris braccata	0	0	0	nan	nan
Falseniella ignava	0	0	0	nan	nan
Flavobacterium sp YJ01	0	0	0	nan	nan
Halopseudomonas maritima	0	0	0	nan	nan
Hamadaea tsunoensis	0	0	0	nan	nan
Herbaspirillum sp WKF16	0	0	0	nan	nan
Hydrogenibacillus sp N12	0	0	0	nan	nan
Hymenobacter sp YIM 151500-1	0	0	0	nan	nan

Species	Median g1 (resp.)	Median g2 (non-resp.)	log2FC	p	padj
Leucobacter sp Psy1	0	0	0	nan	nan
Oceanicola granulosus	0	0	0	nan	nan
Paraflavitalea soli	0	0	0	nan	nan
Paucilactobacillus oligofermentans	0	0	0	nan	nan
Peptoniphilus ivorii	0	0	0	nan	nan
Roseibium aggregatum	0	0	0	nan	nan
Sinorhizobium fredii	0	0	0	nan	nan
Sphingomonas ginsengisoli An et al 2013	0	0	0	nan	nan
Sporothrix schenckii	0	0	0	nan	nan
Streptococcus phage SW6	0	0	0	nan	nan
Streptococcus pluranimalium	0	0	0	nan	nan
Streptomyces ferrugineus	0	0	0	nan	nan
Succinivibrio dextrinosolvens	0	0	0	nan	nan
Terrimonas ferruginea	0	0	0	nan	nan
Yamadazyma nakazawae	0	0	0	nan	nan
Aureimonas altamirensis	0	0	0	nan	nan
Azovibrio restrictus	0	0	0	nan	nan
Bacteroides sp BFG-257	0	0	0	nan	nan
Brevibacterium oceani	0	0	0	nan	nan
Candidatus Viadribacter manganicus	0	0	0	nan	nan
Chlorobaculum limnaeum	0	0	0	nan	nan
Clostridium hydrogeniformans	0	0	0	nan	nan
Crassaminicella profunda	0	0	0	nan	nan
Flavobacterium cauense	0	0	0	nan	nan
Hydrogenophaga pseudoflava	0	0	0	nan	nan
Jannaschia sp W003	0	0	0	nan	nan
Mesobacillus jeotgali	0	0	0	nan	nan
Mesoterricola silvestris	0	0	0	nan	nan
Paludisphaera borealis	0	0	0	nan	nan
Picosynechococcus sp PCC 73109	0	0	0	nan	nan
Pseudomonas lijiangensis	0	0	0	nan	nan
Pseudomonas sp FP453	0	0	0	nan	nan
Roseomonas aerilata	0	0	0	nan	nan
Saccharomonospora viridis	0	0	0	nan	nan

Species	Median g1 (resp.)	Median g2 (non-resp.)	log2FC	p	padj
Saccharopolyspora pogona	0	0	0	nan	nan
Sinorhizobium sp BG8	0	0	0	nan	nan
Spiroplasma chinense	0	0	0	nan	nan
Thermonema rossianum	0	0	0	nan	nan
ylanibacter ruminicola	0	0	0	nan	nan
uncultured Cohaesibacter sp	0	0	0	nan	nan
uncultured Paludibaculum sp	0	0	0	nan	nan
Acinetobacter pittii	0	0	0	nan	nan
Acinetobacter seifertii	0	0	0	nan	nan
Bacteroides sp PHL 2737	0	0	0	nan	nan
Borreliella californiensis	0	0	0	nan	nan
Burkholderia sp S-53	0	0	0	nan	nan
Cedecea neteri	0	0	0	nan	nan
Citrobacter freundii complex sp CFNIH3	0	0	0	nan	nan
Citrobacter sp CF971	0	0	0	nan	nan
Citrobacter sp RHB35-C21	0	0	0	nan	nan
Citrobacter sp RHBSTW-00127	0	0	0	nan	nan
Citrobacter sp RHBSTW-00570	0	0	0	nan	nan
Citrobacter sp RHBSTW-00696	0	0	0	nan	nan
Clostridium novyi	0	0	0	nan	nan
Deinococcus proteolyticus	0	0	0	nan	nan
Efquatrovirus AUEF3	0	0	0	nan	nan
Enterobacter cloacae complex sp ECL411	0	0	0	nan	nan
Enterobacter sp T2	0	0	0	nan	nan
Enterococcus dispar	0	0	0	nan	nan
Exophiala oligosperma	0	0	0	nan	nan
Felixounavirus Alf5	0	0	0	nan	nan
Felixounavirus BPS15Q2	0	0	0	nan	nan
Felixounavirus JH2	0	0	0	nan	nan
Felixounavirus UAB87	0	0	0	nan	nan
Franconibacter helveticus	0	0	0	nan	nan
Gordonia sp w5E2	0	0	0	nan	nan
Granulicella sp 5B5	0	0	0	nan	nan
Hafnia phage yong1	0	0	0	nan	nan

Species	Median g1 (resp.)	Median g2 (non-resp.)	log2FC	p	padj
Hymenobacter sp BRD128	0	0	0	nan	nan
Karamvirus cc31	0	0	0	nan	nan
Karamvirus petcm34	0	0	0	nan	nan
Klebsiella sp P1CD1	0	0	0	nan	nan
Klebsiella sp WP3-S18-ESBL-05	0	0	0	nan	nan
Lactococcus phage 4268	0	0	0	nan	nan
Lecanicillium sp S3808	0	0	0	nan	nan
Leuconostoc sp C2	0	0	0	nan	nan
Ligilactobacillus cholophilus	0	0	0	nan	nan
Macrococcus armenti	0	0	0	nan	nan
Nocardioides sp LMS-CY	0	0	0	nan	nan
Paenisporosarcina antarctica	0	0	0	nan	nan
Pantoea sp Lij88	0	0	0	nan	nan
Paracholeplasma brassicae	0	0	0	nan	nan
Pediococcus damnosus	0	0	0	nan	nan
Pediococcus sp AC40	0	0	0	nan	nan
Pseudomonas sp FLM 11	0	0	0	nan	nan
Pseudomonas toyotomiensis	0	0	0	nan	nan
Pullulanibacillus sp KACC 23026	0	0	0	nan	nan
Rhizopus delemar	0	0	0	nan	nan
Rhodovulum sulfidophilum	0	0	0	nan	nan
Rossellomorea aquimaris	0	0	0	nan	nan
Rothia nasimurium	0	0	0	nan	nan
Skunavirus p272	0	0	0	nan	nan
Sphingobacterium sp E70	0	0	0	nan	nan
Spiroplasma sp BIUS-1	0	0	0	nan	nan
Streptococcus chenjunshii	0	0	0	nan	nan
Streptococcus ratti	0	0	0	nan	nan
Suicoccus acidiformans	0	0	0	nan	nan
Treponema succinifaciens	0	0	0	nan	nan
Vagococcus lutrae	0	0	0	nan	nan
Vagococcus xieshaowenii	0	0	0	nan	nan
Vequintavirus JES2013	0	0	0	nan	nan
Acidipropionibacterium thoenii	0	0	0	nan	nan

Species	Median g1 (resp.)	Median g2 (non-resp.)	log2FC	p	padj
Alkalibacter rhizosphaerae	0	0	0	nan	nan
Anaerocolumna chitinilytica	0	0	0	nan	nan
Aquisphaera giovannonii	0	0	0	nan	nan
Aurodevirus intestinalis	0	0	0	nan	nan
Bradyrhizobium vignae	0	0	0	nan	nan
Buchavirus intestinalis	0	0	0	nan	nan
Campylobacter insulaenigrae	0	0	0	nan	nan
Candidatus Akkermansia timonensis	0	0	0	nan	nan
Chitinophaga caeni	0	0	0	nan	nan
Corynebacterium hindlerae	0	0	0	nan	nan
Cupriavidus malaysiensis	0	0	0	nan	nan
Curtobacterium flaccumfaciens	0	0	0	nan	nan
Diutina catenulata	0	0	0	nan	nan
Erwinia tracheiphila	0	0	0	nan	nan
Idiomarina sp OT37-5b	0	0	0	nan	nan
Listeria aquatica	0	0	0	nan	nan
Maridesulfovibrio sp	0	0	0	nan	nan
Nissabacter sp SGAir0207	0	0	0	nan	nan
Nostoc sp 'Peltigera membranacea cyanobiont' N6	0	0	0	nan	nan
Ornithinimicrobium sp HY1793	0	0	0	nan	nan
Pantoea ananatis	0	0	0	nan	nan
Paraburkholderia aromaticivorans	0	0	0	nan	nan
Polaribacter reichenbachii	0	0	0	nan	nan
Pseudomonas chlororaphis	0	0	0	nan	nan
Ralstonia mannitolilytica	0	0	0	nan	nan
Rhodococcus sp Z13	0	0	0	nan	nan
Riemerella columbina	0	0	0	nan	nan
Rugamonas sp DEMB1	0	0	0	nan	nan
Sinorhizobium chiapasense	0	0	0	nan	nan
Spongiibacter sp IMCC21906	0	0	0	nan	nan
Staphylococcus chromogenes	0	0	0	nan	nan
Sulfitobacter donghicola	0	0	0	nan	nan
Sutterella parvirubra	0	0	0	nan	nan
Thalassotalea sediminis	0	0	0	nan	nan

Species	Median g1 (resp.)	Median g2 (non-resp.)	log2FC	p	padj
Thermoanaerobacter kivui	0	0	0	nan	nan
anthomonas campestris	0	0	0	nan	nan
uncultured Desulfosarcina sp	0	0	0	nan	nan
Acididesulfobacillus acetoxydans	0	0	0	nan	nan
Allokutzneria albata	0	0	0	nan	nan
Anoxybacillus gonensis	0	0	0	nan	nan
Aspergillus turcosus	0	0	0	nan	nan
Bradyrhizobium sp C-145	0	0	0	nan	nan
Chenggangzhangella methanolivorans	0	0	0	nan	nan
Chromobacterium sp IIBBL 290-4	0	0	0	nan	nan
Citrobacter sp Y3	0	0	0	nan	nan
Companilactobacillus futsaii	0	0	0	nan	nan
Enterovibrio calviensis	0	0	0	nan	nan
Flagellimonas sp HMM57	0	0	0	nan	nan
Gordonia sp KTR9	0	0	0	nan	nan
Gordonia sp SMJS1	0	0	0	nan	nan
Hyphomonas jannaschiana	0	0	0	nan	nan
Kocuria atrinae	0	0	0	nan	nan
Lactobacillus taiwanensis	0	0	0	nan	nan
Lactococcus sp bn62	0	0	0	nan	nan
Lentilactobacillus farraginis	0	0	0	nan	nan
Lentilactobacillus kefiri	0	0	0	nan	nan
Lentilactobacillus otakiensis	0	0	0	nan	nan
Leptotrichia hongkongensis	0	0	0	nan	nan
Leucobacter viscericola	0	0	0	nan	nan
Limosilactobacillus pontis	0	0	0	nan	nan
Methyloversatilis discipulorum	0	0	0	nan	nan
Microbacterium plantarum	0	0	0	nan	nan
Microbacterium sp BH-3-3-3	0	0	0	nan	nan
Mucor racemosus	0	0	0	nan	nan
Mucor velutinosus	0	0	0	nan	nan
Paenibacillus sp 19GGS1-52	0	0	0	nan	nan
Paenibacillus sp R14(2021)	0	0	0	nan	nan
Paenibacillus stellifer	0	0	0	nan	nan

Species	Median g1 (resp.)	Median g2 (non-resp.)	log2FC	p	padj
Paenibacillus urinalis	0	0	0	nan	nan
Paramagnetospirillum magneticum	0	0	0	nan	nan
Pediococcus inopinatus	0	0	0	nan	nan
Raoultella sp DY2415	0	0	0	nan	nan
Saccharibacillus sacchari	0	0	0	nan	nan
Schinkia azotoformans	0	0	0	nan	nan
Sinorhizobium mexicanum	0	0	0	nan	nan
Skunavirus sv42	0	0	0	nan	nan
Streptomyces avicenniae	0	0	0	nan	nan
Streptomyces sp NBC 00459	0	0	0	nan	nan
Streptomyces tendae	0	0	0	nan	nan
Vequintavirus murica	0	0	0	nan	nan
Verrucomicrobium spinosum	0	0	0	nan	nan
enorhabdus bovienii	0	0	0	nan	nan
Yersinia pseudotuberculosis	0	0	0	nan	nan
Yimella sp cx-51	0	0	0	nan	nan
Alteriqipengyuania flavescens	0	0	0	nan	nan
Amycolatopsis sp YIM 10	0	0	0	nan	nan
Aspergillus niger	0	0	0	nan	nan
Aurodevirus hominis	0	0	0	nan	nan
Bacillus badius	0	0	0	nan	nan
Brasilonema sennae	0	0	0	nan	nan
Campylobacter sp RM16187	0	0	0	nan	nan
Clostridium acetobutylicum	0	0	0	nan	nan
Corynebacterium pelargi	0	0	0	nan	nan
Desulfocurvus vexinensis	0	0	0	nan	nan
Desulfovibrio aminophilus	0	0	0	nan	nan
Endozoicomonas elysicola	0	0	0	nan	nan
Geosporobacter ferrireducens	0	0	0	nan	nan
Gimesia alba	0	0	0	nan	nan
Halobacteroides halobius	0	0	0	nan	nan
Halomonas huangheensis	0	0	0	nan	nan
Halopseudomonas phragmitis	0	0	0	nan	nan
Halorubrum sp 2020YC2	0	0	0	nan	nan

Species	Median g1 (resp.)	Median g2 (non-resp.)	log2FC	p	padj
Heyndrickxia vini	0	0	0	nan	nan
Hydrogenophaga sp NH-16	0	0	0	nan	nan
Leptodesmis sichuanensis	0	0	0	nan	nan
Lichtheimia ramosa	0	0	0	nan	nan
Marinomonas sp GJ51-6	0	0	0	nan	nan
Mycobacterium sp JS623	0	0	0	nan	nan
Neisseria sp KEM232	0	0	0	nan	nan
Ornithinimicrobium flavum	0	0	0	nan	nan
Paenibacillus aurantius	0	0	0	nan	nan
Paenibacillus sp JDR-2	0	0	0	nan	nan
Polaribacter undariae	0	0	0	nan	nan
Polaromonas vacuolata	0	0	0	nan	nan
Pseudoalteromonas ruthenica	0	0	0	nan	nan
Rhizobium sp SSA 523	0	0	0	nan	nan
Ruegeria sp YS9	0	0	0	nan	nan
Spirosoma radiotolerans	0	0	0	nan	nan
Sporolactobacillus vineae	0	0	0	nan	nan
Streptomyces sp VNUA24	0	0	0	nan	nan
Streptomyces varsoviensis	0	0	0	nan	nan
Thioflavicoccus mobilis	0	0	0	nan	nan
Thiomicrorhabdus sp	0	0	0	nan	nan
Vallitalea guaymasensis	0	0	0	nan	nan
Vibrio cyclitrophicus	0	0	0	nan	nan
Arthrobacter sp DNA4	0	0	0	nan	nan
Bacteroides phage B124-14	0	0	0	nan	nan
Bacteroides phage B40-8	0	0	0	nan	nan
Bifidobacterium saguini	0	0	0	nan	nan
Cepunavirus Cp1	0	0	0	nan	nan
Clostridium carboxidivorans	0	0	0	nan	nan
Clostridium sporogenes	0	0	0	nan	nan
Companilactobacillus crustorum	0	0	0	nan	nan
Corynebacterium hadale	0	0	0	nan	nan
Corynebacterium stationis	0	0	0	nan	nan
Cryptococcus deuterogattii	0	0	0	nan	nan

Species	Median g1 (resp.)	Median g2 (non-resp.)	log2FC	p	padj
Desulfosarcina sp BuS5	0	0	0	nan	nan
Enterococcus phage IME-EFm1	0	0	0	nan	nan
Fibrisoma limi	0	0	0	nan	nan
Gallaecimonas kandeliae	0	0	0	nan	nan
Geobacillus sp E263	0	0	0	nan	nan
Lactobacillus sp 3B(2020)	0	0	0	nan	nan
Lebriduvirus gastrointestinalis	0	0	0	nan	nan
Leuconostoc fallax	0	0	0	nan	nan
Liquorilactobacillus vini	0	0	0	nan	nan
Microbacterium galbinum	0	0	0	nan	nan
Mooreparkvirus Lb3381	0	0	0	nan	nan
Mycoplasma sp Pen4	0	0	0	nan	nan
Paenibacillus sp PK1-4R	0	0	0	nan	nan
Paenibacillus thiaminolyticus	0	0	0	nan	nan
Paludibacter propionicigenes	0	0	0	nan	nan
Pseudodesulfovibrio indicus	0	0	0	nan	nan
Trichosporon ovoides	0	0	0	nan	nan
uncultured Desulfuromonas sp	0	0	0	nan	nan
Aeromicrobium massiliense	0	0	0	nan	nan
Bacillus velezensis	0	0	0	nan	nan
Bifidobacterium asteroides	0	0	0	nan	nan
Burkholderia thailandensis	0	0	0	nan	nan
Caldimonas manganoxidans	0	0	0	nan	nan
Chryseobacterium daecheongense	0	0	0	nan	nan
Citrobacter sp RHB35-C17	0	0	0	nan	nan
Corynebacterium callunae	0	0	0	nan	nan
Desulfovirgula thermocuniculi	0	0	0	nan	nan
Ensifer adhaerens	0	0	0	nan	nan
Gardnerella pickettii	0	0	0	nan	nan
Ligilactobacillus equi	0	0	0	nan	nan
Marinococcus halotolerans	0	0	0	nan	nan
Methylocystis rosea	0	0	0	nan	nan
Morococcus cerebrosus	0	0	0	nan	nan
Mycolicibacter hiberniae	0	0	0	nan	nan

Species	Median g1 (resp.)	Median g2 (non-resp.)	log2FC	p	padj
Paraburkholderia mimosarum	0	0	0	nan	nan
Pleomorphomonas oryzae	0	0	0	nan	nan
Pseudomonas lundensis	0	0	0	nan	nan
Psychrobacillus sp AK 1817	0	0	0	nan	nan
Siccibacter colletis	0	0	0	nan	nan
Streptococcus phage P0091	0	0	0	nan	nan
Streptomyces calvus	0	0	0	nan	nan
Streptomyces sp NBC 01334	0	0	0	nan	nan
Thermaerobacter marianensis	0	0	0	nan	nan
Tritonibacter scottomollicae	0	0	0	nan	nan
Urbifossiella limnaea	0	0	0	nan	nan
Vibrio natriegens	0	0	0	nan	nan
Agrococcus sp Marseille-Q4369	0	0	0	nan	nan
Anaeromyxobacter dehalogenans	0	0	0	nan	nan
Bifidobacterium gallicum	0	0	0	nan	nan
Borrelia miyamotoi	0	0	0	nan	nan
Campylobacter sp RM6137	0	0	0	nan	nan
Cereibacter sphaeroides	0	0	0	nan	nan
Chitiniphilus shinanonensis	0	0	0	nan	nan
Clostridium sp 16K-1-R1	0	0	0	nan	nan
Companilactobacillus farciminis	0	0	0	nan	nan
Companilactobacillus ginsenosidimutans	0	0	0	nan	nan
Corynebacterium rouxii	0	0	0	nan	nan
Crossiella sp CA-258035	0	0	0	nan	nan
Cupriavidus pauculus	0	0	0	nan	nan
Curvularia hawaiiensis	0	0	0	nan	nan
Desulfomicrobium baculatum	0	0	0	nan	nan
Desulfonema magnum	0	0	0	nan	nan
Desulforamulus hydrothermalis	0	0	0	nan	nan
Desulfurispirillum indicum	0	0	0	nan	nan
Enterococcus sp DA9	0	0	0	nan	nan
Flaviramulus sp BrNp1-15	0	0	0	nan	nan
Flavobacterium agricola	0	0	0	nan	nan
Flavobacterium sp N502536	0	0	0	nan	nan

Species	Median g1 (resp.)	Median g2 (non-resp.)	log2FC	p	padj
Hoyosella subflava	0	0	0	nan	nan
Lactococcus petauri	0	0	0	nan	nan
Lactococcus sp LG592	0	0	0	nan	nan
Leadbettera azotonutricia	0	0	0	nan	nan
Leclercia sp 119287	0	0	0	nan	nan
Ligilactobacillus pobuzihii	0	0	0	nan	nan
Lysobacter capsici	0	0	0	nan	nan
Lysobacter daejeonensis	0	0	0	nan	nan
Marivirga sp BDSF4-3	0	0	0	nan	nan
Metaclostridioides mangenotii	0	0	0	nan	nan
Methanobrevibacter olleyae	0	0	0	nan	nan
Methyloferula stellata	0	0	0	nan	nan
Microbacterium chocolatum	0	0	0	nan	nan
Micromonospora siamensis	0	0	0	nan	nan
Mycobacterium riyadhense	0	0	0	nan	nan
Mycolicibacterium confluentis	0	0	0	nan	nan
Neobacillus sp YX16	0	0	0	nan	nan
Nocardia farcinica	0	0	0	nan	nan
Nocardioides humi	0	0	0	nan	nan
Nocardioides marinisabuli	0	0	0	nan	nan
Nocardioides sp JS614	0	0	0	nan	nan
Oleidesulfovibrio alaskensis	0	0	0	nan	nan
Onchocerca volvulus	0	0	0	nan	nan
Paenibacillus alvei	0	0	0	nan	nan
Paenibacillus macerans	0	0	0	nan	nan
Paenibacillus massiliensis	0	0	0	nan	nan
Paenibacillus polymyxa	0	0	0	nan	nan
Paradevosia shaoguanensis	0	0	0	nan	nan
Planctomycetaceae bacterium SH412	0	0	0	nan	nan
Pseudoalteromonas shioyasakiensis	0	0	0	nan	nan
Reyranella massiliensis	0	0	0	nan	nan
Rhizomucor pusillus	0	0	0	nan	nan
Rhodomicrobium lacus	0	0	0	nan	nan
Rickettsiella endosymbiont of Dermanyssus gallinae	0	0	0	nan	nan

Species	Median g1 (resp.)	Median g2 (non-resp.)	log2FC	p	padj
Salisaeta longa	0	0	0	nan	nan
Scolecobasidium constrictum	0	0	0	nan	nan
Shewanella putrefaciens	0	0	0	nan	nan
Spiroplasma diminutum	0	0	0	nan	nan
Staphylococcus succinus	0	0	0	nan	nan
Streptococcus phage P7133	0	0	0	nan	nan
Streptomyces antimycoticus	0	0	0	nan	nan
Streptomyces kanamyceticus	0	0	0	nan	nan
Streptomyces sp NBC 00223	0	0	0	nan	nan
Streptomyces sp NBC 01341	0	0	0	nan	nan
Sulfitobacter faviae	0	0	0	nan	nan
Thermoanaerobacterium sp RBIITD	0	0	0	nan	nan
Vagococcus fluvialis	0	0	0	nan	nan
anthomonas oryzae	0	0	0	nan	nan
enoacremonium recifei	0	0	0	nan	nan
uncultured Draconibacterium sp	0	0	0	nan	nan
uncultured Methylophaga sp	0	0	0	nan	nan
Acidisarcina polymorpha	0	0	0	nan	nan
Actinobacillus ureae	0	0	0	nan	nan
Actinoplanes sp NBC 00393	0	0	0	nan	nan
Arthrobacter sp Y-9	0	0	0	nan	nan
Aspergillus alliaceus	0	0	0	nan	nan
Bacillus sp N1-1	0	0	0	nan	nan
Bifidobacterium tsurumiense	0	0	0	nan	nan
Bordetella holmesii	0	0	0	nan	nan
Bradyrhizobium murdochi	0	0	0	nan	nan
Caulobacter segnis	0	0	0	nan	nan
Chryseobacterium sp 6424	0	0	0	nan	nan
Clostridium saccharobutylicum	0	0	0	nan	nan
Coprinopsis cinerea	0	0	0	nan	nan
Corynebacterium diphtheriae	0	0	0	nan	nan
Crassaminicella thermophila	0	0	0	nan	nan
Cylindrospermum stagnale	0	0	0	nan	nan
Ereboglobus luteus	0	0	0	nan	nan

Species	Median g1 (resp.)	Median g2 (non-resp.)	log2FC	p	padj
Erwinia tasmaniensis	0	0	0	nan	nan
Exophiala xenobiotica	0	0	0	nan	nan
Faecalibacter sp LW9	0	0	0	nan	nan
Gardnerella vaginalis	0	0	0	nan	nan
Hydrogenophaga sp BPS33	0	0	0	nan	nan
Leptotrichia shahii	0	0	0	nan	nan
Lunatimonas lonarensis	0	0	0	nan	nan
Malassezia globosa	0	0	0	nan	nan
Mycolicibacterium smegmatis	0	0	0	nan	nan
Nannizziopsis arthrosporioides	0	0	0	nan	nan
Neisseria dumasiana	0	0	0	nan	nan
Nocardioides scoriae	0	0	0	nan	nan
Novosphingobium resinovorum	0	0	0	nan	nan
Novosphingobium sp 9	0	0	0	nan	nan
Paenibacillus peoriae	0	0	0	nan	nan
Paenibacillus pini	0	0	0	nan	nan
Piorkowskivirus SW22	0	0	0	nan	nan
Rhizobium sp NZLR1	0	0	0	nan	nan
Sphingobium cloacae	0	0	0	nan	nan
Staphylococcus lugdunensis	0	0	0	nan	nan
Streptococcus sp DAT741	0	0	0	nan	nan
Streptomyces aurantiacus	0	0	0	nan	nan
Streptomyces canus	0	0	0	nan	nan
Streptomyces sp KPB2	0	0	0	nan	nan
Streptomyces spinosirectus	0	0	0	nan	nan
Thalassotalea hakodatensis	0	0	0	nan	nan
Trichomonas vaginalis	0	0	0	nan	nan
Trinickia caryophylli	0	0	0	nan	nan
Verrucomicrobium sp GAS474	0	0	0	nan	nan
Arawnvirus arawn	0	0	0	nan	nan
Bartonella choladocola	0	0	0	nan	nan
Brevibacterium linens	0	0	0	nan	nan
Brevibacterium sp BRM-1	0	0	0	nan	nan
Candidatus Rickettsiella viridis	0	0	0	nan	nan

Species	Median g1 (resp.)	Median g2 (non-resp.)	log2FC	p	padj
Clostridium sp 'deep sea'	0	0	0	nan	nan
Desulfurobacterium thermolithotrophum	0	0	0	nan	nan
Fusarium solani	0	0	0	nan	nan
Geobacter pickeringii	0	0	0	nan	nan
Gimesia fumaroli	0	0	0	nan	nan
Halobacterium litoreum	0	0	0	nan	nan
Kitasatospora setae	0	0	0	nan	nan
Komagataeibacter europaeus	0	0	0	nan	nan
Lentzea sp DG1S-22	0	0	0	nan	nan
Lichtheimia corymbifera	0	0	0	nan	nan
Lysinibacillus sp 2017	0	0	0	nan	nan
Mucilaginibacter sp KACC 22773	0	0	0	nan	nan
Nocardioides sp cx-173	0	0	0	nan	nan
Paenibacillus sp PSB04	0	0	0	nan	nan
Paraburkholderia phytofirmans	0	0	0	nan	nan
Phialophora tarda	0	0	0	nan	nan
Pseudofrankia inefficax	0	0	0	nan	nan
Pseudomonas brenneri	0	0	0	nan	nan
Pseudomonas resinovorans	0	0	0	nan	nan
Pseudonocardia abyssalis	0	0	0	nan	nan
Rhizobium sp 11515TR	0	0	0	nan	nan
Sphingomonas piscis	0	0	0	nan	nan
Streptomyces sp So13 3	0	0	0	nan	nan
Sulfurimonas sp HSL-3221	0	0	0	nan	nan
Terricaulis silvestris	0	0	0	nan	nan
Trypanosoma cruzi	0	0	0	nan	nan
Vanrija sp TS 01	0	0	0	nan	nan
uncultured Desulfovibrio sp	0	0	0	nan	nan
uncultured Pleomorphomonas sp	0	0	0	nan	nan
unidentified bacterial endosymbiont	0	0	0	nan	nan
Aeromonas veronii	0	0	0	nan	nan
Aminobacterium mobile	0	0	0	nan	nan
Bacillus shivajii	0	0	0	nan	nan
Bifidobacterium actinocoloniiforme	0	0	0	nan	nan

Species	Median g1 (resp.)	Median g2 (non-resp.)	log2FC	p	padj
Cedecea sp FDAARGOS 727	0	0	0	nan	nan
Chryseobacterium taklimakanense	0	0	0	nan	nan
Clostridium akagii	0	0	0	nan	nan
Dehalogenimonas formicexedens	0	0	0	nan	nan
Dermacoccus nishinomiyaensis	0	0	0	nan	nan
Desulfofarcimen acetoxidans	0	0	0	nan	nan
Desulfosarcina alkanivorans	0	0	0	nan	nan
Desulfotomaculum nigrificans	0	0	0	nan	nan
Echinococcus granulosus	0	0	0	nan	nan
Efquatrovirus EfaCPT1	0	0	0	nan	nan
Efquatrovirus IME196	0	0	0	nan	nan
Efquatrovirus LY0322	0	0	0	nan	nan
Efquatrovirus SHEF5	0	0	0	nan	nan
Enterobacter cloacae complex sp ECL405	0	0	0	nan	nan
Enterobacter sp JBIWA005	0	0	0	nan	nan
Flavobacterium sp N1846	0	0	0	nan	nan
Gardnerella greenwoodii	0	0	0	nan	nan
Haliea salexigens	0	0	0	nan	nan
Justusliebigvirus PD06	0	0	0	nan	nan
Justusliebigvirus PHB05	0	0	0	nan	nan
Justusliebigvirus alia	0	0	0	nan	nan
Justusliebigvirus muut	0	0	0	nan	nan
Karamvirus pg7	0	0	0	nan	nan
Lelliottia amnigena	0	0	0	nan	nan
Levilactobacillus parabrevis	0	0	0	nan	nan
Mesorhizobium loti	0	0	0	nan	nan
Microbulbifer sp MI-G	0	0	0	nan	nan
Microlunatus soli	0	0	0	nan	nan
Mycolicibacterium sp HK-90	0	0	0	nan	nan
Mycoplasmopsis pullorum	0	0	0	nan	nan
Neisseria gonorrhoeae	0	0	0	nan	nan
Oceanobacillus kimchii	0	0	0	nan	nan
Pectobacterium parmentieri	0	0	0	nan	nan
Periweissella cryptocerci	0	0	0	nan	nan

Species	Median g1 (resp.)	Median g2 (non-resp.)	log2FC	p	padj
Phapecoctavirus nepoznato	0	0	0	nan	nan
Polaromonas sp JS666	0	0	0	nan	nan
Porphyromonas sp oral taxon 275	0	0	0	nan	nan
Proteus columbae	0	0	0	nan	nan
Pseudomonas fragi	0	0	0	nan	nan
Ruegeria sp SCSIO 43209	0	0	0	nan	nan
Saccharobesus litoralis	0	0	0	nan	nan
Salisediminibacterium beveridgei	0	0	0	nan	nan
Schizophyllum commune	0	0	0	nan	nan
Sinomonas sp R1AF57	0	0	0	nan	nan
Slopekvirus kp27	0	0	0	nan	nan
Streptococcus phage SW12	0	0	0	nan	nan
Streptomyces flavidovirens	0	0	0	nan	nan
Terrabacter sp C0L 2	0	0	0	nan	nan
Thermocoleostomius sinensis	0	0	0	nan	nan
Aceticella autotrophica	0	0	0	nan	nan
Acetivibrio clariflavus	0	0	0	nan	nan
Acetoanaerobium sticklandii	0	0	0	nan	nan
Achromobacter spanius	0	0	0	nan	nan
Actinacidiphila glaucinigra	0	0	0	nan	nan
Actinoallomurus sp NBC 01490	0	0	0	nan	nan
Actinomadura graeca	0	0	0	nan	nan
Actinomadura madurae	0	0	0	nan	nan
Actinomadura verrucosospora	0	0	0	nan	nan
Actinomortierella wolfii	0	0	0	nan	nan
Actinomyces qiguomingii	0	0	0	nan	nan
Actinoplanes derwentensis	0	0	0	nan	nan
Actinoplanes sp L3-i22	0	0	0	nan	nan
Actinoplanes sp N902-109	0	0	0	nan	nan
Actinoplanes subtropicus	0	0	0	nan	nan
Agrobacterium tumefaciens	0	0	0	nan	nan
Agrobacterium vitis	0	0	0	nan	nan
Alicyclobacillus cycloheptanicus	0	0	0	nan	nan
Alicyclobacillus fastidiosus	0	0	0	nan	nan

Species	Median g1 (resp.)	Median g2 (non-resp.)	log2FC	p	padj
Alicyclobacillus mengziensis	0	0	0	nan	nan
Alienimonas californiensis	0	0	0	nan	nan
Alkaliphilus sp B6464	0	0	0	nan	nan
Allobaculum mucilyticum	0	0	0	nan	nan
Allobaculum sp Allo2	0	0	0	nan	nan
Aminithiophilus ramosus	0	0	0	nan	nan
Amycolatopsis acidiphila	0	0	0	nan	nan
Amycolatopsis albispora	0	0	0	nan	nan
Amycolatopsis sp 2-2	0	0	0	nan	nan
Amycolatopsis sp NBC 01480	0	0	0	nan	nan
Anaeromyxobacter sp Fw109-5	0	0	0	nan	nan
Aneurinibacillus sp Ricciae BoGa-3	0	0	0	nan	nan
Aquihabitans sp G128	0	0	0	nan	nan
Aquipluma nitroreducens	0	0	0	nan	nan
Arthrobacter agilis	0	0	0	nan	nan
Arthrobacter crystallopoietes	0	0	0	nan	nan
Aspergillus calidoustus	0	0	0	nan	nan
Aspergillus fumigatiaffinis	0	0	0	nan	nan
Azospirillum brasilense	0	0	0	nan	nan
Azospirillum thermophilum	0	0	0	nan	nan
Bacillus glycinifermentans	0	0	0	nan	nan
Bacillus mycoides	0	0	0	nan	nan
Bacillus sp FJAT-18017	0	0	0	nan	nan
Bacillus sp FJAT-42376	0	0	0	nan	nan
Bacillus subtilis	0	0	0	nan	nan
Baekduia alba	0	0	0	nan	nan
Bifidobacterium lemurum	0	0	0	nan	nan
Bifidobacterium magnum	0	0	0	nan	nan
Bifidobacterium sp ESL0790	0	0	0	nan	nan
Biomaibacter acetigenes	0	0	0	nan	nan
Blastomyces emzantsi	0	0	0	nan	nan
Blattabacterium cuenoti	0	0	0	nan	nan
Blautia sp KLE 1732 HM 1032	0	0	0	nan	nan
Brachybacterium phenoliresistens	0	0	0	nan	nan

Species	Median g1 (resp.)	Median g2 (non-resp.)	log2FC	p	padj
Bradyrhizobium diazoefficiens	0	0	0	nan	nan
Bradyrhizobium japonicum	0	0	0	nan	nan
Bradyrhizobium prioritasuperba	0	0	0	nan	nan
Breoghania sp	0	0	0	nan	nan
Brevibacillus borstelensis	0	0	0	nan	nan
Brevibacillus composti	0	0	0	nan	nan
Brevibacillus marinus	0	0	0	nan	nan
Brevibacillus massiliensis	0	0	0	nan	nan
Brevibacillus ruminantium	0	0	0	nan	nan
Breznakiella homolactica	0	0	0	nan	nan
Brucepastera parasyntrophica	0	0	0	nan	nan
Burkholderia gladioli	0	0	0	nan	nan
Caldibacillus thermoamylovorans	0	0	0	nan	nan
Caldisalinibacter kiritimatiensis	0	0	0	nan	nan
Caloramator sp Dgby cultured 2	0	0	0	nan	nan
Caloramator sp E03	0	0	0	nan	nan
Caloramator sp mosi 1	0	0	0	nan	nan
Caloranaerobacter azorensis	0	0	0	nan	nan
Candida sp PB3310	0	0	0	nan	nan
Candidatus Desulforudis audaxviator	0	0	0	nan	nan
Candidatus Desulfovibrio trichonymphae	0	0	0	nan	nan
Candidatus Promineifilum breve	0	0	0	nan	nan
Candidatus Thiodictyon syntrophicum	0	0	0	nan	nan
Caproiciproducens sp NJN-50	0	0	0	nan	nan
Capsulimonas corticalis	0	0	0	nan	nan
Caulobacter flavus	0	0	0	nan	nan
Cellulosimicrobium cellulans	0	0	0	nan	nan
Chaetomium globosum	0	0	0	nan	nan
Chitinimonas koreensis	0	0	0	nan	nan
Chitinophaga sancti	0	0	0	nan	nan
Chryseobacterium indologenes	0	0	0	nan	nan
Clostridium aceticum	0	0	0	nan	nan
Clostridium algidicarnis	0	0	0	nan	nan
Clostridium cochlearium	0	0	0	nan	nan

Species	Median g1 (resp.)	Median g2 (non-resp.)	log2FC	p	padj
Clostridium drakei	0	0	0	nan	nan
Clostridium fermenticellae	0	0	0	nan	nan
Clostridium gasigenes	0	0	0	nan	nan
Clostridium manihotivorum	0	0	0	nan	nan
Clostridium scatologenes	0	0	0	nan	nan
Clostridium sp 001	0	0	0	nan	nan
Clostridium sp BNL1100	0	0	0	nan	nan
Clostridium sp MB40-C1	0	0	0	nan	nan
Clostridium sp OS1-26	0	0	0	nan	nan
Clostridium sulfidigenes	0	0	0	nan	nan
Clostridium tagluense	0	0	0	nan	nan
Clostridium thermarum	0	0	0	nan	nan
Cohnella cholangitidis	0	0	0	nan	nan
Cohnella sp LGH	0	0	0	nan	nan
Conexibacter sp SYSU D00693	0	0	0	nan	nan
Cupriavidus metallidurans	0	0	0	nan	nan
Cupriavidus necator	0	0	0	nan	nan
Cupriavidus taiwanensis	0	0	0	nan	nan
Cystobacter fuscus	0	0	0	nan	nan
Cytobacillus firmus	0	0	0	nan	nan
Dactylosporangium aurantiacum	0	0	0	nan	nan
Dactylosporangium sp AC04546	0	0	0	nan	nan
Dactylosporangium sp NBC 01737	0	0	0	nan	nan
Deinococcus ficus	0	0	0	nan	nan
Desulfobulbus oralis	0	0	0	nan	nan
Desulfoluna limicola	0	0	0	nan	nan
Desulfolutivibrio sulfoxidireducens	0	0	0	nan	nan
Desulfuromonas soudanensis	0	0	0	nan	nan
Desulfuromonas versatilis	0	0	0	nan	nan
Domibacillus sp DTU 2020 1001157 1 SI ALB TIR 016	0	0	0	nan	nan
Draconibacterium orientale	0	0	0	nan	nan
Echinococcus multilocularis	0	0	0	nan	nan
Erwinia rhapontici	0	0	0	nan	nan
Ferribacterium limneticum	0	0	0	nan	nan

Species	Median g1 (resp.)	Median g2 (non-resp.)	log2FC	p	padj
Ferrovibrio terrae	0	0	0	nan	nan
Friedmanniella luteola	0	0	0	nan	nan
Geobacter sp FeAm09	0	0	0	nan	nan
Gudongella oleilytica	0	0	0	nan	nan
Haliovirga abyssi	0	0	0	nan	nan
Halopseudomonas litoralis	0	0	0	nan	nan
Hathewaya histolytica	0	0	0	nan	nan
Humisphaera borealis	0	0	0	nan	nan
Hyalangium minutum	0	0	0	nan	nan
Hymenobacter busanensis	0	0	0	nan	nan
Hymenobacter nivis	0	0	0	nan	nan
Hymenobacter sp 5317J-9	0	0	0	nan	nan
Hymenobacter sp GOD-10R	0	0	0	nan	nan
Hymenobacter sp PAMC 26628	0	0	0	nan	nan
Hymenobacter tibetensis	0	0	0	nan	nan
Ignavibacterium sp	0	0	0	nan	nan
Ilyonectria destructans	0	0	0	nan	nan
Indioceanicola profundi	0	0	0	nan	nan
Jiangella alkaliphila	0	0	0	nan	nan
Kallotenue papyrolyticum	0	0	0	nan	nan
Kibdelosporangium phytohabitans	0	0	0	nan	nan
Kiritimatiella glycovorans	0	0	0	nan	nan
Kocuria rosea	0	0	0	nan	nan
Koleobacter methoxysyntrophicus	0	0	0	nan	nan
Kroppenstedtia eburnea	0	0	0	nan	nan
Kutzneria sp CA-103260	0	0	0	nan	nan
Labilithrix luteola	0	0	0	nan	nan
Lasiodiplodia pseudotheobromae	0	0	0	nan	nan
Limnochorda pilosa	0	0	0	nan	nan
Limnochorda sp L945t	0	0	0	nan	nan
Luteitalea pratensis	0	0	0	nan	nan
Luteitalea sp TBR-22	0	0	0	nan	nan
Luteolibacter ambystomatis	0	0	0	nan	nan
Lysobacter antibioticus	0	0	0	nan	nan

Species	Median g1 (resp.)	Median g2 (non-resp.)	log2FC	p	padj
Lysobacter enzymogenes	0	0	0	nan	nan
Mangrovibacterium marinum	0	0	0	nan	nan
Marinifilum fragile	0	0	0	nan	nan
Marispirochaeta aestuarii	0	0	0	nan	nan
Massilia violaceinigra	0	0	0	nan	nan
Micromonospora sp NBC 01796	0	0	0	nan	nan
Miniphocaeibacter halophilus	0	0	0	nan	nan
Moorella glycerini	0	0	0	nan	nan
Moorella sp Hama-1	0	0	0	nan	nan
Moorella thermoacetica	0	0	0	nan	nan
Mucor circinelloides	0	0	0	nan	nan
Nannocystis poenicansa	0	0	0	nan	nan
Neobacillus sp Marseille-Q6967	0	0	0	nan	nan
Neobacillus sp OS1-33	0	0	0	nan	nan
Niallia taxi	0	0	0	nan	nan
Nitrospina watsonii	0	0	0	nan	nan
Nocardia asteroides	0	0	0	nan	nan
Nocardia brasiliensis	0	0	0	nan	nan
Nonomuraea candida	0	0	0	nan	nan
Nonomuraea fuscirosea	0	0	0	nan	nan
Nonomuraea gerenzanensis	0	0	0	nan	nan
Nonomuraea glycinis	0	0	0	nan	nan
Nonomuraea sp NBC 00507	0	0	0	nan	nan
Nonomuraea sp NBC 01738	0	0	0	nan	nan
Oceanidesulfovibrio marinus	0	0	0	nan	nan
Opitutus sp WL0086	0	0	0	nan	nan
Orrella dioscoreae	0	0	0	nan	nan
Paenibacillus albicereus	0	0	0	nan	nan
Paenibacillus antibioticophila	0	0	0	nan	nan
Paenibacillus barcinonensis	0	0	0	nan	nan
Paenibacillus beijingensis	0	0	0	nan	nan
Paenibacillus borealis	0	0	0	nan	nan
Paenibacillus cellulosilyticus	0	0	0	nan	nan
Paenibacillus chitinolyticus	0	0	0	nan	nan

Species	Median g1 (resp.)	Median g2 (non-resp.)	log2FC	p	padj
Paenibacillus darwinianus	0	0	0	nan	nan
Paenibacillus elgii	0	0	0	nan	nan
Paenibacillus gorillae	0	0	0	nan	nan
Paenibacillus graminis	0	0	0	nan	nan
Paenibacillus ihbetae	0	0	0	nan	nan
Paenibacillus konkukensis	0	0	0	nan	nan
Paenibacillus kribbensis	0	0	0	nan	nan
Paenibacillus lycopersici	0	0	0	nan	nan
Paenibacillus naphthalenovorans	0	0	0	nan	nan
Paenibacillus pabuli	0	0	0	nan	nan
Paenibacillus physcomitrellae	0	0	0	nan	nan
Paenibacillus protaetiae	0	0	0	nan	nan
Paenibacillus psychroresistens	0	0	0	nan	nan
Paenibacillus silvisoli	0	0	0	nan	nan
Paenibacillus sp 481	0	0	0	nan	nan
Paenibacillus sp D2 2	0	0	0	nan	nan
Paenibacillus sp FSL H7-0357	0	0	0	nan	nan
Paenibacillus sp FSL P4-0081	0	0	0	nan	nan
Paenibacillus sp JZ16	0	0	0	nan	nan
Paenibacillus sp MMS20-IR301	0	0	0	nan	nan
Paenibacillus sp SYP-B4298	0	0	0	nan	nan
Paenibacillus sp URB8-2	0	0	0	nan	nan
Paenibacillus sp YPD9-1	0	0	0	nan	nan
Paenibacillus sp sptzw28	0	0	0	nan	nan
Paenibacillus spongiae	0	0	0	nan	nan
Paenibacillus tianjinensis	0	0	0	nan	nan
Paenibacillus tritici	0	0	0	nan	nan
Paenibacillus woosongensis	0	0	0	nan	nan
Paenibacillus yonginensis	0	0	0	nan	nan
Paludibaculum fermentans	0	0	0	nan	nan
Paracoccidioides brasiliensis	0	0	0	nan	nan
Paracoccus sanguinis	0	0	0	nan	nan
Pelolinea submarina	0	0	0	nan	nan
Pelosinus fermentans	0	0	0	nan	nan

Species	Median g1 (resp.)	Median g2 (non-resp.)	log2FC	p	padj
Peribacillus asahii	0	0	0	nan	nan
Peribacillus frigoritolerans	0	0	0	nan	nan
Peterkaempfera bronchialis	0	0	0	nan	nan
Phytohabitans suffuscus	0	0	0	nan	nan
Pimelobacter simplex	0	0	0	nan	nan
Pirellulimonas nuda	0	0	0	nan	nan
Piscinibacter sp XHJ-5	0	0	0	nan	nan
Planctomyces sp SH-PL62	0	0	0	nan	nan
Pleurostoma richardsiae	0	0	0	nan	nan
Pontibacter russatus	0	0	0	nan	nan
Porphyromonas pogonae	0	0	0	nan	nan
Priestia megaterium	0	0	0	nan	nan
Proteiniphilum saccharofermentans	0	0	0	nan	nan
Pseudodesulfovibrio cashew	0	0	0	nan	nan
Pseudodesulfovibrio mercurii	0	0	0	nan	nan
Pseudomonas fluorescens	0	0	0	nan	nan
Pseudomonas otitidis	0	0	0	nan	nan
Pseudoxanthomonas suwonensis	0	0	0	nan	nan
Purpureocillium lilacinum	0	0	0	nan	nan
Pyxidicoccus parkwaysis	0	0	0	nan	nan
Ramlibacter tataouinensis	0	0	0	nan	nan
Rhizobium leguminosarum	0	0	0	nan	nan
Rhizopus arrhizus	0	0	0	nan	nan
Rhodocaloribacter litoris	0	0	0	nan	nan
Rhodothermus marinus	0	0	0	nan	nan
Rhodotorula toruloides	0	0	0	nan	nan
Rhodovastum atsumiense	0	0	0	nan	nan
Roseibium sp Sym1	0	0	0	nan	nan
Roseomonas sp OT10	0	0	0	nan	nan
Rossellomorea marisflavi	0	0	0	nan	nan
Ruminiclostridium cellobioparum	0	0	0	nan	nan
Ruminococcus sp FMB-CY1	0	0	0	nan	nan
Ruminococcus sp FMBCY1	0	0	0	nan	nan
Saccharibacillus brassicae	0	0	0	nan	nan

Species	Median g1 (resp.)	Median g2 (non-resp.)	log2FC	p	padj
Saccharopolyspora erythraea	0	0	0	nan	nan
Saccharopolyspora rosea	0	0	0	nan	nan
Saccharothrix syringae	0	0	0	nan	nan
Salidesulfovibrio onnuriiensis	0	0	0	nan	nan
Salinibacter ruber	0	0	0	nan	nan
Sarocladium strictum	0	0	0	nan	nan
Serpentinicella alkaliphila	0	0	0	nan	nan
Solibacillus sp R5-41	0	0	0	nan	nan
Sorangium cellulosum	0	0	0	nan	nan
Sphingobacterium spiritivorum	0	0	0	nan	nan
Sphingosinicella microcystinivorans	0	0	0	nan	nan
Sporomusa termitida	0	0	0	nan	nan
Sporosarcina ureae	0	0	0	nan	nan
Streptomyces albus	0	0	0	nan	nan
Streptomyces armeniacus	0	0	0	nan	nan
Streptomyces chartreusis	0	0	0	nan	nan
Streptomyces europaeiscabiei	0	0	0	nan	nan
Streptomyces lydicus	0	0	0	nan	nan
Streptomyces marincola	0	0	0	nan	nan
Streptomyces niveus	0	0	0	nan	nan
Streptomyces sp XD-27	0	0	0	nan	nan
Streptomyces tsukubensis	0	0	0	nan	nan
Streptomyces venezuelae	0	0	0	nan	nan
Streptomyces virginiae	0	0	0	nan	nan
Streptomyces xinghaiensis	0	0	0	nan	nan
Streptosporangium sp 'caverna'	0	0	0	nan	nan
Strongyloides stercoralis	0	0	0	nan	nan
Sulfidibacter corallicola	0	0	0	nan	nan
Sutcliffiella horikoshii	0	0	0	nan	nan
Sutterella megalosphaeroides	0	0	0	nan	nan
Syntrophotalea acetylenica	0	0	0	nan	nan
Tannockella kyphosi	0	0	0	nan	nan
Taurinivorans muris	0	0	0	nan	nan
Tautonia plasticadhaerens	0	0	0	nan	nan

Species	Median g1 (resp.)	Median g2 (non-resp.)	log2FC	p	padj
Tepidibacter aestuarii	0	0	0	nan	nan
Tepidibacter hydrothermalis	0	0	0	nan	nan
Tessaracoccus defluvii	0	0	0	nan	nan
Thalassobaculum sp OXR-137	0	0	0	nan	nan
Thermaerobacter sp PB12/4term	0	0	0	nan	nan
Thermoanaerobacterium sp CMT5567-10	0	0	0	nan	nan
Thermomonospora amylolytica	0	0	0	nan	nan
Thioalkalivibrio versutus	0	0	0	nan	nan
Thyridium curvatum	0	0	0	nan	nan
Tissierella sp MB52-C2	0	0	0	nan	nan
Trichophyton interdigitale	0	0	0	nan	nan
Tumebacillus flagellatus	0	0	0	nan	nan
Umezawaea sp Da 62-37	0	0	0	nan	nan
Vallitalea pronyensis	0	0	0	nan	nan
ylophilus rhododendri	0	0	0	nan	nan
Yarrowia lipolytica	0	0	0	nan	nan
[Candida] auris	0	0	0	nan	nan
[Phialemoniopsis] sp UDSM-2020	0	0	0	nan	nan
uncultured Acetobacterium sp	0	0	0	nan	nan
uncultured Acetobacteroides sp	0	0	0	nan	nan
uncultured Anaeromusa sp	0	0	0	nan	nan
uncultured Enterococcus sp	0	0	0	nan	nan
uncultured Hyphomonas sp	0	0	0	nan	nan
uncultured Macellibacteroides sp	0	0	0	nan	nan

Table S5. Complete differential-abundance results for all imputed metabolites (responders vs non-responders at T0), annotated by functional class. The full table (255 metabolites) is provided as a separate machine-readable file (Supplementary_Table_S5.tsv).

Metabolite	Median g1 (resp.)	Median g2 (non-resp.)	Effect	p	padj	Functional class (refined)	Functional class (granular)
ADMA	17809447.908947	6813688.4998581	10995759.409088898	0.004740357681534152	0.34768432259781745	Other	Carnitine and methylated compounds
Pantothete	543938748.574971	242585162.82393998	301353585.751031	0.009696186166774402	0.34768432259781745	Other	Vitamins and cofactors
Inosine	45172602.44624865	-19974979.70848895	65147582.1547376	0.009696186166774402	0.34768432259781745	Nucleosides / DNA-RNA	Nucleobases / nucleosides
Propionate	139077994.4182145	92723600.6822904	46354393.735924095	0.009696186166774402	0.34768432259781745	Other	SCFA and fermentation products
Guanosine	-207160.87343918148	-17743851.0054747	17536690.13203552	0.009696186166774402	0.34768432259781745	Other	Nucleobases / nucleosides
Arginine	1253714.3105384605	-2191718.355609215	3445432.6661476754	0.009696186166774402	0.34768432259781745	Other	Amino acids
Quinic acid	142179982.67625552	5687940.58833445	136492042.08792108	0.013466925231631115	0.34768432259781745	Aromatic / phenolic metabolism	Phenolic / aromatic microbial metabolites
C36 5 PC plasmalogen A	-522510.3385475865	-4316966.81562507	3794456.477077483	0.013466925231631115	0.34768432259781745	Other	Lipids and fatty acids
Leucine	2524881297.362125	1806389006.36096	718492291.0011649	0.018207282913165267	0.34768432259781745	Other	Amino acids
Asparagine	55197910.80750975	-16080751.847326951	71278662.6548367	0.018207282913165267	0.34768432259781745	Other	Amino acids
Linoleoyl ethanolamide 2	-1616958.65156893	-48783854.6836628	47166896.032093875	0.018207282913165267	0.34768432259781745	Other	Lipids and fatty acids
Asparagine	30633277.3483695	-2678391.4997465652	33311668.848116066	0.018207282913165267	0.34768432259781745	Other	Amino acids
2 hydroxymyristic acid	-2317776.4553651297	471971.4947183995	-2789747.950083529	0.018207282913165267	0.34768432259781745	Other	Lipids and fatty acids
Nicotinic acid	492537881.9284915	326915434.899794	165622447.0286975	0.024455936220642106	0.34768432259781745	Other	Vitamins and cofactors
Methionine	720812071.2262275	590920760.7714405	129891310.45478702	0.024455936220642106	0.34768432259781745	Other	Amino acids
Butyrate	600253988.928371	484656054.760127	115597934.16824394	0.024455936220642106	0.34768432259781745	SCFA / BCFA	SCFA and fermentation products
Proline betaine	296039622.167773	213914188.6591375	82125433.50863552	0.024455936220642106	0.34768432259781745	Other	Amino acids
Hydroxyisovaleric acid	53106642.85097815	23506471.7860002	29600171.06497795	0.024455936220642106	0.34768432259781745	SCFA / BCFA	SCFA and fermentation products
Linoleoyl ethanolamide	209604.97833212843	-5224142.091394295	5433747.069726423	0.024455936220642106	0.34768432259781745	Other	Lipids and fatty acids
Chenodeoxycholate	107833970.58445409	51978763.247792706	55855207.33666138	0.03199741435035553	0.34768432259781745	Other	Bile acids
Dihydroxybenzoic Acid	91496668.3809195	73259092.58986384	18237575.791055664	0.03199741435035553	0.34768432259781745	Other	Phenolic / aromatic microbial metabolites
Malonate	6006220.981093315	-12154732.24399445	18160953.225087766	0.03199741435035553	0.34768432259781745	Organic acids	Organic acids and TCA-related
Pseudouridine	9938463.377587419	-7755744.933289135	17694208.310876556	0.03199741435035553	0.34768432259781745	Other	Nucleobases / nucleosides
Xanthine	970652066.0016665	800523594.1804645	170128471.82120204	0.041478129713423836	0.34768432259781745	Other	Nucleobases / nucleosides
N acetylspermidine	35478862.33871736	-64597250.8806247	100076113.21934205	0.041478129713423836	0.34768432259781745	Other	Biogenic amines and polyamines
Imidazole propionate	-20004297.921489753	-84117057.905267	64112759.98377725	0.041478129713423836	0.34768432259781745	Other	SCFA and fermentation products
N oleoylethanolamine	6955441.379339416	-20278661.92698345	27234103.306322865	0.041478129713423836	0.34768432259781745	Other	Other
C20 3 CE	10738237.252664056	-9890678.260373555	20628915.51303761	0.041478129713423836	0.34768432259781745	Other	Lipids and fatty acids
Taurochenodesoxycholic acid	-1404533.5820279436	-10080789.22204424	8676255.640016297	0.041478129713423836	0.34768432259781745	Bile acids	Bile acids
Alpha Hydroxymetoprolol	13239790.215139799	8265330.86732706	4974459.347812739	0.041478129713423836	0.34768432259781745	Other	Host / drug / xenobiotic-like
Anserine	50176.63352599615	-483687.6755017305	533864.3090277266	0.041478129713423836	0.34768432259781745	Other	Amino acids
Alpha muricholate	163605647.66850698	11561939.678645229	152043707.98986176	0.05289808230984702	0.3506035687978233	Other	Bile acids

Metabolite	Median g1 (resp.)	Median g2 (non-resp.)	Effect	p	padj	Functional class (refined)	Functional class (granular)
Nicotinate	40757901.461759955	-3745422.07933107	44503323.541091025	0.05289808230984702	0.3506035687978233	Vitamins / cofactors	Vitamins and cofactors
Taurodeoxycholic acid	-26861663.7697755	-54169708.72177115	27308044.951995652	0.05289808230984702	0.3506035687978233	Other	Bile acids
Linoleoyl ethanolamide	-2227640.851417195	-13207907.978401799	10980267.126984604	0.05289808230984702	0.3506035687978233	Other	Lipids and fatty acids
Homovanillate	3020483.5258706864	-7149126.7592451945	10169610.28511588	0.05289808230984702	0.3506035687978233	Other	Phenolic / aromatic microbial metabolites
Beta sitosterol	11661056.096091751	6248641.610952515	5412414.485139236	0.05289808230984702	0.3506035687978233	Other	Other
Glycolithocholic acid	-158401.01095035725	1540250.48901033	-1698651.4999606872	0.05289808230984702	0.3506035687978233	Other	Bile acids
Palmitoylethanolamide	275222.055629266	-264568.926639825	539790.9822690911	0.05289808230984702	0.3506035687978233	Other	Lipids and fatty acids
Succite	-128379113.0723835	-651591040.9937994	523211927.9214159	0.06679594914889034	0.35918576429120275	Other	SCFA and fermentation products
Valine	434078285.41401196	13143814.049551852	420934471.3644601	0.06679594914889034	0.35918576429120275	Other	Amino acids
Glucurote	59476988.8502525	30487309.012321353	28989679.83793115	0.06679594914889034	0.35918576429120275	Other	Organic acids and TCA-related
Imidazolelactate	655798.38579809	-20960857.78821045	21616656.17400854	0.06679594914889034	0.35918576429120275	Other	SCFA and fermentation products
Acetylalanine	-4423517.231386885	-12028413.67809011	7604896.446703225	0.06679594914889034	0.35918576429120275	Amino acids	Amino acids
Hydroxyproline	1614022.8617729195	-3694904.5167403403	5308927.37851326	0.06679594914889034	0.35918576429120275	Other	Amino acids
Palmitoylethanolamide	4299515.244364585	-134843.4162310565	4434358.6605956415	0.06679594914889034	0.35918576429120275	Other	Lipids and fatty acids
C3 DC CH3 carnitine	-586052.8401185785	-4018062.550101025	3432009.7099824464	0.06679594914889034	0.35918576429120275	Other	Carnitine and methylated compounds
Riboflavin	384065.40363558347	26397.47635909455	357667.9272764889	0.06679594914889034	0.35918576429120275	Other	Vitamins and cofactors
Glutamate	829042662.449036	310941468.23159647	518101194.21743953	0.08306399482870072	0.37576569089174133	Other	Amino acids
Ketodeoxycholate	286015770.64685047	-168650255.821921	454666026.46877146	0.08306399482870072	0.37576569089174133	Other	Bile acids
Cholate	119605750.39754984	-210644934.994155	330250685.3917048	0.08306399482870072	0.37576569089174133	Other	Bile acids
Urate	161437013.2878245	142866247.86870348	18570765.419121027	0.08306399482870072	0.37576569089174133	Other	Other
Fumarate maleate	65368798.11321625	51593363.8222062	13775434.291010052	0.08306399482870072	0.37576569089174133	Other	Organic acids and TCA-related
Taurohyodeoxycholate tauroursodeoxycholate	1151459.43642714	-3681534.87137782	4832994.30780496	0.08306399482870072	0.37576569089174133	Other	Bile acids
C36 5 PC plasmalogen B	-266464.1411218314	763733.7480903965	-1030197.8892122279	0.08306399482870072	0.37576569089174133	Other	Lipids and fatty acids
Malate	622497340.0158525	482734185.3583895	139763154.65746295	0.10245636716224953	0.4358218603170316	Other	Organic acids and TCA-related
Pipecolic acid	151843253.8328655	190346777.9340315	-38503524.10116598	0.10245636716224953	0.4358218603170316	Other	Amino acids
Myristic acid	18390697.582656197	22091675.2690632	-3700977.6864070036	0.10245636716224953	0.4358218603170316	Other	Lipids and fatty acids
Tetrahydro beta carboline carboxylic acid	15740516.62353525	18732984.529160447	-2992467.905625198	0.10245636716224953	0.4358218603170316	Other	Tryptophan / indole metabolism
Aspartate	1346269116.823715	1082012072.535115	264257044.28859997	0.1246498599439776	0.467436974789916	Other	Amino acids
Cholesterol	527061583.133791	449910114.914845	77151468.21894604	0.1246498599439776	0.467436974789916	Other	Other
2 Aminobutyric Acid	7510628.826071395	-42573459.073125646	50084087.89919704	0.1246498599439776	0.467436974789916	SCFA / BCFA	Amino acids
Methylpyridonecarboxamide	69588120.5869692	92269451.04026185	-22681330.453292653	0.1246498599439776	0.467436974789916	Other	Vitamins and cofactors
Beta alanine	31775131.8581473	24213954.0765387	7561177.7816086	0.1246498599439776	0.467436974789916	Other	Amino acids
Xylose	1246231.4603405935	-4989598.3657328095	6235829.826073403	0.1246498599439776	0.467436974789916	Other	Sugars and carbohydrates
Didanosine	-909092.830259451	872441.68919497	-1781534.519454421	0.1246498599439776	0.467436974789916	Nucleosides / DNA-RNA	Nucleobases / nucleosides

Metabolite	Median g1 (resp.)	Median g2 (non-resp.)	Effect	p	padj	Functional class (refined)	Functional class (granular)
Uracil	487735098.6146445	415078479.2869985	72656619.32764602	0.15050635638870935	0.5106465663188353	Other	Nucleobases / nucleosides
2 hydroxyhexadecanoate	146701880.434509	195946511.9156205	-49244631.4811115	0.15050635638870935	0.5106465663188353	Other	Other
Glutamine	182670891.77527148	161937337.059377	20733554.71589446	0.15050635638870935	0.5106465663188353	Other	Amino acids
1 methylguanine	8192252.391100541	5354673.235249095	2837579.155851446	0.15050635638870935	0.5106465663188353	Other	Nucleobases / nucleosides
Phytosphingosine	8302432.467526754	5505084.652240595	2797347.8152861595	0.15050635638870935	0.5106465663188353	Other	Lipids and fatty acids
Undecanedicarboxylic acid	9516165.762434479	12264537.632724	-2748371.8702895213	0.15050635638870935	0.5106465663188353	Other	Other
Glycocholate	-329372.989817597	-2548033.7874459997	2218660.7976284027	0.15050635638870935	0.5106465663188353	Bile acids	Bile acids
7 methylguanine	-38202312.05108925	-127761706.5893014	89559394.53821215	0.1797026502908856	0.5886810957804873	Nucleosides / DNA-RNA	Nucleobases / nucleosides
C22 5 CE	11938095.02476755	9338763.92212218	2599331.1026453692	0.1797026502908856	0.5886810957804873	Other	Lipids and fatty acids
Oleic acid	1154873097.36888	2279684817.06181	-1124811719.69293	0.21288515406162467	0.6320028011204483	Host lipids / FA	Lipids and fatty acids
Cholestenone	1861842509.20758	2443878988.7788	-582036479.5712199	0.21288515406162467	0.6320028011204483	Other	Other
Arginine	-137252533.102725	-200634737.37966	63382204.27693501	0.21288515406162467	0.6320028011204483	Other	Amino acids
Threonine	334095945.03680897	298373529.868281	35722415.16852796	0.21288515406162467	0.6320028011204483	Other	Amino acids
Alanine	135860042.769418	109210204.9299175	26649837.8395005	0.21288515406162467	0.6320028011204483	Other	Amino acids
Betaine	162254159.106883	182396341.08739	-20142181.980507016	0.21288515406162467	0.6320028011204483	Other	Carnitine and methylated compounds
4 nitrophenol	31337769.0737673	40721171.28834215	-9383402.214574847	0.21288515406162467	0.6320028011204483	Other	Phenolic / aromatic microbial metabolites
Oxymetazoline	4007436.232673545	6206918.72650825	-2199482.4938347056	0.21288515406162467	0.6320028011204483	Other	Host / drug / xenobiotic-like
N acetylputrescine	942111184.2795556	744265579.9698765	197845604.30967903	0.24962292609351436	0.6775479422538248	Other	Biogenic amines and polyamines
Uridine	137946490.6665435	79170415.3210548	58776075.34548871	0.24962292609351436	0.6775479422538248	Other	Nucleobases / nucleosides
2 hydroxy 3 methylpentanoic acid leucinic acid	123773563.373275	106693640.7322565	17079922.641018495	0.24962292609351436	0.6775479422538248	Other	Lipids and fatty acids
C18 0 CE	28122870.47119705	24607844.36392875	3515026.1072683	0.24962292609351436	0.6775479422538248	Other	Lipids and fatty acids
Kynurenic acid	12674751.3275872	15507200.611370102	-2832449.2837829012	0.24962292609351436	0.6775479422538248	Other	Tryptophan / indole metabolism
Cystine	3808531.3544859653	2945888.648460835	862642.7060251301	0.24962292609351436	0.6775479422538248	Other	Amino acids
Urocanic acid	1018299924.37709	1215161321.022995	-196861396.64590502	0.29077784960137903	0.7206233664034176	Other	Tryptophan / indole metabolism
C34 1 DAG	137987766.33244848	153767339.708551	-15779573.376102507	0.29077784960137903	0.7206233664034176	Other	Lipids and fatty acids
Putrescine	92703492.54729456	78210366.67707245	14493125.870222107	0.29077784960137903	0.7206233664034176	Other	Biogenic amines and polyamines
Heptadecanoate	40059006.9303197	50417737.19634925	-10358730.266029552	0.29077784960137903	0.7206233664034176	Other	Lipids and fatty acids
N acetyl L arginine	1179836.5446954502	-4479406.105756	5659242.65045145	0.29077784960137903	0.7206233664034176	Other	Amino acids
13 docosenoate	4771832.349713075	6686344.796536544	-1914512.4468234694	0.29077784960137903	0.7206233664034176	Other	Lipids and fatty acids
Glycoursodeoxycholic acid	1522249.8149418754	549940.862487477	972308.9524543984	0.29077784960137903	0.7206233664034176	Bile acids	Bile acids
Fucose	1320772.03357764	935719.7470243269	385052.28655331314	0.29077784960137903	0.7206233664034176	Other	Sugars and carbohydrates
C36 2 DAG	1695213191.840255	1940532569.996145	-245319378.15589	0.33548804137039434	0.7710813854077612	Other	Lipids and fatty acids
Lysine	525957808.131565	311057314.70536304	214900493.42620194	0.33548804137039434	0.7710813854077612	Amino acids	Amino acids
C36 3 DAG	494002329.914976	590542035.0068365	-96539705.09186053	0.33548804137039434	0.7710813854077612	Other	Lipids and fatty acids

Metabolite	Median g1 (resp.)	Median g2 (non-resp.)	Effect	p	padj	Functional class (refined)	Functional class (granular)
Fructose glucose galactose	121752766.9733685	106729436.569823	15023330.403545499	0.33548804137039434	0.7710813854077612	Other	Sugars and carbohydrates
Pantothenate	136069427.7485735	125568180.481783	10501247.26679051	0.33548804137039434	0.7710813854077612	Other	Vitamins and cofactors
Pyroglutamic acid	140880740.385699	131320017.46579	9560722.919909	0.33548804137039434	0.7710813854077612	Other	Other
Acetytyrosine	56239803.676693	51728707.03545335	4511096.641239651	0.33548804137039434	0.7710813854077612	Other	Amino acids
C14 0 CE	12957034.92314045	14773608.6702813	-1816573.7471408509	0.33548804137039434	0.7710813854077612	Other	Lipids and fatty acids
Pyrocatechol	4698934.90201498	3100425.1294231154	1598509.7725918647	0.33548804137039434	0.7710813854077612	Aromatic / phenolic metabolism	Phenolic / aromatic microbial metabolites
Palmitic acid	339138135.796106	362413888.045334	-23275752.249228	0.3845076492135316	0.8561303127020039	Other	Lipids and fatty acids
Thiamine	345024184.5427555	359006813.641469	-13982629.098713517	0.3845076492135316	0.8561303127020039	Other	Vitamins and cofactors
1 2 3 4 Tetrahydro b carboline 1 3 dicarboxylic acid	24615699.83185725	25907659.39407985	-1291959.5622226	0.3845076492135316	0.8561303127020039	Other	Tryptophan / indole metabolism
C36 4 PC B	5561495.650505984	6393317.371606765	-831821.7211007811	0.3845076492135316	0.8561303127020039	Other	Lipids and fatty acids
Oleamide	64029598.02585075	40193213.49931525	23836384.526535504	0.43708252531781944	0.8711085294795702	Other	Lipids and fatty acids
Sphinganine	135484147.04317552	111724413.5902065	23759733.452969015	0.43708252531781944	0.8711085294795702	Other	Lipids and fatty acids
Glutamate	348799521.74813855	332270922.156584	16528599.591554523	0.43708252531781944	0.8711085294795702	Other	Amino acids
4 guanidinobutanoic acid	-8313830.91588579	-22979686.699920148	14665855.784034356	0.43708252531781944	0.8711085294795702	Amino acids	Other
ADMA SDMA	-45135271.7065869	-56732747.15836255	11597475.451775655	0.43708252531781944	0.8711085294795702	Other	Carnitine and methylated compounds
C34 0 DAG	85295586.3577989	82101082.8367342	3194503.5210646987	0.43708252531781944	0.8711085294795702	Other	Lipids and fatty acids
C22 6 CE	17967556.8092165	14907576.3811596	3059980.4280568995	0.43708252531781944	0.8711085294795702	Other	Lipids and fatty acids
Lactose	12801592.1051035	9900275.119063465	2901316.9860400353	0.43708252531781944	0.8711085294795702	Other	Sugars and carbohydrates
Alpha ketoisovalerate	-8881562.327471904	-11101275.949141216	2219713.621669311	0.43708252531781944	0.8711085294795702	BCAA / branched AA	Other
Stearoyl ethanolamide	21425488.85093715	19351116.1308242	2074372.7201129496	0.43708252531781944	0.8711085294795702	Other	Lipids and fatty acids
Tetradecanedioate	7021367.928620715	8079999.412607195	-1058631.4839864802	0.43708252531781944	0.8711085294795702	Other	Other
2 hydroxyglutarate	50938397.46940685	50438279.44849705	500118.0209098011	0.43708252531781944	0.8711085294795702	Other	Organic acids and TCA-related
C32 1 PC	3720990.3563731797	3225245.13167476	495745.2246984197	0.43708252531781944	0.8711085294795702	Other	Lipids and fatty acids
Tartarate	1037756.366372175	652188.8392073625	385567.52716481255	0.43708252531781944	0.8711085294795702	Organic acids	Organic acids and TCA-related
Ornithine	15842835.34811765	16200423.4803806	-357588.13226295076	0.43708252531781944	0.8711085294795702	Other	Amino acids
Hypoxanthine	3572548389.676115	3253080487.498525	319467902.1775899	0.49364361129067014	0.8961046446996241	Other	Nucleobases / nucleosides
Ascorbate	421886003.05815053	351485108.420851	70400894.63729954	0.49364361129067014	0.8961046446996241	Other	Vitamins and cofactors
L phenyllactic Acid	134518831.6696725	116008217.378535	18510614.291137487	0.49364361129067014	0.8961046446996241	Other	Phenolic / aromatic microbial metabolites
C18 1 CE	198258190.736709	189196563.8816285	9061626.855080485	0.49364361129067014	0.8961046446996241	Other	Lipids and fatty acids
9 10 diHOME	31988810.298005648	26573438.9408113	5415371.357194349	0.49364361129067014	0.8961046446996241	Other	Other
C20 4 CE	143560612.49773848	141261838.2163215	2298774.2814169824	0.49364361129067014	0.8961046446996241	Other	Lipids and fatty acids
Bilirubin	-5008837.683190345	-3469869.3785881	-1538968.3046022449	0.49364361129067014	0.8961046446996241	Other	Other
C36 1 PC	37427965.59636575	36539859.07329525	888106.5230704993	0.49364361129067014	0.8961046446996241	Other	Lipids and fatty acids
Palmitoyl glycerol	37326297.06010215	37954042.50775145	-627745.4476493001	0.49364361129067014	0.8961046446996241	Other	Lipids and fatty acids

Metabolite	Median g1 (resp.)	Median g2 (non-resp.)	Effect	p	padj	Functional class (refined)	Functional class (granular)
Salicylate	-2673649.35877015	-2257390.53547809	-416258.8232920603	0.49364361129067014	0.8961046446996241	Xenobiotic / drug	Host / drug / xenobiotic-like
Nervonic acid	4263504.9433444105	4629216.20977296	-365711.2664285498	0.49364361129067014	0.8961046446996241	Other	Lipids and fatty acids
Phenyllactate	1115972243.4951239	932725350.6255391	183246892.8695848	0.5532212885154062	0.9345301131909393	Aromatic / phenolic metabolism	SCFA and fermentation products
Pentadecanoate	176933646.44035375	226702858.3668895	-49769211.926535755	0.5532212885154062	0.9345301131909393	Host lipids / FA	Lipids and fatty acids
Histamine	324029419.195701	340564521.84515	-16535102.64944899	0.5532212885154062	0.9345301131909393	Other	Biogenic amines and polyamines
Serine	152703490.031232	139515569.8371765	13187920.194055498	0.5532212885154062	0.9345301131909393	Other	Amino acids
4 hydroxystyrene	53016789.03348015	44539870.528085	8476918.505395152	0.5532212885154062	0.9345301131909393	Aromatic / phenolic metabolism	Phenolic / aromatic microbial metabolites
C18 3 CE	-9289792.97004765	-16951411.828018002	7661618.857970351	0.5532212885154062	0.9345301131909393	Host lipids / FA	Lipids and fatty acids
Lactate	-9742451.20539321	-14945659.0398672	5203207.83447399	0.5532212885154062	0.9345301131909393	Other	SCFA and fermentation products
C16 1 CE	41067444.3595186	38824245.5899632	2243198.769555405	0.5532212885154062	0.9345301131909393	Other	Lipids and fatty acids
Eicosenoate	93747118.15097865	95965347.6450209	-2218229.4940422475	0.5532212885154062	0.9345301131909393	Other	Lipids and fatty acids
C20 5 LPC	2443187.76554281	2989161.78264509	-545974.0171022802	0.5532212885154062	0.9345301131909393	Other	Lipids and fatty acids
Methionine	267021356.10768798	228845890.95805502	38175465.14963296	0.6164619694031459	0.9345301131909393	Other	Amino acids
Hydrocinnamic acid	4211369.8335086	-27323084.8091045	31534454.642613098	0.6164619694031459	0.9345301131909393	Other	Phenolic / aromatic microbial metabolites
Tryptophan	247796630.257586	227568146.504343	20228483.753243	0.6164619694031459	0.9345301131909393	Other	Amino acids
C18 0e MAG	990439358.7758856	974306137.8971475	16133220.878738046	0.6164619694031459	0.9345301131909393	Other	Lipids and fatty acids
Leucine	762230117.3422074	748721425.453946	13508691.888261437	0.6164619694031459	0.9345301131909393	Other	Amino acids
Acetylcholine	20982978.02191795	33858129.71550005	-12875151.693582099	0.6164619694031459	0.9345301131909393	Other	Lipids and fatty acids
1 2 3 4 Tetrahydro 1 methyl beta carboline 3 carboxylic acid	-7457987.38523072	-15298248.8161244	7840261.43089368	0.6164619694031459	0.9345301131909393	Aromatic / phenolic metabolism	Tryptophan / indole metabolism
Sphingosine	144782721.23593348	151544456.348693	-6761735.112759531	0.6164619694031459	0.9345301131909393	Other	Lipids and fatty acids
Guanine	2292889.1652719	-270965.2950826195	2563854.4603545195	0.6164619694031459	0.9345301131909393	Other	Nucleobases / nucleosides
Proline	327863174.069748	325403054.7875985	2460119.2821494937	0.6164619694031459	0.9345301131909393	Other	Amino acids
Serine	74182520.32433845	71977991.7360281	2204528.588310346	0.6164619694031459	0.9345301131909393	Other	Amino acids
NMMA	-1993754.1675509824	-4034489.86820989	2040735.7006589076	0.6164619694031459	0.9345301131909393	Other	Carnitine and methylated compounds
PGE2 d4	41819444.2705297	43797286.54318825	-1977842.2726585492	0.6164619694031459	0.9345301131909393	Other	Host / drug / xenobiotic-like
C34 1 PC	11616233.478174046	9691421.539132645	1924811.9390414003	0.6164619694031459	0.9345301131909393	Other	Lipids and fatty acids
Stearic acid	440346322.17132	441526671.048067	-1180348.8767469525	0.6164619694031459	0.9345301131909393	Other	Lipids and fatty acids
C18 1 LPC	2540971.05328108	3158284.905743365	-617313.8524622852	0.6164619694031459	0.9345301131909393	Other	Lipids and fatty acids
C18 2 LPC	3844917.63946715	4409208.796249	-564291.1567818504	0.6164619694031459	0.9345301131909393	Other	Lipids and fatty acids
Palmitoleic acid	11208877.747916602	11601139.01568855	-392261.2677719481	0.6164619694031459	0.9345301131909393	Other	Lipids and fatty acids
C18 2 carnitine	1842986.259936485	1580392.58385805	262593.67607843503	0.6164619694031459	0.9345301131909393	Other	Carnitine and methylated compounds
C22 4 CE	9349072.928920096	9213202.101959966	135870.82696012966	0.6164619694031459	0.9345301131909393	Other	Lipids and fatty acids
Creatinine	1447305485.82931	1588159254.06377	-140853768.23446012	0.6819650937297996	0.9718002585649645	Other	Other
Taurine	651404004.7544194	770728626.6530415	-119324621.89862204	0.6819650937297996	0.9718002585649645	Other	Other

Metabolite	Median g1 (resp.)	Median g2 (non-resp.)	Effect	p	padj	Functional class (refined)	Functional class (granular)
Tyrosine	1567563624.66139	1496210547.17238	71353077.4890101	0.6819650937297996	0.9718002585649645	Other	Amino acids
Docosahexaenoic acid	278924550.8275705	300482256.93018055	-21557706.10261005	0.6819650937297996	0.9718002585649645	Other	Lipids and fatty acids
C24 0 PC	166861169.96663648	171521684.64305001	-4660514.676413536	0.6819650937297996	0.9718002585649645	Other	Lipids and fatty acids
Caffeine	-18669689.745382752	-21151298.99646205	2481609.2510792986	0.6819650937297996	0.9718002585649645	Other	Host / drug / xenobiotic-like
C20 5 CE	32709000.94808485	31314666.81642005	1394334.1316648014	0.6819650937297996	0.9718002585649645	Other	Lipids and fatty acids
Cytosine	-4884979.023569331	-6043205.532798694	1158226.5092293639	0.6819650937297996	0.9718002585649645	Other	Nucleobases / nucleosides
C22 1 SM	11662652.290869351	12424377.446760349	-761725.1558909975	0.6819650937297996	0.9718002585649645	Other	Lipids and fatty acids
C34 0 PE	-1472955.3903858	-984310.312137173	-488645.078248627	0.6819650937297996	0.9718002585649645	Other	Lipids and fatty acids
C36 2 PC plasmalogen	1040903.8664021385	1028345.150304477	12558.716097661527	0.6819650937297996	0.9718002585649645	Other	Lipids and fatty acids
Trigonelline	408611491.036179	426131484.27262497	-17519993.236445963	0.7502693385046326	0.98085670400835	Other	Vitamins and cofactors
Tryptophan	248139854.16435498	235853398.3531255	12286455.811229467	0.7502693385046326	0.98085670400835	Other	Amino acids
Creatine	440397033.241979	428702228.623216	11694804.61876303	0.7502693385046326	0.98085670400835	Other	Other
Dimethyllysine	207425566.13476402	200626810.13519	6798755.999574006	0.7502693385046326	0.98085670400835	Other	Amino acids
Butyrobetaine	4046745062.917355	4053010421.44731	-6265358.52995491	0.7502693385046326	0.98085670400835	SCFA / BCFA	Carnitine and methylated compounds
C18 0 MAG	332287457.21722245	326514551.0789305	5772906.138291955	0.7502693385046326	0.98085670400835	Other	Lipids and fatty acids
C18 0 SM	61941856.0410785	66588159.21652585	-4646303.175447352	0.7502693385046326	0.98085670400835	Other	Lipids and fatty acids
C16 0 LPE	71815211.77990276	75970193.53751075	-4154981.7576079965	0.7502693385046326	0.98085670400835	Other	Lipids and fatty acids
C14 0 SM	55463859.2699922	51402866.799695596	4060992.4702966064	0.7502693385046326	0.98085670400835	Other	Lipids and fatty acids
P hydroxyphenylacetate	52847107.83615565	49424055.56834445	3423052.2678112015	0.7502693385046326	0.98085670400835	Other	Phenolic / aromatic microbial metabolites
Deoxyinosine	75907249.69331911	73378207.59797695	2529042.0953421593	0.7502693385046326	0.98085670400835	Other	Nucleobases / nucleosides
Methionine sulfoxide	42001277.629026055	39548820.7436814	2452456.8853446543	0.7502693385046326	0.98085670400835	Other	Amino acids
C24 0 SM	86132831.8525067	84173724.094601	1959107.7579056919	0.7502693385046326	0.98085670400835	Other	Lipids and fatty acids
C34 2 PC	-3942906.9931586212	-2110401.3906833753	-1832505.602475246	0.7502693385046326	0.98085670400835	Other	Lipids and fatty acids
Phenylacetate	-4923643.3662422	-4807635.4871863555	-116007.87905584462	0.7502693385046326	0.98085670400835	Other	Phenolic / aromatic microbial metabolites
Piperine	-1044843759.863545	-2738179938.564005	1693336178.70046	0.8200818789054083	0.9861744113419466	Xenobiotic / drug	Lipids and fatty acids
Atenolol	357809433.6930605	492423487.193377	-134614053.5003165	0.8200818789054083	0.9861744113419466	Xenobiotic / drug	Host / drug / xenobiotic-like
Isoleucine	480773102.745749	432597073.24881554	48176029.49693346	0.8200818789054083	0.9861744113419466	Other	Amino acids
Orotate	249463671.2637025	281126865.21192455	-31663193.94822204	0.8200818789054083	0.9861744113419466	Other	Organic acids and TCA-related
Trimethyllysine	122152810.2068795	143392799.3892265	-21239989.182347	0.8200818789054083	0.9861744113419466	Carnitine / TMAO axis	Amino acids
C20 0 LPE	136823204.713821	144249601.894083	-7426397.180261999	0.8200818789054083	0.9861744113419466	Other	Lipids and fatty acids
Oxalate	98714020.27063316	102304130.29659939	-3590110.025966227	0.8200818789054083	0.9861744113419466	Other	Organic acids and TCA-related
Eicosapentaenoic acid	110887449.34896901	107479249.58407399	3408199.764895022	0.8200818789054083	0.9861744113419466	Other	Lipids and fatty acids
C36 0 DAG	55498719.13912965	57000480.3322272	-1501761.1930975467	0.8200818789054083	0.9861744113419466	Other	Lipids and fatty acids
Glycerate	137226523.760387	138394699.98505902	-1168176.2246720195	0.8200818789054083	0.9861744113419466	Other	Organic acids and TCA-related

Metabolite	Median g1 (resp.)	Median g2 (non-resp.)	Effect	p	padj	Functional class (refined)	Functional class (granular)
3 methylhistidine	20188593.27426865	19106282.38988425	1082310.8843844011	0.8200818789054083	0.9861744113419466	Other	Amino acids
C18 carnitine	13647299.49093645	12980909.81431305	666389.6766234003	0.8200818789054083	0.9861744113419466	Other	Carnitine and methylated compounds
Triethanolamine	8636401.95636179	9115857.20045092	-479455.2440891303	0.8200818789054083	0.9861744113419466	Other	Other
C18 1 LPC plasmalogen	24853599.99917925	24732242.90942065	121357.08975860104	0.8200818789054083	0.9861744113419466	Other	Lipids and fatty acids
C16 1 LPC plasmalogen	4333905.01930129	4252418.805386946	81486.21391434409	0.8200818789054083	0.9861744113419466	Other	Lipids and fatty acids
C14 carnitine	484397.3676951005	455891.5626469275	28505.805048173002	0.8200818789054083	0.9861744113419466	Other	Carnitine and methylated compounds
C4 carnitine	186261.563318072	200125.612705715	-13864.049387642997	0.8200818789054083	0.9861744113419466	Other	Carnitine and methylated compounds
Phenylalanine	2025494213.1117148	2081126334.51361	-55632121.401895046	0.8916181857358327	0.9986249045072576	Other	Amino acids
C16 0 LPC	463733892.455721	494263211.361765	-30529318.906044006	0.8916181857358327	0.9986249045072576	Other	Lipids and fatty acids
Taurine	79057593.4845478	60973486.52651335	18084106.95803444	0.8916181857358327	0.9986249045072576	Other	Other
C18 3 LPC	156488635.579637	166965780.646622	-10477145.066985011	0.8916181857358327	0.9986249045072576	Other	Lipids and fatty acids
Thymine	228962049.96946	222127308.8503935	6834741.119066507	0.8916181857358327	0.9986249045072576	Other	Nucleobases / nucleosides
LysoPC O 18 0	323058482.67425096	318519827.198505	4538655.475745976	0.8916181857358327	0.9986249045072576	Other	Lipids and fatty acids
Adrenic acid	96149830.19880305	100564490.38917431	-4414660.19037126	0.8916181857358327	0.9986249045072576	Other	Lipids and fatty acids
2 hydroxyphenethylamine	38439195.8220075	34214065.5493519	4225130.272655599	0.8916181857358327	0.9986249045072576	Other	Biogenic amines and polyamines
Threonine	63605758.78360075	60683873.8703569	2921884.913243845	0.8916181857358327	0.9986249045072576	Other	Amino acids
Eicosapentaenoic acid	176768530.3043155	174462378.250593	2306152.053722501	0.8916181857358327	0.9986249045072576	Other	Lipids and fatty acids
C34 1 PC plasmalogen A	24143541.9624875	25748169.56267845	-1604627.6001909487	0.8916181857358327	0.9986249045072576	Other	Lipids and fatty acids
Citrate	30695500.853752196	29522705.4804245	1172795.3733276948	0.8916181857358327	0.9986249045072576	Other	Organic acids and TCA-related
C18 1 carnitine	11519408.64300505	10692094.929613698	827313.7133913524	0.8916181857358327	0.9986249045072576	Other	Carnitine and methylated compounds
7 Hexadecenoic acid methyl ester	11018010.18764087	11747480.41174235	-729470.2241014801	0.8916181857358327	0.9986249045072576	Other	Lipids and fatty acids
1 methylnicotinamide	7999276.306863405	7537313.04858571	461963.2582776947	0.8916181857358327	0.9986249045072576	Other	Vitamins and cofactors
C16 carnitine	7696297.64064917	7513705.317534136	182592.32311503403	0.8916181857358327	0.9986249045072576	Other	Carnitine and methylated compounds
Arachidonic acid	1773615409.118335	1887726909.38116	-114111500.26282501	0.9635854341736694	0.9986249045072576	Other	Lipids and fatty acids
Caprylic acid	-176226849.459744	-79410053.0542994	-96816796.4054446	0.9635854341736694	0.9986249045072576	Host lipids / FA	Lipids and fatty acids
Carnitine	-88736504.33897837	-104038793.75372285	15302289.414744481	0.9635854341736694	0.9986249045072576	Carnitine / TMAO axis	Carnitine and methylated compounds
C24 1 SM	326742749.015652	322687606.6226765	4055142.392975509	0.9635854341736694	0.9986249045072576	Other	Lipids and fatty acids
12 13 diHOME	45552513.66290035	43146411.736607194	2406101.926293157	0.9635854341736694	0.9986249045072576	Host lipids / FA	Other
Acesulfame	1653074.338485975	2995354.65840616	-1342280.3199201848	0.9635854341736694	0.9986249045072576	Other	Lipids and fatty acids
Methylbutyric aid Valeric Isovaleric	52740882.1500798	53974154.190228805	-1233272.0401490033	0.9635854341736694	0.9986249045072576	SCFA / BCFA	SCFA and fermentation products
C16 0 SM	16527135.99868815	15304683.56162815	1222452.4370600004	0.9635854341736694	0.9986249045072576	Other	Lipids and fatty acids
C30 0 DAG	-7955400.801013459	-7033185.96143076	-922214.8395826994	0.9635854341736694	0.9986249045072576	Other	Lipids and fatty acids
C18 2 CE	600946045.461956	561882530.6859839	39063514.77597213	0.9635854341736696	0.9986249045072576	Other	Lipids and fatty acids
Docosapentaenoic acid	130832044.194111	148281903.9573655	-17449859.76325451	0.9635854341736696	0.9986249045072576	Other	Lipids and fatty acids

Metabolite	Median g1 (resp.)	Median g2 (non-resp.)	Effect	p	padj	Functional class (refined)	Functional class (granular)
Tyrosine	624984977.041141	639495743.7214329	-14510766.680291891	0.9635854341736696	0.9986249045072576	Other	Amino acids
Eicosatrienoic acid	94936859.4702518	107168400.0443795	-12231540.574127704	0.9635854341736696	0.9986249045072576	Other	Other
C18 0 LPC	423740458.652676	411977447.2311585	11763011.421517491	0.9635854341736696	0.9986249045072576	Other	Lipids and fatty acids
Docosapentaenoate	206138113.951767	215154771.19312352	-9016657.241356522	0.9635854341736696	0.9986249045072576	Other	Lipids and fatty acids
N acetylhistidine	146436512.1701895	141099745.35255247	5336766.817637026	0.9635854341736696	0.9986249045072576	Other	Amino acids
C16 0 CE	80848692.8229312	84247462.1558696	-3398769.332938403	0.9635854341736696	0.9986249045072576	Other	Lipids and fatty acids
C18 0 LPC	25508258.25627455	24115058.86298155	1393199.3932930008	0.9635854341736696	0.9986249045072576	Other	Lipids and fatty acids
Citrulline	512097196.28075004	510941864.95009446	1155331.3306555748	0.9635854341736696	0.9986249045072576	Other	Amino acids
C16 1 SM	43370384.72042775	42994121.306870654	376263.4135570973	0.9635854341736696	0.9986249045072576	Other	Lipids and fatty acids
C22 1 MAG	3546758.452303705	3860519.374132575	-313760.9218288702	0.9635854341736696	0.9986249045072576	Other	Lipids and fatty acids
C20 carnitine	2166851.52183748	2237359.92318265	-70508.4013451701	0.9635854341736696	0.9986249045072576	Other	Carnitine and methylated compounds
C16 0 Ceramide d18	180357387.96972448	201465440.7387425	-21108052.769018024	1	1	Other	Lipids and fatty acids
Caproic acid	28467334.98747485	7667852.919301525	20799482.068173327	1	1	Other	SCFA and fermentation products
Threosphingosine	195487168.467866	178588588.5215835	16898579.946282506	1	1	Other	Lipids and fatty acids
Erythronic acid	123736471.29305601	113872924.93833551	9863546.354720503	1	1	Other	Organic acids and TCA-related
Histidine	73302361.3200349	68956462.86022535	4345898.459809557	1	1	Other	Amino acids
C22 0 SM	71631399.1995962	74878116.6847433	-3246717.4851471037	1	1	Other	Lipids and fatty acids
C38 5 DAG	16380415.239214052	18618201.2159393	-2237785.9767252468	1	1	Other	Lipids and fatty acids
Carnosine	7978924.87917907	7715829.031360175	263095.8478188943	1	1	Other	Other

Table S6. Differential abundance of collapsed functional metabolite categories between the two response groups (g1, g2) at T0 (Wilcoxon rank-sum test, log2 fold-change, Benjamini-Hochberg FDR).

Feature	Median g1 (resp.)	Median g2 (non-resp.)	log2FC	p	padj
Butyrate	0.94	0	16.52	0.179	1
Polyphenols	0	0.307	-14.90	0.259	1
Tryptamine	14.2	25.2	-0.82	0.282	1
Propionate	11.1	14.6	-0.39	0.282	1
Lactate	14.1	18	-0.35	0.282	1
D-lactate	0.696	1.02	-0.55	0.328	1
Indoleacetic acid	0	0	0.00	0.332	1
Mucolysis	83.1	63.9	0.38	0.345	1
B-glucuronidase	3.5	1.86	0.91	0.414	1
Pyruvate	78.1	118	-0.59	0.414	1
Acetate	34.2	42.1	-0.30	0.414	1
Trimethylamine	1.92	1.14	0.76	0.476	1
Proteolysis	5.64	10.5	-0.89	0.491	1
Indole	15.1	26.1	-0.79	0.491	1
Succinate	10.4	19.6	-0.92	0.573	1
Serotonin	25.9	44.9	-0.79	0.573	1
Fumarate	40.8	53.8	-0.40	0.573	1
Hydrogen sulphide	0.772	1.17	-0.60	0.695	1
Methane	1.2	0.92	0.39	0.73	1
Cadaverine	1.91	1.98	-0.05	0.744	1
Spermidine	4.56	5.65	-0.31	0.755	1
Vitamin K2	14.5	12.5	0.21	0.755	1
Putrescine	8.87	9.69	-0.13	0.755	1
Indolepropionic acid	4.55	3.62	0.33	0.852	1
Secondary bile acids	3.28	2.83	0.21	0.852	1
GABA	2.92	4.62	-0.66	0.896	1
B-group vitamins	24.6	18.1	0.44	0.95	1
Lipopolysaccharide	13.2	15.4	-0.22	0.95	1
Histamine	7.95	8.77	-0.14	0.95	1
Ethanol	9.66	10.4	-0.10	0.95	1
L-lactate	2.41	2.79	-0.21	1	1
Sulfatase	4.22	4.37	-0.05	1	1

Feature	Median g1 (resp.)	Median g2 (non-resp.)	log2FC	p	padj
Gluten degradation	0	0	0.00		

Table S7. Logistic regression model outputs for chemotherapy-response prediction.

Panel A. Model comparison

Model	Predictors	AIC	Null dev.	Resid. dev.	Pseudo-R2
Multivariable	Predicted butyrate + Stage (III vs IV)	20.58	19.12	14.58	0.237
Univariable	Predicted butyrate only	21.91	19.12	17.91	n/a
Univariable	Stage (III vs IV) only	21.37	19.12	17.37	n/a
Null	Intercept only	21.12	19.12	19.12	n/a

Panel B. Predictor estimates

Model	Predictor	Estimate (log-odds)	OR	95% CI low	95% CI high	P
Multivariable	Predicted butyrate	1.671	5.32	0.77	80.94	0.139
Multivariable	Stage IV vs III	-2.848	0.058	0.0004	1.20	0.139
Univariable	Predicted butyrate	0.888	2.43	n/a	n/a	0.291
Univariable	Stage IV vs III	-1.609	0.20	n/a	n/a	0.217

Panel C. Sample characteristics

	Responders	Non-responders	Total
N	6	8	14
Stage III	5	4	9
Stage IV	1	4	5

Table S8. PERMANOVA of baseline (T0) faecal species composition as a function of the subsequently assigned chemotherapy regimen. Bray-Curtis dissimilarities on species-level relative abundances; 999 permutations. Only the two main regimen classes were compared (FOLFIRINOX-based, n = 7; Gemcitabine-based, n = 6); the PAX-G patient (n = 1) and one patient with an unspecified regimen were excluded. Composition did not differ significantly between groups.

Source	Df	SumOfSqs	R2	pseudo-F	Pr(>F)
Chemotherapy regimen	1	0.2804	0.077	0.920	0.509
Residual	11	3.3508	0.923	n/a	n/a
Total	12	3.6312	1.000	n/a	n/a

Table S9. Baseline demographic, clinical, nutritional and lifestyle characteristics of responders versus non-responders. All 15 enrolled patients (6 responders, 9 non-responders). Continuous variables are median [interquartile range] (Mann-Whitney U test); categorical variables are n (%) (Fisher's exact test). No variable differed significantly between groups (all p > 0.05; the largest difference was in CA19-9, p = 0.088, consistent with higher tumour burden in non-responders).

Characteristic	Responders (n = 6)	Non-responders (n = 9)	p-value
Age, years	67.0 [63.0-69.5]	64.0 [61.0-70.0]	0.953
Sex, male, n (%)	4 (67%)	6 (67%)	1.000
Stage IV, n (%)	1 (17%)	5 (56%)	0.287
BMI, kg/m2	25.0 [23.9-25.9]	26.0 [24.2-27.9]	0.479
CA19-9, U/mL	152.5 [140.0-1307.2]	1724.0 [597.0-6056.0]	0.088
NLR	2.9 [1.9-3.9]	3.8 [3.1-4.3]	0.224
CRP, mg/L	5.8 [1.3-8.8]	2.0 [0.9-24.3]	1.000
Albumin, g/L	39.8 [38.1-43.2]	42.1 [38.1-43.5]	0.860
PNI	50.1 [49.8-52.3]	49.0 [45.2-49.6]	0.181
SMI, kg/m2	9.9 [8.0-10.1]	10.7 [9.8-11.8]	0.240
Mediterranean-diet adherence (MEDAS group, 1-3)	2.0 [1.2-2.0]	2.0 [2.0-2.0]	0.819
Malnutrition risk, n (%)	4/6 (67%)	5/8 (62%)	1.000
Sarcopenia, n (%)	4/6 (67%)	3/9 (33%)	0.315
Antibiotics (prev. 30 d), n (%)	1/6 (17%)	1/9 (11%)	1.000
Proton pump inhibitors, n (%)	1/6 (17%)	4/9 (44%)	0.580
Supplements/probiotics, n (%)	3/6 (50%)	5/9 (56%)	1.000
Any concomitant medication, n (%)	3/6 (50%)	8/9 (89%)	0.235

Figure S1. Study workflow. Patient recruitment, faecal sampling at diagnosis (T0) before chemotherapy, whole-genome shotgun metagenomic sequencing (NovaSeq 6000Dx, single batch), the bioinformatic pipeline (Kraken2 taxonomy; MEGAHIT/FragGeneScan/DIAMOND functional profiling and metabolite prediction), statistical analysis and outcome assessment (surgical eligibility; 6 responders vs. 9 non-responders).

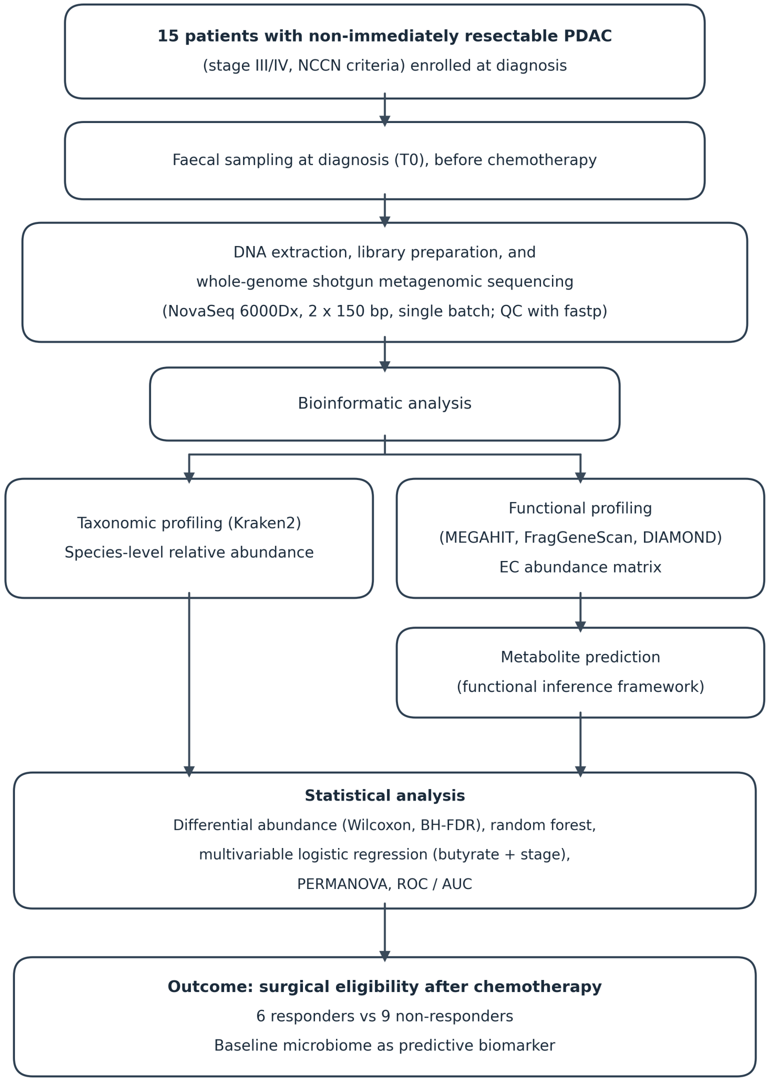

Figure S2. ROC curve for the multivariable model. Receiver operating characteristic curve for the multivariable logistic regression model (predicted butyrate + disease stage) predicting surgical eligibility. AUC = 0.78 (95% CI 0.52-1.00, DeLong); sensitivity 0.67, specificity 0.88 at the Youden-optimal threshold (n = 14). Reported for descriptive purposes given the small sample size and absence of an independent validation set.

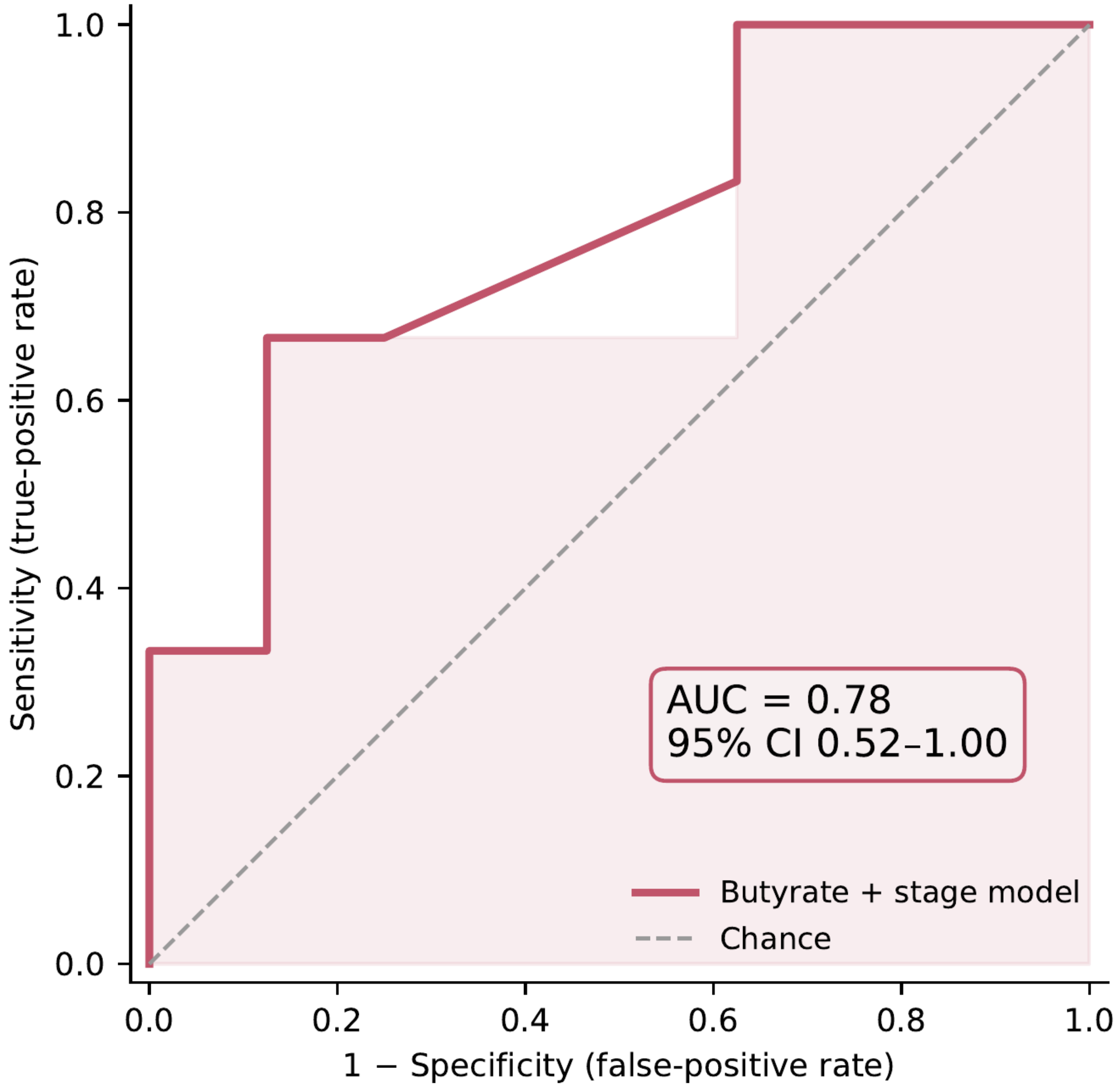

Supplement: Supplementary file 1 [file metabolites-16-00720-s001.zip › metabolites-4532032-supplementary.pdf]
